# Enabling late-stage drug diversification by high-throughput experimentation with geometric deep learning

**DOI:** 10.1038/s41557-023-01360-5

**Published:** 2023-11-23

**Authors:** David F. Nippa, Kenneth Atz, Remo Hohler, Alex T. Müller, Andreas Marx, Christian Bartelmus, Georg Wuitschik, Irene Marzuoli, Vera Jost, Jens Wolfard, Martin Binder, Antonia F. Stepan, David B. Konrad, Uwe Grether, Rainer E. Martin, Gisbert Schneider

**Affiliations:** 1 Roche Pharma Research and Early Development (pRED), Roche Innovation Center Basel, F. Hoffmann-La Roche Ltd., Basel, Switzerland; 2 Department of Pharmacy, Ludwig-Maximilians-Universität München, Munich, Germany; 3 Department of Chemistry and Applied Biosciences, ETH Zurich, Zurich, Switzerland; 4 Process Chemistry and Catalysis (PCC), F. Hoffmann-La Roche Ltd., Basel, Switzerland; 5 ETH Singapore SEC Ltd, Singapore, Singapore

**Keywords:** Machine learning, Lead optimization, Synthetic chemistry methodology, Chemical engineering, Automation

## Abstract

Late-stage functionalization is an economical approach to optimize the properties of drug candidates. However, the chemical complexity of drug molecules often makes late-stage diversification challenging. To address this problem, a late-stage functionalization platform based on geometric deep learning and high-throughput reaction screening was developed. Considering borylation as a critical step in late-stage functionalization, the computational model predicted reaction yields for diverse reaction conditions with a mean absolute error margin of 4–5%, while the reactivity of novel reactions with known and unknown substrates was classified with a balanced accuracy of 92% and 67%, respectively. The regioselectivity of the major products was accurately captured with a classifier *F*-score of 67%. When applied to 23 diverse commercial drug molecules, the platform successfully identified numerous opportunities for structural diversification. The influence of steric and electronic information on model performance was quantified, and a comprehensive simple user-friendly reaction format was introduced that proved to be a key enabler for seamlessly integrating deep learning and high-throughput experimentation for late-stage functionalization.

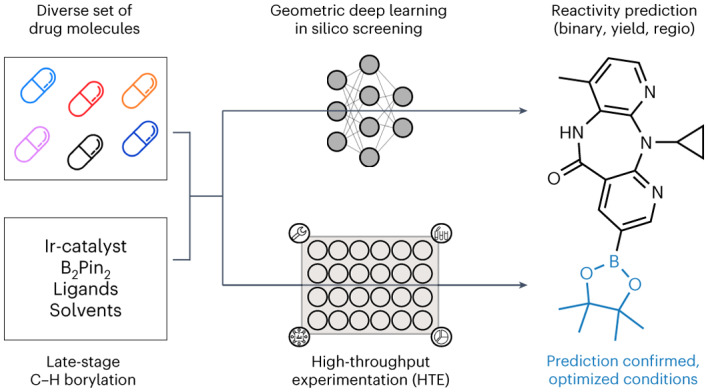

## Abstract (web summary)

Late-stage functionalization of complex drug molecules is challenging. To address this problem, a discovery platform based on geometric deep learning and high-throughput experimentation was developed. The computational model predicts binary reaction outcome, reaction yield and regioselectivity with low error margins, enabling the functionalization of complex molecules without de novo synthesis.

## Main

**Fig. 1:**
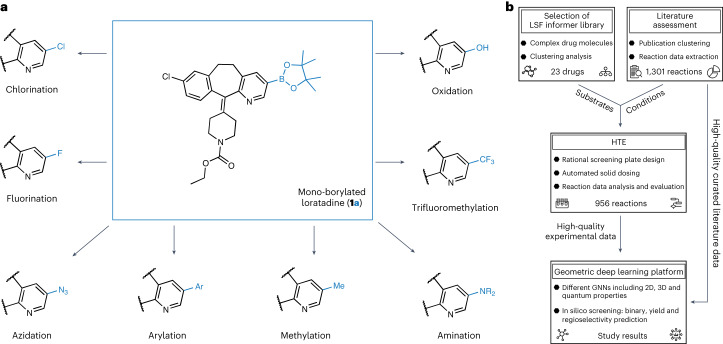
Borylation diversification opportunities and research overview of the study. **a**, Late-stage borylation of a drug molecule. The example illustrates mono-borylated Loratadine (**1a**), which can be accessed through borylation of the drug Loratadine (**1**). Borylation provides the opportunity for rapid and broad diversification, aiming to study structure–activity relationships and improve pharmacokinetic and pharmacodynamic properties. Note that the eight potential post-functionalization modifications shown are for demonstration purposes only; these transformations were not carried out in the presented research. **b**, Overview of the research study. A comprehensive literature study provided a manually curated, high-quality literature dataset containing 1,301 reactions extracted from 38 publications. The dataset was used to identify suitable borylation reaction conditions for HTE and used for machine learning. The LSF informer library resulted from a cluster analysis of 1,174 approved drug molecules. In total, 23 drugs from the LSF informer library, 12 relevant fragments and 5 simple substrates were subjected to HTE to deliver 956 experimental data points. Both experimental and literature data provided the basis for geometric deep learning using different GNNs, including 2D and 3D information and atomic partial charges. Prediction models for substrate reactivity, reaction yields and regioselectivity were developed, and the results are shared in this study.

Structural novelty and complexity render the synthesis of chemical target structures challenging when aiming to establish structure–activity relationships in medicinal chemistry^1^. Structure–activity relationship models guide hit-to-lead and lead optimization programmes, aiming to improve the pharmacological activity and physicochemical properties of drug candidates^2–4^. For structure–activity relationship exploration, time-efficient synthesis is important because synthesis represents a bottleneck of the design–make–test–analyse cycle^5^. A number of synthetic methods for the selective activation and modification of C–H bonds allow for the late-stage functionalization (LSF) of organic scaffolds, ranging from molecular building blocks to advanced drug molecules^6^. Numerous catalytic systems offer both, directed and non-directed methods, as well as chemo- and site-selective access to modified analogues. LSF methods in medicinal chemistry include fluorination, amination, arylation, methylation, trifluoromethylation, borylation, acylation and oxidation^7^. Among these methods, C–H borylation is considered the most versatile for rapid compound diversification. Organoboron species can be transformed into an array of functional groups and serve as a robust handle for subsequent C–C bond couplings (Fig. 1a), which enables broad structure–activity relationship studies^8–10^.

However, only a few applications of LSF in drug discovery have been reported to date^11,12^. Most of these rare examples focus on a single LSF reaction type^13–15^. Multiple functional groups and various types of C–H bonds with different bond strengths, electronic properties and steric and functional group environments pose challenges for straightforward LSF; thus, generalizing guidelines for reactivity and selectivity predictions should be applied with caution^11^. Consequently, running a successful LSF campaign often requires time-consuming and resource-intensive experimentation, which is not compatible with the tight timelines and limited assets of many medicinal chemistry projects.

High-throughput experimentation (HTE) is an established approach for reaction optimization^16–18^, enabling semi-automated miniaturized low-volume screenings to rapidly and reproducibly perform multiple transformations in parallel with small amounts of precious building blocks and consumables^19–21^. In combination with FAIR (Findability, Accessibility, Interoperability, Reusability)^22^ documentation, which generates high-quality datasets on successful and failed reactions^23,24^, HTE provides a foundation to unlock LSF for drug discovery by enabling advanced data analysis and machine learning.

Graph neural networks (GNNs) have seen broad applications in molecular feature extraction and property prediction^25–28^. Among the various machine learning methods developed for chemical reaction planning^23,29,30^, GNNs have been successfully employed for retrosynthesis planning, regioselectivity prediction and reaction product prediction^31–34^. In addition, transformers and fingerprint-based methods were developed to tackle similar problems^35,36^. Other studies have shown that learning the activation energies of transition-state geometries yields accurate predictions for competing reaction outcomes^37–39^. Graph featurization with density functional theory (DFT)-level atomic partial charges improved the prediction of regioselectivity for reactions driven by electronic effects^40^. The combination of graph machine learning with HTE enabled the optimization of reaction conditions for the C–H activation of organic substrates^41^. Recently, a GNN-based approach for predicting late-stage alkylation opportunities has been published, mainly focusing on Baran-type diversinate chemistry using alkyl sodium sulfinate salts^42^. Several studies have focused on deep learning models using transition states with the capability of predicting reaction outcomes, including, in some cases, enantioselectivity^43–45^. However, these approaches are limited to small molecular structures and comparably small datasets, rendering the application of such models to structurally more intricate drug-like molecules challenging^46^. A recent study has shown that hybrid machine learning models augmented with the quantum chemical information of transition states enable regioselectivity predictions for iridium-catalysed borylation reactions^47^. Importantly, the influence of steric and electronic effects on the model performance for C–H activation reactions and their application to regioselectivity for molecules with multiple aromatic ring systems remains unexplored.

Here we introduce a geometric deep learning approach applied to automated LSF borylation screening for identifying late-stage hits and lead diversification opportunities (Fig. 1b). Computational deep learning was employed for predicting reaction outcomes, yields and regioselectivity for the LSF of complex drug molecules. In the first step of this study, a comprehensive analysis of the published literature was performed to provide a rationale for selecting suitable reaction conditions for HTE screening and relevant substrates reflecting the nature of late-stage lead compounds in drug discovery. Reaction conditions were chosen from manually curated literature data based on 38 selected publications (the literature dataset). LSF substrates were chosen based on a cluster analysis of 1,174 approved drugs, resulting in 23 structurally diverse drug molecules. This approach enabled us to work with relevant examples of reaction conditions and substrates in an ‘informer library’ approach (that is, an approach involving a chemical space tailored to the assessment of a synthetic methodology) rather than using idealized substrates and fragments with limited applicability to lead optimization^48^. In the second step of the study, semi-automated HTE was used for data generation (the experimental dataset). The reaction data for the selected drug molecules and reaction conditions provided high-quality data for subsequent machine learning of the reaction outcomes. Finally, different GNNs were trained on two-dimensional (2D), three-dimensional (3D) and atomic-partial-charge-augmented molecular graphs, to predict binary (yes/no) reaction outcomes, reaction yields and regioselectivity.

## Results

### High-throughput experimentation

**Fig. 2:**
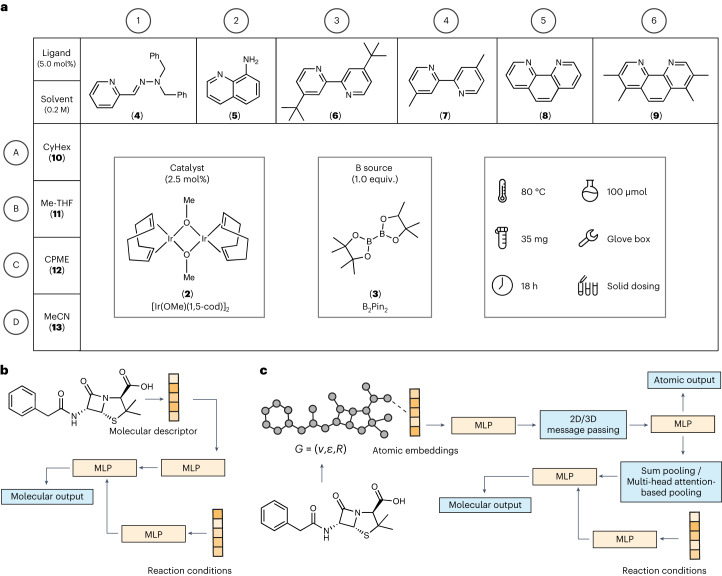
Screening plate overview and GNN architecture. **a**, Schematic of the 24-well borylation screening plates (columns: 1–6, rows: A–D) that were used in the experiments. One catalyst (**2**), one boron source (**3**), six ligands (**4**–**9**) and four solvents (**10**–**13**) were screened for all starting materials. B_2_Pin_2_, bis(pinacolato)diboron; CyHex, Cyclohexane; [Ir(COD)OMe]_2_, (1,5-cyclooctadiene)(methoxy)iridium(I) dimer. **b**, Baseline model composed of a feed-forward neural network, using the molecular descriptor ECFP4 and the reaction conditions as input. Multilayer perceptron (MLP) modules are highlighted in orange, and the output is in blue. This baseline model was applied for the prediction of reaction yield and binary reaction outcomes. **c**, The molecular graph is featured with 2D or 3D information, with or without atomic partial charges (Methods for details on atom featurization). After passing the atomic features through a first MLP, the atomic features are updated via three 2D or 3D message-passing layers. Subsequently, the learned atomic features are either transformed directly to the regioselectivity output, or pooled via sum pooling or multi-head attention-based pooling operations to obtain a whole-molecule feature space. This learned molecular feature space is then combined with the embedded features of the reaction conditions (Methods for details on condition featurization) and transformed to the reaction output (reaction yield, binary reaction outcome) via a final MLP.

Using a HTE set-up and liquid chromatography–mass spectrometry (LCMS) coupled to a reaction data analysis pipeline, 23 drug compounds (**1**, **14**, **16**–**36**; structures of all compounds are in the Supplementary Information (Supplementary Section 3 and Supplementary Figs. 3 and 4)) and 12 drug-like fragments (**37**–**48**; Supplementary Section 3 and Supplementary Fig. 5) were screened using the plate layout depicted in Fig. 2a. Herein, the ensemble of the selected 23 drug compounds and 12 drug-like fragments is referred to as the LSF informer library. The 24-well borylation screening plate was designed based on a comprehensive literature assessment that delivered 1,301 reactions for meta-analysis. A detailed description of this approach is provided in the Methods.

In addition to the LSF informer library, a small subset of five frequently occurring literature substrates (**49**–**53**; Supplementary Section 3 and Supplementary Fig. 5) was screened by applying the borylation conditions. In total, a dataset containing the conditions and results of 956 reactions was obtained. LCMS measurement, followed by data analysis, enabled the determination of (1) binary (yes/no) reaction outcomes, that is, whether the conditions in combination with the individual substrates resulted in the desired mono- or di-borylated products, as well as (2) reaction yields, providing information about the amount of the desired reaction product. A protocol for visualizing the reaction outcome was implemented in the data analysis pipeline, which expedited the identification of starting points for suitable scaled-up procedures. Running selected reactions on larger scales indicated that individual conditions from the miniaturized HTE screenings can be adapted to produce sufficient material for biological tests or further post-borylation modification. In addition, the scale-up reactions enabled the determination of isolated yields and elucidation of the exact structure by nuclear magnetic resonance (NMR) spectroscopy and high-resolution mass spectrometry (HRMS) of a set of selected compounds (**1**, **25**, **29**, **37**–**39** and **45**). These analyses generated a high-quality experimental dataset containing information on the binary reaction outcomes, reaction yields and regioselectivity, which served as the basis for the geometric deep learning platform.

### Geometric deep learning

The geometric deep learning platform introduced in this study consists of a set of different GNNs tailored to learn three targets: binary reaction outcome, reaction yield and regioselectivity. Three different model architectures were investigated, and four different molecular graph representations were evaluated for each architecture (Fig. 2c).

- Architectures. For the reaction tasks (binary reaction outcome, reaction yield), two network architectures were investigated: a GNN using sum pooling and a graph transformer neural network (GTNN) using graph multiset transformer-based pooling^49^. For regioselectivity, an atomistic GNN (aGNN), which learns directly from atomic features, was employed.
- Molecular graphs. To quantify the influence of steric (3D) and electronic (quantum mechanical (QM)) effects, the input molecular graph was featured using 3D- and QM-augmented information, resulting in four different molecular graphs per neural network: 2D, 3D, 2DQM and 3DQM.

The various combinations resulted in eight different GNNs for each of the reaction tasks (binary reaction outcome and reaction yield) and four for regioselectivity (Table 1). For the reaction tasks, a baseline neural network was investigated using the well-established extended connectivity fingerprint (ECFP (ref. ^50^); Fig. 2b).

**Table 1:** Model performance of the GNNs

	Reaction yield *r* value	Reaction yield m.a.e. (%)	Binary reaction outcome (random split), AUC (%)	Binary reaction outcome (substrate split), AUC (%)
**GTNN2D**	0.896 ± 0.006	4.53 ± 0.09	**91.8** **±** **2.1**	52 ± 2
**GNN2D**	0.866 ± 0.005	5.61 ± 0.06	87.5 ± 1.0	51 ± 2
**GTNN3D**	0.884 ± 0.01	4.51 ± 0.11	91.4 ± 0.7	58 ± 4
**GNN3D**	0.877 ± 0.001	5.33 ± 0.34	89.4 ± 0.8	65 ± 5
**GTNN2DQM**	**0.898** **±** **0.003**	4.41 ± 0.17	90.9 ± 1.5	53 ± 5
**GNN2DQM**	0.876 ± 0.01	5.41 ± 0.10	89.0 ± 1.1	59 ± 5
**GTNN3DQM**	0.890 ± 0.01	**4.23** **±** **0.08**	**91.8** **±** **0.9**	**67** **±** **2**
**GNN3DQM**	0.890 ± 0.006	4.88 ± 0.24	89.1 ± 0.9	64 ± 4
**ECFP4NN**	0.885 ± 0.0006	4.55 ± 0.14	89.3 ± 1.3	52 ± 3
	*F*-score (%)	PVV (%)	TPR (%)	Accuracy (%)
**aGNN2D**	38 ± 5	56 ± 1	30 ± 6	88 ± 1
**aGNN2DQM**	39 ± 2	54 ± 2	30 ± 3	88 ± 0.3
**aGNN3D**	59 ± 3	**62** ± **2**	56 ± 4	**90** **±** **1**
**aGNN3DQM**	**60** **±** **4**	**62** **±** **2**	**59** **±** **6**	**90** **±** **1**

The top of the table shows the model performance of the nine investigated neural networks, predicting binary reaction outcomes and reaction yields. Pearson correlation coefficient (*r*) and m.a.e. values were used to quantify reaction yield predictions. Balanced accuracy (AUC) was used to quantify binary reaction outcome predictions. The bottom of the table shows the model performance of the four different aGNNs for regioselectivity prediction in terms of *F*-score, PPV, TPR and accuracy. The numbers represent mean and standard deviation for *N* = 3 independent neural network runs. The numbers in bold indicate the best performance for each of the individual metrics.

### Reaction yield and reaction outcome

Eight different GNNs and the baseline method, ECFP4NN, were optimized to predict reaction yields and binary reaction outcomes.

**Fig. 3:**
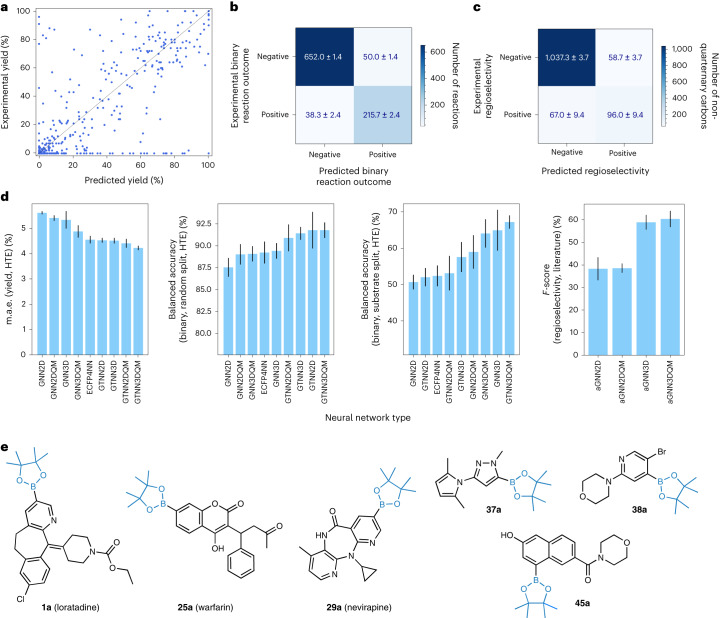
Results of binary reaction outcome, reaction yield and regioselectivity predictions. **a**, Performance of reaction yield prediction on the experimental dataset. The scatter plot shows predicted reaction yields on the *x* axis and experimental reaction yields on the *y* axis for GTNN3DQM. Predictions were obtained from fourfold nested cross-validation, enabling the visualization of the whole dataset (details on dataset splitting are in Supplementary Section 1). **b**, Confusion matrix for binary reaction outcome prediction with a threshold of ≥1% (confusion matrices with additional thresholds are in Supplementary Section 9.3). **c**, Confusion matrix for the prediction of non-quaternary carbons in the test set for aGNN3DQM. **d**, Performance of the investigated neural networks for four different tasks. Each bar plot shows the worst-performing model on the left and the best on the right. Error bars on all bar plots show the standard deviation observed on a threefold cross-validation of independent neural network training runs on the same dataset split. The centre of the error bars denotes the mean performance observed for the threefold cross-validation. The number of predicted reaction data points in the test set (*n*) is annotated individually. The tasks are the m.a.e. as a percent for reaction yield prediction (left; experimental dataset, *n* = 239); balanced accuracy (centre left; AUC) as a percent on the binary reaction outcome prediction using the random dataset split (experimental dataset, *n* = 239); AUC as a percent on the binary reaction outcome prediction using the substrate-based dataset split (centre right; experimental dataset, *n* = 239); and the performance of the four aGNNs for regioselectivity prediction measured in terms of *F*-score (right; literature dataset, *n* = 164). **e**, Selected examples of validated borylation opportunities as predicted by the best-performing neural network (GTNN3DQM) binary reaction outcomes of unseen substrates for three drugs (**1**, **25**, **29**) and three fragments (**37**, **38**, **45**).

The performance of the reaction yield predictions was investigated on a randomly split dataset to learn reaction yields for known substrates in combination with new conditions for the experimental dataset. Figure 3a shows a scatter plot of the predictions of the best-performing neural network, GTNN3DQM, achieving a mean absolute error (m.a.e.) of 4.23 ± 0.08% and a Pearson correlation, *r*, of 0.890 ± 0.01. Figure 3d (left) shows a comparison of the nine different neural networks for this task. The four GTNNs (4.23–4.53% m.a.e.) achieved considerably higher accuracy than the ECFP4NN baseline (4.55% m.a.e.) and the four GNNs (4.88–5.61% m.a.e.). For reaction yield prediction, atomic charges as well as 3D information did not influence the performance of either the GTNNs or GNNs. GTNN models trained on the literature dataset achieve substantially higher errors with m.a.e. values of 16.15–16.73% and a correlation between *r* = 0.59 and *r* = 0.62 (Supplementary Section 9.2 for details). The observation of lower errors for reaction yield predictions for HTE data compared to literature data is in line with recent findings^51^.

Binary reaction outcomes were considered ‘successful’ if the reaction condition with the chosen substrate yielded a mono- or di-borylation product that could be confirmed by LCMS with a corresponding conversion of ≥1%, or ‘unsuccessful’ if the desired transformation was not traceable with LCMS. For the machine learning models trained on binary reaction outcomes, two different dataset splits were investigated: (1) a random split to investigate the performance on new conditions for known substrates; and (2) a substrate-based split for the 23 drug molecules to investigate the performance on unknown substrates with different conditions. First, the binary reaction outcome prediction was evaluated for random data splits (that is, predicting reaction outcomes for novel reaction conditions on known substrates). Figure 3d (centre left) shows a comparison of the nine different neural networks developed for this task. For the binary reaction outcome as observed for reaction yield prediction, a similar trend can be perceived; that is, GTNNs slightly outperformed (90.9–91.8% area under receiver operating characteristic curve, AUC) the ECFP4NN model (89.3% AUC) and GNN model (87.5–89.1% AUC), and the augmentation with atomic partial charges as well as 3D information did not affect the performance of the models (Table 1). Figure 3b shows a confusion matrix that is observed for predictions with a binary threshold of ≥1%. Models with additional binary thresholds of >5%, >10% and >20% were developed (Supplementary Section 9.3), achieving similar accuracy (AUC for 1% threshold, 94.5 ± 0.2%; 5% threshold, 94.5 ± 0.2%; 10% threshold, 95.6 ± 0.3%; and 20% threshold, 94.4 ± 0.2%).

Furthermore, the binary reaction outcome prediction was evaluated for substrate-based data splits (that is, predicting reaction outcomes for novel substrates). For 20 of the 23 unseen drugs, GTNN3DQM achieved an accuracy greater than 50%; for 16 of the 23 unseen drugs, an accuracy greater than 80% was obtained. Overall, the GTNN3DQM model exhibited an AUC value of 67 ± 2% (Table 1). Figure 3d (centre right) shows a comparison of the nine different neural networks for this task, indicating a better performance for the GNNs trained on 3D graphs (58–67% AUC) in comparison to the ECFP4NN (52% AUC) and the GNNs and GTNNs trained on 2D graphs (51–59% AUC). Furthermore, augmentation with atomic partial charges did not show improvements for GNNs or GTNNs. Figure 3e shows three drugs (**1**, **25**, **29**) and three fragments (**37**, **38**, **45**) that were predicted by GTNN3DQM to yield successful reaction outcomes for unseen substrates. The main reaction products of these six substrates were isolated with reaction yields ranging from 5% to 90% (Supplementary Section 11 for experimental details).

### Regioselectivity

Four different aGNN models were developed for regioselectivity prediction by training the neural networks computed for all non-quaternary carbons in a given molecule to determine whether the reaction will occur. As borylation reactions regularly occur at one atom or, in rare cases, at two atoms in a molecule, the atomic labels ‘reactive’ and ‘non-reactive’ in a molecule are unbalanced (approximately 1:6). Therefore, the *F*-score (that is, the mean of positive predictive value (PPV) and true positive rate (TPR)) was used as a measure of neural network accuracy.

**Fig. 4:**
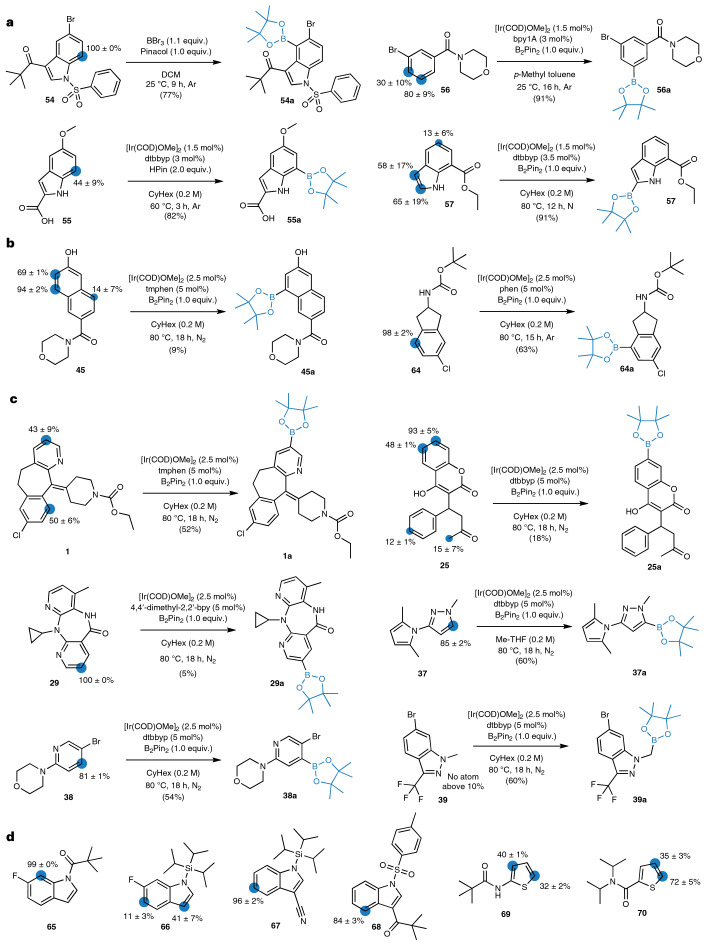
Selected examples from the borylation regioselectivity prediction. **a**–**d**, For each transformation, the predicted regioselectivity is shown on the left, and borylation including the reported reaction conditions and experimentally validated regioselectivity are shown on the right. The percentages for the regioselectivity predictions were generated by aGNN3D through the mean and standard deviation on ten individual conformers. Every prediction resulted in a value between zero and one, where one was set to 100%. **a**, Retrospective results obtained from the test set of the literature dataset. Results for two reactions from the top 20% (**54**, **55**) and bottom 20% (**56**, **57**) of the predictions from the literature dataset. **b**, Retrospective results obtained from out-of-distribution reactions from Roche legacy projects. Validation is shown for two molecules (**45**, **64**). **c**, Prospective experimental validation of regioselectivity prediction models that were trained on the literature dataset. Validation is shown for three drugs, Loratadine (**1**), warfarin (**25**) and nevirapine (**29**), and three fragments, **37**, **38** and **39**. **d**, Influence of steric hindrance and directing functional groups on regioselectivity prediction for six selected examples from the test set of the literature dataset. Regioselectivity predictions of indole derivatives (**65**–**68**) and thiophene derivatives (**69**, **70**). The numbering of the shown indole molecule starts with 1 for the nitrogen atom and proceeds around the carbons in the ring, numbering the carbon atoms 2–7. DCM, dichloromethane; BBr_3_, boron tribromide; dtbbpy, 4,4′-di-tert-butyl-2,2′-dipyridyl; byp1A, 1-(2-([2,2′-bipyridin]-5-yl)phenyl)-3-cyclohexylurea; byp, bi-pyridine; Cy, cyclohexane; HPin, pinacolborane; [Ir(COD)OMe]_2_, (1,5-cyclooctadiene)(methoxy)iridium(I) dimer; phen, 1,10-phenanthroline; tmphen, 3,4,7,8-tetramethyl-1,10-phenanthroline; N_2_, nitrogen.

Figure 3d (right) shows the performance of four aGNNs trained on the literature dataset. The aGNNs trained on 3D graph structures outperformed those trained on 2D graph structures (Table 1 shows the exact numbers). The graph structures that included atomic partial charges did not appear to improve the prediction accuracy of the networks compared to their 2D and 3D equivalents. The aGNN3DQM model was the best-performing model overall, with an *F*-score of 60 ± 4%. Figure 4c shows six selected predictions of the test set using aGNN3DQM; on the left side, three reactions from the top 20% are shown, and on the right side, three molecules from the bottom 20% of the test set are shown. Figure 3c features the confusion matrix of the aGNN3DQM predictions on the test set. For the 1,259 non-quaternary carbons in the test set, aGNN3DQM achieved an accuracy of 90 ± 1%, a PPV of 62 ± 2% and a TPR of 59 ± 6%. Table 1 lists the accuracy, PVV values, TPR values and *F*-scores of the four aGNN models. The aGNNs trained on 2D graph structures yielded a similar false positive rate (that is, similar PPV), but a much higher false negative rate (that is, lower TPR) than the aGNNs trained on 3D graph structures.

The regioselectivity prediction method aGNN3D was trained and subsequently validated on the literature dataset. Test set predictions revealed many accurate examples (Fig. 4a; **54**, **55**) but also pointed to certain limitations of the computational model (Fig. 4a; **56**, **57**). For additional testing, aGNN3D was retrospectively applied to out-of-distribution reactions containing substrates outside of the literature dataset found in Roche Medicinal Chemistry legacy projects (Fig. 4b). The model predicted three potential sites of reaction for morpholine **45**, two of which were experimentally confirmed. For carbamate **64**, the correct site of borylation and one false positive site were predicted. The aGNN3D model was then prospectively validated using six selected borylation reactions of the drugs Loratadine (**1**), warfarin (**25**) and nevirapine (**29**), and three fragments (**37**, **38**, **39**; Fig. 4c).

The prediction model achieved approximately 70% accuracy in this experiment. Five of seven experimentally observed borylation sites were correctly predicted by the model. Figure 4c illustrates the six predictions compared to the isolated and characterized products obtained through the scaled-up reactions of the best-observed screening conditions. For fragments **37** and **38** and the drug nevirapine (**29**), the model predicted only one site of borylation. The predicted sites were experimentally confirmed, and neither false positive nor false negative predictions were observed. For Loratadine (**1**), aGNN3D predicted two potential reaction sites. The predicted mono-borylation product **1a** was isolated, and the regioselectivity prediction was confirmed. For the second predicted species, the exact position of the two pinacol esters on Loratadine (**1**) could not be directly confirmed by NMR, but the respective mass was confirmed by HRMS. Product **1b** was consequently subjected to hydrolysis to obtain the corresponding phenol **1c** (Supplementary Section 11). The analysis revealed that the second prediction was incorrect. For warfarin (**25**), aGNN3D predicted two potential reaction sites, scoring 93 ± 5% and 48 ± 1%. Mono-borylation of the C–H bond with the most confident prediction (93%) was experimentally confirmed. For fragment **39** the regioselectivity model did not suggest that borylation occurs, but mono-borylation was observed during the screening, and a scale-up was conducted. This analysis revealed that **39** in fact underwent mono-borylation of the methyl group to deliver **39a**.

Finally, we investigated the influence of substitutions with different steric hindrances and electronic effects on the regioselectivity predictions. The aGNN3D model was applied to six unseen examples from the literature test set that introduce steric hindrance or directing functional groups. Figure 4d illustrates the regioselectivity predictions for four indole derivatives. Placing a directing amide functionality in position 1 yielded a prediction of 99 ± 0% at position 7 (Fig. 4d). Substituting the directing amide functionality with a bulky triisopropylsilane blocks position 7 and therefore yielded a score of 41 ± 7% for position 3 (Fig. 4d). Furthermore, blocking position 3 with a cyano group and keeping the triisopropylsilane in position 1 in place yielded a prediction score of 96 ± 2% for position 5 (Fig. 4d). For a directing keto functionality at position 3, a score of 84 ± 3% was obtained for position 4 (Fig. 4d, right). Figure 4d illustrates the regioselectivity predictions for two thiophene derivatives. Placing a directing secondary amide functionality at position 2 shows a slight preference at position 3 with a score value of 40 ± 1% (Fig. 4d). Replacing the directing secondary amide at position 2 with a bulky tertiary amide shifts the high score (72 ± 5%) to position 5 (Fig. 4d). For all of these examples, the highest prediction is in line with observed mono-borylations in the literature^52–55^. These results conclude that the regioselectivity prediction model aGNN3D successfully considers steric and electronic substituent effects.

## Discussion

Curated high-quality reaction data are key drivers of successful deep learning. The results of this study were obtained using two FAIR datasets (that is, literature and experimental) containing 1,301 and 956 reactions, respectively. To lower the barrier to sharing reaction data, we developed a comprehensive reaction data format (SURF, simple user-friendly reaction format) that allows for FAIR data capture. A detailed description of the SURF structure and data templates is provided in Supplementary Section 7. SURF complements similar initiatives, such as the open reaction database (ORD) and unified data model (UDM)^56,57^. It was developed to enable scientists to store and share reaction data in an easily editable format. High-quality literature data and newly generated experimental reaction data have enabled in silico estimation of reaction outcomes and reaction selectivity. The resulting geometric deep learning platform has been shown to correctly predict the reaction outcome for six substrates, and their main products were isolated (Supplementary Section 11). This approach represents a tool for identifying late-stage modifications of advanced drug-like molecules before initiating resource-intensive synthesis.

Two GNN architectures were implemented to predict the reaction tasks (binary reaction output and reaction yield). The two models, GNN and GTNN, differ only in their pooling operations. Whereas the GNN uses sum pooling, the GTNN relies on more complex graph multiset transformer-based pooling. This additional flexibility of the GTNNs slightly improved the reaction yield prediction but did not lead to increased prediction performance for binary reaction outcomes. This result suggests that greater neural network flexibility may lead to improved prediction accuracy for certain reaction prediction tasks but does not offer a general advantage.

The best-performing neural network model for reaction yield prediction (GTNN3DQM) achieved a m.a.e. of 4.23 ± 0.08% with a Pearson correlation of *r* = 0.890 ± 0.01 on the experimental dataset (Table 1), whereas the most accurate model for literature data prediction (GTNN2DQM) achieved a m.a.e. of 16.11 ± 0.02% with *r* = 0.61 ± 0.01 (Supplementary Section 9.2 for details). This disparity can be explained by the heterogeneity and quality of the two datasets. The experimental data were generated in the same laboratory using the same equipment for syntheses and analyses and included the same standard for determining the reaction yield in all experiments. Furthermore, the experimental dataset covers a less diverse reaction parameter space (that is, 24 versus 864 possible conditions per substrate), thereby facilitating the learning task. By contrast, the reaction outcomes in the literature dataset originate from a variety of experiments performed in different laboratories that used different methods for determining the yield (for example, isolated yield, reaction conversion assessed by NMR, LCMS). Standardized, chemically diverse, high-quality datasets will be beneficial for building accurate machine learning models that enable further optimization of reaction conditions for LSF.

Importantly, the incorporation of steric information via 3D molecular graphs led to improved neural network performance for all investigated tasks, ranging from small enhancements in reaction yield prediction (m.a.e., 4.2% versus 4.4%) and binary reaction outcomes (AUC, 67% versus 59%) to substantial improvements in regioselectivity predictions (*F*-score, 60% versus 39%). Implementing partial charges generated with DFT accuracy into neural networks did not exhibit any improvements in all investigated tasks. However, the explored borylation reactions are mainly guided by steric effects and, to a lesser extent, electronic effects^58,59^, which could explain these observed effects. Incorporating the local 3D geometry considerably improved regioselectivity predictions from 38 ± 5 for the best-performing 2D model to 60 ± 4% for the best-performing 3D model. These observations demonstrate the relevance of the local geometries and the additional information provided by 3D graphs for reactivity prediction on the level of individual atomic environments.

Regioselectivity predictions on the literature data delivered accurate results for the majority (90%) of the cases. The four selected and validated substrates from the experimental dataset highlight the reaction biases in the literature data used for model training. Specifically, the majority of the borylations captured in the literature dataset occur at *sp*^2^ carbons on substrates with no more than two ring systems. Substrates that fulfil these characteristics, such as fragments **37** and **38**, are predicted correctly. However, substrates outside of this scope, including the *sp*^3^-carbon borylation on fragment **39** or the di-borylation on the annulated pentadecanyl moiety in Loratadine (**1**), exploit the limitations of the available literature data. These results conclude that small datasets, such as the presented 1,301 reactions from the literature in this study, are sufficient for predicting regioselectivity with GTNNs on substrates similar to the ones covered by the chemical space in the literature. However, to predict regioselectivity in a trustworthy manner for a broader chemical space including larger molecules and potentially also *sp*^3^ borylations, further training data will be required.

The LSF informer library containing 23 structurally diverse, approved drugs (**1**, **14**–**36**) complemented with 12 fragments (**37**–**48**) and five idealized substrates (**49**–**53**) yielded a dataset covering the essential chemical motifs relevant in drug discovery. A functional group analysis revealed that 33 (82.5%) of the 40 most abundant functional groups extracted from the 1,174 drug molecules are covered by the LSF informer library. Further analysis highlighted that functional groups that are known to exhibit the desired borylation reaction, such as aromatic nitrogens, aromatic alkyl-oxy groups and alcohols, are also among the functional groups in the LSF informer library that show the highest tolerance for successful reaction outcomes. On the contrary, certain functional groups such as primary amines, carbamates and carbonates, or aromatic functional groups with strong electron-withdrawing moieties (for example, nitro-aryls) were found to be less tolerated and inhibit desired reaction outcomes (Supplementary Section 8.2 for further details on the functional group analysis). Since every substrate was screened with every reaction condition, further insights about reaction conditions could be gained (Supplementary Tables 4 and 5). Whereas the best-performing ligand was **9** (33%), **6**–**8** (28–30%) showed similar good results, whereas **5** (22%) and especially **4** (17%) delivered fewer successful reaction outcomes. Moreover, reaction outcomes were further influenced by solvents. Cyclohexane (**10**, 50%) outperformed the other three solvents 2-methyltetrahydrofuran (Me-THF; **11**, 43%), cyclopentyl methyl ether (CPME; **12**, 38%) and acetonitrile (MeCN; **13**, 29%).

HTE and GNNs have previously been used for identifying substrates suitable for C–H activation^41^. This present study extends this original approach by (1) using HTE and GNNs for drug molecules, (2) introducing a literature search strategy that enables the selection of a structurally diverse set of substrates and ideal plate reaction screening conditions and (3) introducing a flexible geometric deep learning approach that considers the influence of steric and electronic effects of the substrates and allows the prediction of reaction outcome, yield and regioselectivity.

The structural and shape diversity of the compounds used for training the regioselectivity prediction model considerably exceeds the compound diversity of a recent report on regioselectivity prediction for iridium-catalysed borylation reactions^47^. Compound clustering, scaffold and shape analyses of both datasets revealed greater chemical diversity of our training data. Furthermore, the neural networks were developed with more examples and broader chemical space coverage (Supplementary Section 9.1, Supplementary Figs. 14 and 15 and Supplementary Tables 6 and 7). Importantly, the estimated three dimensionality of the data is characteristic of molecules typically observed in medicinal chemistry^60^. These findings positively advocate for using these computational models for drug discovery.

In conclusion, the results of this study confirm the practical applicability of the geometric deep learning platform in bioorganic and medicinal chemistry and their potential benefit for laboratory automation. The approach is routinely and successfully applied to assess binary reaction outcome, reaction yield and regioselectivity for borylation opportunities in drug discovery projects at F. Hoffmann-La Roche Ltd. Additional data points are continuously generated by standardized HTE to further enhance the predictive power of the computational models presented. For future improvements, (1) additional reaction conditions for iridium-catalysed borylation will be explored. This extended screening panel could include exchanging the catalyst or boron source as well as using a broader variety of ligands and solvents. In addition, (2) the LSF informer library can be augmented to include more frequently occurring fragments in drug molecules to expand the relevant chemical space and potentially improve the performance of the machine learning pipeline. Finally, (3) less frequently employed transition-metal-catalysed or even metal-free synthesis methods can be investigated to enhance the coverage of the reaction conditions, addressing reactions from publications initially excluded from the analysis.

## Methods

### Literature analysis

The systematic analysis of chemical transformations (SACT) of the data retrieved from literature consisted of four steps: (1) literature search, (2) literature data curation and evaluation, (3) methodology extraction and (4) reaction data curation and analysis. All details of the literature analysis are provided in Supplementary Section 2. The literature analysis identified 38 publications describing relevant borylation methods, from which the reaction data were manually extracted to obtain a high-quality dataset containing 1,301 chemical transformations. Meta-analysis of these data provided a foundation for an informed plate design.

### LSF informer library

The concept of chemical informer libraries, initially reported by Merck^48,61^, served as the basis for developing the LSF informer library. Applying a clustering method based on structural features to a dataset containing 1,174 approved small-molecule drugs yielded eight structurally diverse groups of molecules. Details of the applied clustering and visualization of the cluster via principal component analysis are provided in Supplementary Section 3. Three molecules were selected from each cluster based on their distance from the cluster centre, price and availability and were subjected to borylation screening. To complement the model with fragments relevant to Roche’s chemical space, the top 100 most popular ring assemblies found in the Roche corporate compound collection were identified. For these ring assemblies, substructure searches were performed for the entire database. The resulting compounds were retained if (1) the structures had a molecular weight below 300 g mol^−1^ or fewer than 20 non-hydrogen atoms, (2) there was at least 1 g of powder stock available and (3) the structures were not used in any internal project or subject to legal restrictions. Out of this pool of candidates, 12 fragments were manually selected. Further details on the determination and constitution of the LSF informer library are described in Supplementary Section 3.

### Screening plate design

Following the SACT approach that delivered a curated high-quality literature data set, a meta-analysis was conducted to define a clear rationale for determining the conditions for the 24-well borylation screening plate used for the LSF informer library. This analysis included the temperature (*T*), time (*t*), reaction concentration (*c*) and scale (*n*), selected based on the median values for our screening plate (*T* = 80 °C, *t* = 16 h, *c* = 0.2 M, *n* = 100 mmol). Subsequently, the number of reaction components generally used for borylation reactions (catalyst, ligand, boron source and solvent) was determined. Owing to the limited space on the 24-well plate and the high occurrence of [Ir(COD)(OMe)]_2_ (**2**), **2** was chosen as the catalyst. Analysis of the reagents used in combination with **2** provided the rationale for choosing B_2_Pin_2_ (**3**) as the boron source. This selection made it possible to screen a set of six ligands and four solvents. Six rather than four ligands were used because the dataset showed a greater variety of ligands than solvents. The ligands were assessed based on the chemical diversity of the converted starting materials and their commercial availability. Based on these results, six ligands from four chemical classes were selected. While the meta-analysis revealed that low-boiling solvents are the predominant solvents for borylation, their corresponding higher-boiling analogues (for example, Me-THF instead of tetrahydrofuran, THF) were selected to avoid potential solvent evaporation at 80 °C and reduce the risk of cross-contamination. The detailed meta-analysis results leading to the final plate design are described in Supplementary Section 4.

### HTE borylation screening

Using a 24-well plate design (Fig. 3), all drug molecules from the LSF informer library and selected fragments (Supplementary Section 3 and Supplementary Figs. 3–5) were screened. The reaction set-up (automated solid dosing and solvent addition) and execution (heating and stirring) in glass vials on a parallel screening plate were conducted in a glove box under a nitrogen atmosphere. Upon completion of the reaction, the solvents were removed through evaporation, followed by automated resuspension of the residues in MeCN/H_2_O and dilution to a defined concentration for LCMS analysis using a liquid handler. The samples were then analysed by LCMS, and the resultant data were subjected to an automated reaction data analysis pipeline (Supplementary Figure 6) to rapidly determine all components within the mixture. Standardized reaction data output (SURF; Supplementary Section 7) allowed direct visualization of reaction outcome with the TIBCO Spotfire software as well as the direct loading into machine learning models. The general screening procedure, including detailed information on the hardware and software used, is provided in Supplementary Sections 5 and 6).

### Scaled-up reactions

Selected molecules (three drugs, **1**, **25** and **29**; and four fragments, **37**, **38**, **39** and **45**) showing substantial conversion to the respective borylation products were scaled up using the most promising conditions. All reactions were conducted under a nitrogen atmosphere in a glove box using glass reaction vessels with pressure release caps and standard stirring bars. Purification was performed using flash chromatography or reversed-phase high-pressure liquid chromatography. In selected cases, where separation of the borylated species could not be achieved, the boronic ester was transformed into a hydroxyl group. Structural elucidation was performed using NMR and HRMS. The full analytical results and spectra for all compounds are shown in Supplementary Sections 11 and 12.

### Deep learning

#### Graph neural network architecture

The following paragraphs describe the neural network architecture of the three introduced GNNs (that is, GNN, GTNN and aGNN). GNN and GTNN were trained to learn the two reaction properties (that is, binary reaction outcome and reaction yield), and aGNN was trained to learn regioselectivity. Details about dataset splitting are in Supplementary Section 1.

Molecular graph. For each of the three GNNs (that is, GNN, GTNN and aGNN), four different input molecular graph representations were investigated, which include steric (3D) and electronic (QM) features in different combinations, yielding four different molecular graphs: 2D, 2DQM, 3D and 3DQM.

E(3)-invariant message passing. The atomic features and optionally DFT-level partial charges were embedded and transformed using a MLP, resulting in atomic features ${{{{\bf{h}}}}}_{i}^{0}$. E(3)-invariant message passing in a similar fashion as suggested by Satorras et al.^62^ was applied to *l* layers over all atomic representations ${{{{\bf{h}}}}}_{i}^{0}$ and their edges. Edges were defined by covalent bonds for the 2D graph and all atoms within a radius of 4 Å for the 3D graph, respectively. All networks contained three message-passing layers. In each message-passing layer, the atomic representations were transformed via equation (1)

1 $${{{{\bf{h}}}}}_{i}^{l+1}=\phi \left({{{{\bf{h}}}}}_{i}^{l},\mathop{\sum}\limits_{j\in {{{\mathcal{N}}}}(i)}\psi \left({{{{\bf{h}}}}}_{i}^{l},{{{{\bf{h}}}}}_{j}^{l}\right)\right),$$

for 2D graph structures, and equation (2)

2 $${{{{\bf{h}}}}}_{i}^{l+1}=\phi \left({{{{\bf{h}}}}}_{i}^{l},\mathop{\sum}\limits_{j\in {{{\mathcal{N}}}}(i)}\psi \left({{{{\bf{h}}}}}_{i}^{l},{{{{\bf{h}}}}}_{j}^{l},{{{{\bf{r}}}}}_{i,j},\right)\right),$$

for 3D graph structures.

In equations (1) and (2), ${{{{\bf{h}}}}}_{i}^{l}$ is the atomic representation **h** of the *i*th atom at the *l*th layer; $j\in {{{\mathcal{N}}}}(i)$ is the set of neighbouring nodes connected via edges; **r**_*i*,*j*_ the interatomic distance features (Methods, “Atom featurization” for details); *ψ* is a MLP transforming node features into massage features **m**_*ij*_ as ${{{{\bf{m}}}}}_{ij}=\psi ({{{{\bf{h}}}}}_{i}^{l},{{{{\bf{h}}}}}_{j}^{l},{{{{\bf{r}}}}}_{i,j})$ for 3D graphs and ${{{{\bf{m}}}}}_{ij}=\psi ({{{{\bf{h}}}}}_{i}^{l},{{{{\bf{h}}}}}_{j}^{l})$ for 2D graphs; ∑ denotes the permutation-invariant pooling operator (that is, sum) transforming **m**_*ij*_ into **m**_*i*_ as ${{{{\bf{m}}}}}_{i}={\sum }_{j\in {{{\mathcal{N}}}}(i)}{{{{\bf{m}}}}}_{ij}$; and *ϕ* is a MLP transforming ${{{{\bf{h}}}}}_{i}^{l}$ and **m**_*i*_ into ${{{{\bf{h}}}}}_{i}^{l+1}$. The atomic features from all layers $[{{{{\bf{h}}}}}_{i}^{l = 1},{{{{\bf{h}}}}}_{i}^{l = 2},{{{{\bf{h}}}}}_{i}^{l = 3}]$ were concatenated and transformed via a MLP, resulting in final atomic features **H**. **H** was then transformed differently by the three GNNs, using sum pooling (GNN) or multi-head attention-based pooling (GTNN) to obtain molecular outputs (that is, reaction yield and binary reaction outcome), or no pooling (aGNN) for regioselectivity prediction.

GNN. Atom features **H** were pooled via sum pooling, transformed via an additional MLP, concatenated to a learned representation of the reaction conditions (Methods, “Condition featurization” for details) and transformed to the desired output via a final MLP.

GTNN. A graph multiset transformer^49^ was incorporated into the GTNN architecture for pooling the atomic features into a molecular feature. The nodes **H** were transformed using the Attn function: Attn(**Q**,**K**,**V**) = **QK**^*T*^**V**, where query **Q**, key **K** and value **V** are learned features from the node representations **H**. **Q** is learned via individual embedding vectors per attention head. **K** and **V** are learned via individual GNNs GNN^*K*^ and GNN^*V*^ resulting in the overall graph attention head via equation (3):

3 $${{{{\bf{o}}}}}_{i}={{{\rm{Attn}}}}({{{\bf{H}}}}{{{{\bf{W}}}}}^{Q},{{{{\rm{GNN}}}}}_{i}^{K}({{{\bf{H}}}},{{{\mathcal{E}}}}),{{{{\rm{GNN}}}}}_{i}^{V}({{{\bf{H}}}},{{{\mathcal{E}}}}))$$

where **o**_*i*_ denotes the weighted pooling vector from one attention head, and **W**^*Q*^ is a linear layer to learn the query vectors from **H**. Herein, four attention heads are incorporated, yielding the pooling scheme graph multi-head attention block GMH: GMH(**Q**,**H**,${{{\mathcal{E}}}}$) = [**o**_1_, **o**_2_, **o**_3_, **o**_4_]**W**^*o*^. This learned molecular representation was transformed via an additional MLP, concatenated to a learned representation of the reaction conditions (Methods, “Condition featurization” for details) and transformed to the desired output via a MLP network.

aGNN. No pooling of atom features was applied, and **H** was directly transformed to the desired atomic output via a final MLP with a sigmoid activation function.

#### Training details

PyTorch Geometric (v.2.0.2)^63^ and PyTorch (v.1.10.1+cu102)^64^ functionalities were used for neural network training. Training was performed on a graphical processing unit, GPU (Nvidia GeForce GTX 1080 Ti) for four hours, using a batch size of 16 samples. The Adam stochastic gradient descent optimizer was employed^65^ with a learning rate of 10^−4^, a mean squared error (m.s.e.) loss on the training set, a decay factor of 0.5 applied after 100 epochs and an exponential smoothing factor of 0.9. Early stopping was applied to the model that achieved the lowest validation m.a.e. within 1,000 epochs. All the models considered in this study were trained on the Euler computing cluster at ETH Zurich, Switzerland.

#### Atom featurization

Atomic properties were encoded via the following atomic one-hot-encoding scheme: twelve atom types (H, C, N, O, F, P, S, Cl, Br, I, Si, Se), two ring types (true, false), two aromaticity types (true, false) and four hybridization types (*sp*^3^, *sp*^2^, *sp*, *s*). Additionally, for molecular graphs that contained electronic features, the atomic partial charges were calculated on the fly using DelFTa software^66–68^, obtaining DFT-level (ωB97X-D/def2-SVP (refs. ^69,70^)) Mulliken partial charges^71^. For molecular graphs that contained 3D information, the interatomic distances were represented in terms of Fourier features, using a sine-based and cosine-based encoding as previously shown in ref. ^66^.

#### Condition featurization

Molecular reaction conditions, that is, solvents, ligands, catalysts and reagents, were one-hot encoded. Whereas, the experimental dataset covered six ligands and four solvent types (that is, 24 possible conditions per substrate), the literature dataset covered twelve ligands, nine solvents, two reagents and four catalyst types (that is, 864 possible conditions per substrate). Supplementary Section 4 gives a detailed description of the structures covered by these one-hot-encodings.

#### Conformer generation

The 3D conformers were calculated using RDKit (AllChem.EmbedMolecule (ref. ^72^)) followed by energy minimization via the universal force field (UFF) method^73^. For each molecule, ten different conformers were calculated for training and testing. A conformer was randomly selected at each training step. For testing, the final predictions were obtained by averaging the individual predictions calculated for each of the ten conformers.

#### Baseline model

The ECFP4NN baseline model combined three MLPs for input transformation, namely the ECFP4 fingerprint and two embedded reaction conditions (that is, solvent and ligand). The ECFP4 feature dimension was set to 256 after screening the feature dimensions in the range of 2^7^−2^10^. Additional baseline experiments using binary reaction fingerprints with two popular decision tree algorithms, gradient boosting and extreme gradient boosting (XGBoost), can be found in Supplementary Section 10.

#### Number of hyperparameters

The feature dimension of the GNN internal representation was set to 128, except for (1) the embedding dimension of the reaction and atomic properties,tr which was set to 64, and (2) the first MLP layer after the graph multiset transformer-based pooling, which was set to 256. This setting resulted in neural network sizes of ~2.0 million trainable parameters for the GNN and aGNN models and ~3.0 million trainable parameters for GTNN. The dimensions within ECFP4NN were maintained at 128 yielding a neural network size of ~2.0 million trainable parameters.

#### Dataset filtering and reaction yield

From the total number of 1,301 reactions in the literature dataset, 492 reactions were used for yield prediction. Two filtering criteria were applied to obtain these training data: (1) duplicate reactions were removed, that is, reactions with identical annotations for starting material, catalyst, solvent, reagent, and product, and (2) only those reactions were included that included catalysts, solvents, reagents, and that occurred at least four times in the whole dataset (in line with the one-hot encoding described in Methods, “Condition featurization”).

#### Dataset filtering and regioselectivity

From the total number of 1,301 reactions in the literature dataset, 656 reactions were used for regioselectivity prediction. Three filtering criteria were applied to obtain these training data: (1) duplicate products (reactions with identical products) were removed, (2) only reactions using B_2_Pin_2_ (that is, bis(pinacolato)diboron) as the borylation product were kept and (3) an annotated yield of ≥30% was required.

## Online content

Any methods, additional references, Nature Portfolio reporting summaries, source data, extended data, supplementary information, acknowledgements, peer review information; details of author contributions and competing interests; and statements of data and code availability are available at 10.1038/s41557-023-01360-5.

### Supplementary information

Supplementary Information Full supplementary information for the main manuscript.

## Data Availability

The SURF-formatted literature and experimental datasets containing 1,301 and 956 reactions, respectively, as well as a SURF template are available at https://github.com/ETHmodlab/lsfml (https://zenodo.org/record/8118845).

## Code availability

A reference implementation of the geometric deep learning platform based on PyTorch^64^ and PyTorch Geometric^63^ is available at https://github.com/ETHmodlab/lsfml (https://zenodo.org/record/8118845).
