# Supplementary material for: Enabling late-stage drug diversification by high-throughput experimentation with geometric deep learning
Source: Nat Chem. 2023 Nov 23;16(2):239–48. doi: 10.1038/s41557-023-01360-5 (PMC10849962; doi:10.1038/s41557-023-01360-5)
Supplement: Supplementary file 1 — Full supplementary information for the main manuscript. [file 41557_2023_1360_MOESM1_ESM.pdf]

nature chemistry

Article

https://doi.org/10.1038/s41557-023-01360-5

Enabling late-stage drug diversification by high-throughput experimentation with geometric deep learning

In the format provided by the authors and unedited

Supplementary Information: Enabling late-stage drug diversification by high-throughput experimentation with geometric deep learning

David F. Nippa[1,2,†], Kenneth Atz[3,†], Remo Hohler[1], Alex T. Müller[1], Andreas Marx[1], Christian Bartelmus[1], Georg Wuitschik[1], Irene Marzuoli[4], Vera Jost[1], Jens Wolfard[1], Martin Binder[1], Antonia F. Stepan[1], David B. Konrad[2,*], Uwe Grether[1,*], Rainer E. Martin[1,*] & Gisbert Schneider[3,5,*]

[1]Roche Pharma Research and Early Development (pRED), Roche Innovation Center Basel, F. Hoffmann-La Roche Ltd., Grenzacherstrasse 124, 4070 Basel, Switzerland.
[2]Department of Pharmacy, Ludwig-Maximilians-Universität München, Butenandtstrasse 5, 81377 Munich, Germany.
[3]ETH Zurich, Department of Chemistry and Applied Biosciences, Vladimir-Prelog-Weg 4, 8093 Zurich, Switzerland.
[4]Process Chemistry and Catalysis (PCC), F. Hoffmann-La Roche Ltd., Grenzacherstrasse 124, 4070 Basel, Switzerland.
[5]ETH Singapore SEC Ltd, 1 CREATE Way, #06-01 CREATE Tower, Singapore, Singapore.
† These authors contributed equally to this work.
∗ To whom correspondence should be addressed.
E-mail: david.konrad@cup.lmu.de, uwe.grether@roche.com, rainer_e.martin@roche.com, gisbert@ethz.ch

Contents

SI1 Data set splitting **2**

SI2 Systematic literature analysis **3**

SI3 LSF informer library **7**

SI4 Screening plate design **11**
SI4.1 Reaction conditions . . . 11
SI4.2 Catalyst . . . 11
SI4.3 Ligand . . . 12
SI4.4 Reagent / boron source . . . 13
SI4.5 Solvents . . . 13
SI4.6 Plate design . . . 13

SI5 HTE borylation screening protocol **14**

SI6 Automated reaction data analysis pipeline **15**

SI7 SURF convention **17**

SI8 Further analysis of the experimental data set **18**
SI8.1 Molecular property distribution . . . 18
SI8.2 Functional group analysis . . . 19

SI9 Further analysis of the literature data set **21**
SI9.1 Diversity analysis for regioselectivity data set . . . 22
SI9.2 Model performance on the literature data set . . . 24
SI9.3 Different thresholds for binary reaction outcome prediction . . . 26

SI10 Decision tree algorithms using reaction fingerprints **27**

SI11 Borylation scale-ups **29**
SI11.1 Reagent and purification information . . . 29
SI11.2 Analytical information . . . 29
SI11.3 Experimental procedures and analytical data . . . 30

SI12 NMR spectra **42**

SI1 Data set splitting

For the three random split tasks (yield-, binary-, and regioselectivity-prediction), the data set was randomly split into training (50%), validation (25%), and test set (25%). For two of the three tasks (yield, and binary reaction outcome prediction), three-fold cross-validation was conducted for each using the same test set, for eight different graph neural networks and one ECFP baseline, within a random split, resulting in 27 training runs for each of the two tasks. The scatter plot in Figure 3a in the main document was created by the best-performing neural network (GTNN3DQM) using a nested four-fold cross-validation with four individual test sets covering the whole data set. The regioselectivity prediction was conducted in a similar manner with the only difference that four graph neural networks and no ECFP-baseline were trained, resulting in 12 training runs. A substrate-based split was additionally conducted for the binary reaction outcome prediction, where all reactions for one substrate were placed into the test set (2.5%), and the remaining data set was randomly split into training set (65%) and validation set (32.5%). For the substrate-based split, three-fold cross-validation was conducted for eight different graph neural networks and one ECFP baseline, for one split per substrate (23), resulting in 621 training runs. See Table S1 for additional details w.r.t. data set splitting.

Table S1: Overview of the neural networks trained for the four different tasks.

**Task**	**Folds** / N	**Networks** / N	**Splits** / N	**Runs** / N	**data set**
**Binary**	3	9	1 (random)	**27**	Experimental
**Yield**	3	9	1 (random)	**27**	Experimental
**Regioselectivity**	3	4	1 (random)	**12**	Literature
**Binary**	3	9	23 (substrate-based)	**621**	Experimental
**Yield**	3	9	1 (random)	**27**	Literature

SI2 Systematic literature analysis

The systematic analysis of chemical transformations (SACT) can be split up into four major steps: (1) literature search, (2) literature data curation and evaluation, (3) methodology extraction, (4) reaction data curation and analysis.

The literature search (1) can be conducted using keyword- or structure-based queries for the desired transformation allowing a comprehensive assessment of the field. For this study, the keyword-based approach was selected, which consisted of four main query categories: Methodology (M), starting material (S), review/article (R) and catalytic system (C). The M category search aimed at identifying different types of borylations (*e.g.* directed, undirected). The S pillar focused on detecting methodologies for various starting materials (*e.g.* aromatic, aliphatic) and included the hybridisation of the reacting C atom (sp^2, sp^3) as well. Category R is centred around the publication type (methodology or review paper). In addition to enclosing the typical borylation catalyst metals (*e.g.* Ir, Rh), metal-free methods were part of the catalytic system (C) search. Tables S2 and S3 showcase all search queries for this research paper. To balance the strengths and weaknesses, *e.g.* the number of records, of scientific databases, [1] the queries were run on three different, renowned tools, Scopus (Elsevier, Amsterdam, Netherlands), Web of Science (Clarivate Analytics, Philadelphia, USA) and SciFinder-n (Chemical Abstracts Service, Columbus, USA), on the 3rd of November 2021.

Table S2: The first four categorized queries that were carried out on SciFinder, Scopus and Web of Science. Sections indicated with (M) are modified for the other queries. Those modifications are shown in Table S3.

**Query**	**Methodology**	**Starting Material**	**Review / Article**	**Catalytic System**
**Query Name**	M1	S2	R1	C1
**Title**	borylation	borylation	borylation	borylation
**Connector**	AND	AND	AND	AND
**Keyword (KW) or Abstract (ABS)**	functionalization OR catalys* OR activation	functionalization OR catalys* OR activation	functionalization OR catalys* OR activation	functionalization OR catalys* OR activation
**Connector**	AND	AND	AND	AND
**KW or ABS (M)**	direct*	arene*	review	iridium OR ir
**Connector (M)**	AND	AND	AND	AND
**KW or ABS (M)**	c-h OR c h	substrate OR starting material	overview	ligand* OR complex*

The resulting publication data from Scopus was downloaded as comma-separated value files (.csv), which contained information on citation, bibliography, abstract, keywords and funding details for each record. In a similar process, extraction of full records (information density similar to Scopus) from Web of Science searches as an Excel file (.xls) took place. The download of the reference data in SciFinder required additional manual efforts as only 100 references are downloadable at once in Excel format (.xlsx). Therefore, upon completing the downloads for one search tree, all excel files were combined into one sheet.

The downloaded data was subjected to a custom-built Alteryx Designer (Irvine, US) data curation (2) workflow that removed duplicates, added information from other databases, *e.g.*, journal impact factor, and carried out further filtering as well as calculations before splitting the publications into four quadrants based on journal impact factor and citations per year (Figure S1). After the removal of duplicates, 1723 unique publication records were identified, highlighting the broad and comprehensive search, which reduces the error of not including a relevant publication. Upon additional filtering for the presence of borylation and LSF-related keywords within

Table S3: Additional search queries (M2-3, S2-7, R2-4, C2-5), only showing the two modified sections.

Query number	Methodology	Starting Material	Review	Catalytic System
**2**	undirect* AND c-h OR c h	aromat* AND substrate OR starting material	review AND overview	rhodium OR rh AND ligand* OR complex*
**3**	ligand* OR complex* AND c-h OR c h	aliphat* AND substrate OR starting material	article OR method*	copper OR cu AND ligand* OR complex*
**4**	-	benzyl* AND substrate OR starting material	article AND method*	iron OR fe AND ligand* OR complex*
**5**	-	*sp^2* AND substrate OR starting material	-	no and metal OR metalfree OR metal AND free
**6**	-	*sp^3* AND substrate OR starting material	-	-
**7**	-	aryl* AND substrate OR starting material	-	-

the title and the abstract, 938 publications remained in the data set. With this data, various different clustering approaches could have been carried out using a selection of the following dimensions, *e.g.*, journal and affiliation, citations, journal impact factor, technologies, catalysts, starting materials, and publication year. For this research, clustering by citations per year over journal impact factor to determine the most relevant borylation methodology publications (high citations/year, high journal impact factor) was chosen. Removal of review papers delivered 242 remaining records, which underwent manual analysis to guarantee that the papers are within the scope of the automated HTE system (*e.g.*, photochemistry not yet possible). All deselected publications received a tag containing the reason to allow the usage of these records for other purposes in future without re-initiating the manual selection process. The final set of methodology papers contained 38 records, [2–40] which were subjected to reaction data extraction (3) in the next step. Figure S1 illustrates the first two steps of SACT including the results obtained for the borylation literature methodology search campaign.

While there are multiple ongoing efforts and ideas on how to establish a FAIR, simple and standardized format for reaction data documentation, today, methodologies are still reported in a multitude of different, usually not machine-readable structures. [41, 42] Therefore, full manual extraction of the data from reaction schemes or tables was conducted and a suitable database structure that captures this relevant information of a chemical transformation was determined. Rather than recording the pure minimum, all available data was stored. In this course, the simple user-friendly reaction format (SURF) convention, a simple, yet fully comprehensive and variable format, to document and store reaction data in a tab-delimited format, was developed. More details on SURF are shared in the respective section (see Section SI7). While SciFinder and Reaxys are helpful resources to obtain certain information concerning the chemical transformation, they are missing important details, such as equivalents or reaction concentration. Therefore, those properties were sourced manually from the paper, while unique identifiers (CAS numbers or SMILES) of reaction components could mostly be obtained through SciFinder or Reaxys. In addition, yield types (*e.g.*, isolated, GC-MS or NMR) and analytical data were documented. This labour-intensive work resulted in a high-quality data set comprising 1301 borylation reactions serving as an ideal foundation for informed plate design based on data analysis and chemical understanding. Moreover, due to the flexibility of the SURF format, the data was readily available as input for machine learning pipelines.

In the final step of the SACT methodology (4), the reaction data underwent analysis on various measures. Statistical evaluations of conditions, such as temperature or reaction time, as well as equivalent ratios, were complemented by an in-depth chemical and frequency interpretation. This included *e.g.*, mapping ligands with starting materials to determine what type of functional groups can be transformed by which ligands. The main important outcomes of this analysis, *i.e.*, those used for the informed plate design, can be found in Section SI4.

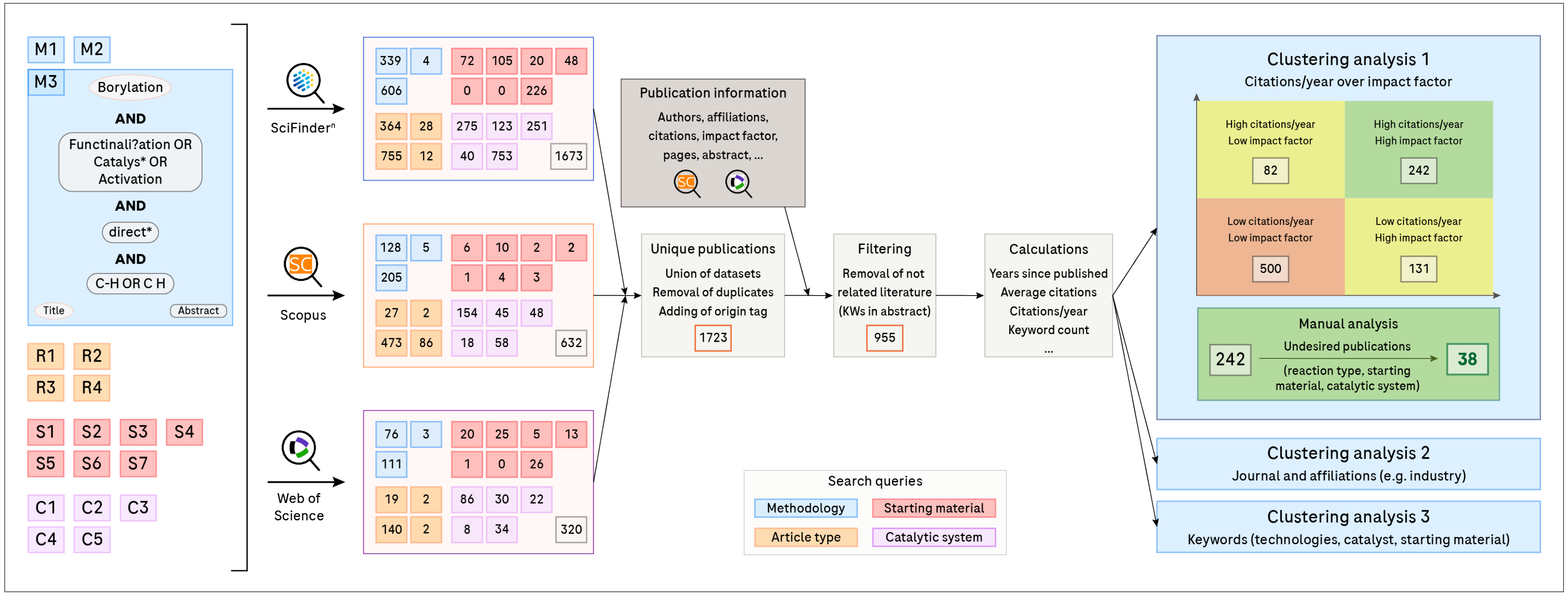

Figure S1: Literature search (SACT 1) followed by curation and evaluation of the obtained data (SACT 2).

SI3 LSF informer library

The substrates for the reaction screening and data generation were chosen through the agglomerative clustering method (a subtype of hierarchical cluster analysis), [43] of 1174 approved and accessible drugs obtained from Cortellis Drug Discovery Intelligence (Clarivate Analytics, Philadelphia, USA), and a molecular weight between 200 and 800 g/mol. The molecules were encoded using a similarity matrix of the Jaccard similarity of the ECFP4 [44] descriptors. Thereby, the obtained similarity matrix consisted of the dimensions NxN, where N equals the number of drugs in the similarity matrix. The similarity matrix was clustered into eight clusters from where the ten closest molecules to the cluster centre were picked using the cosine distance. 3 / 10 were then selected for the case study based on commercial availability and chemical meaningfulness. From this selection of 24 drugs (**1, 14-36**), 23 arrived within the required time frame. Figure S2 illustrates the investigated chemical space *via* principal component analysis (PCA) and Figures S3-S4 the selected drugs.

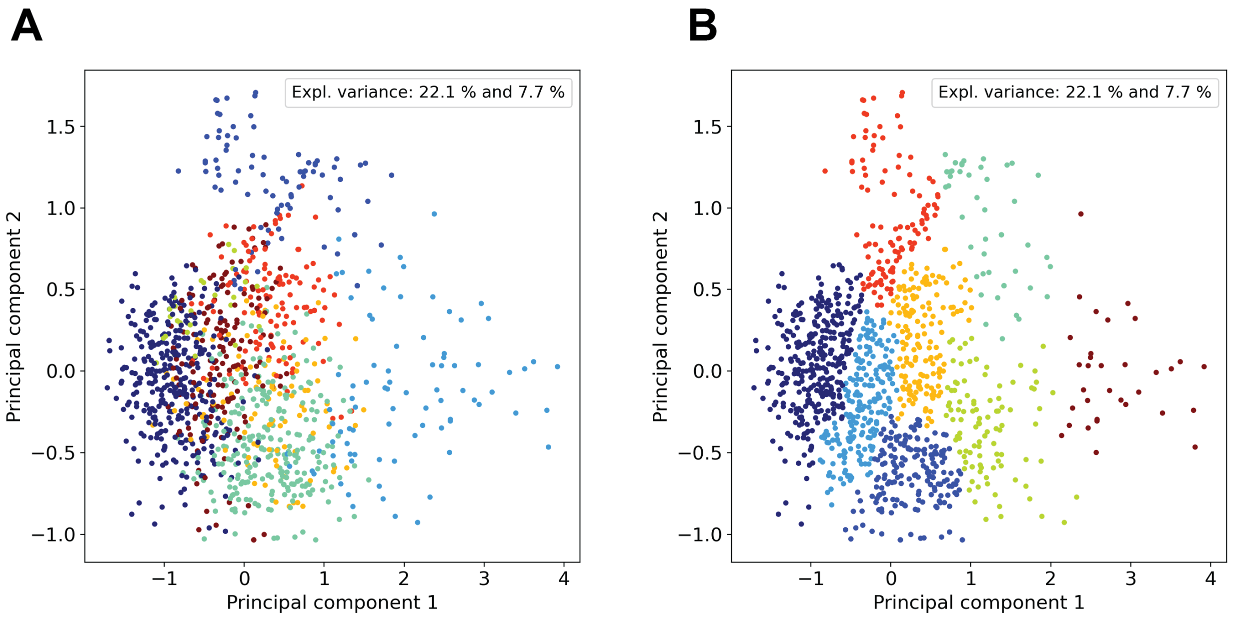

Figure S2: **Clustering**. **A** Principal component analysis (PCA) into principal component (PC) 1 and 2 of the 1174 drugs grouped into the calculated eight clusters in all dimensions. **B** PCA into PC 1 and 2 of the 1174 drugs grouped into the calculated eight clusters in the two reduced dimensions of the PCs. The explainable variance for the investigated data set in the first two PCA is 22.1% for PC1 and 7.7% for PC2.

To provide the model with fragments that are relevant to Roche's chemical space, the top 100 most popular ring assemblies in compounds of the Roche corporate compound collection were determined first. For these assemblies, substructure searches in the entire database were performed. The resulting compounds were kept if the structures had a molecular weight of less than 300 and or less than 20 heavy atoms, if there was at least 1 g of powder stock available and if the structures were not involved in any internal project or subject to legal restrictions. 268 fragments that fulfilled these criteria were identified. Out of this pool of candidates, 16 fragments (**37-48**) were manually selected by the authors. The manual selection aimed at incorporating a variety of frequently occurring functional groups and substituents in medicinal chemistry to test the broader applicability of the methodology. [45–47] Thus, fragments carrying halogen atoms (F, Br, Cl) or OH groups on the aromatic ring were chosen. Furthermore, the selection aimed to cover frequently used heterocyclic elements, such as pyridines, pyrazoles, thiazoles, morpholines, and benzimidazoles. Moreover, five idealized substrates were picked from the literature data set (**49-53**). All screened fragments and idealized substrates are depicted in Figure S5.

1

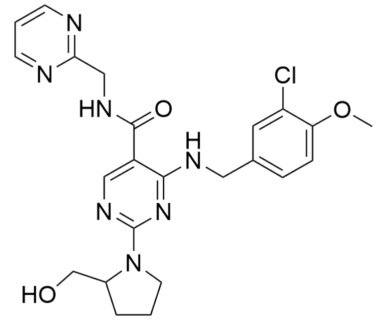

14 (Avanafil)

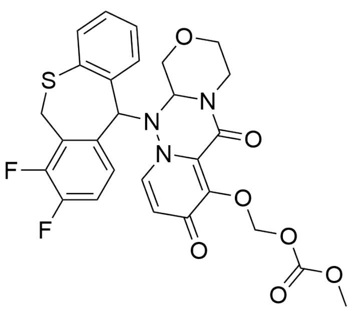

15 (Baloxavir marboxil)

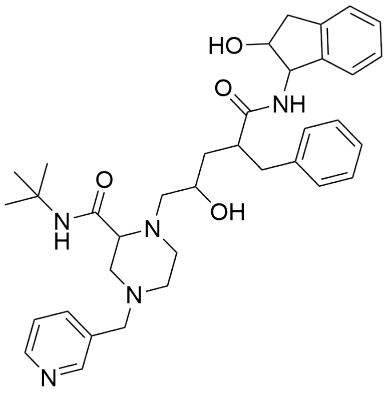

16 (Indinavir)

2

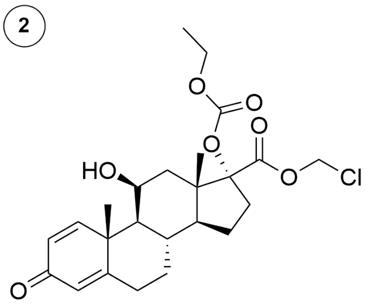

17 (Loteprednol etabonate)

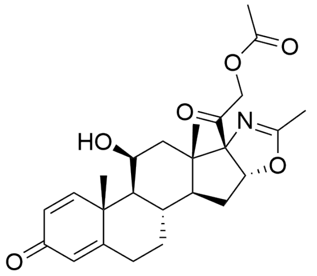

18 (Deflazacort)

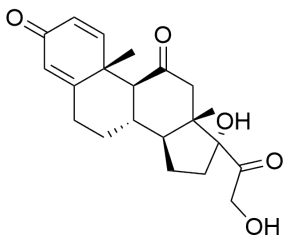

19 (Prednisone)

3

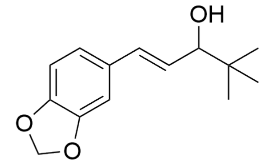

20 (Stiripentol)

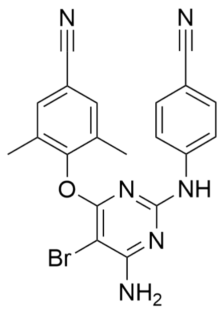

21 (Etravirine)

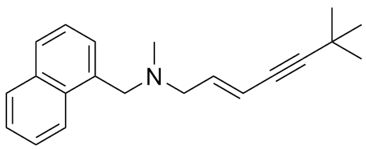

22 (Terbinafine)

4

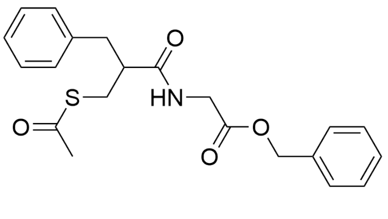

23 (Racecadotril)

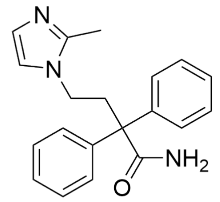

24 (Imidafenacin)

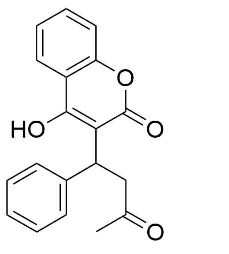

25 (Warfarin)

Figure S3: Selected examples from drug clusters 1-4. *Note: **15** did not arrive in time and was excluded from the study.*

5

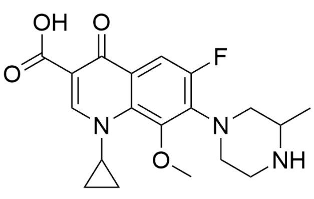

26 (Gatifloxacin)

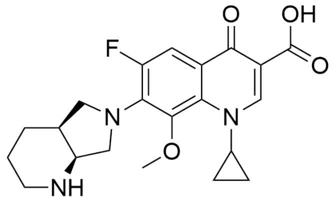

27 (Moxifloxacin)

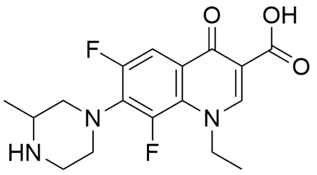

28 (Lomefloxacin)

6

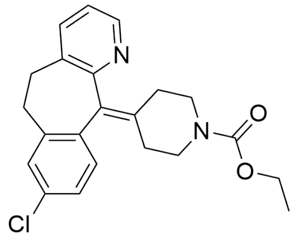

1 (Loratadine)

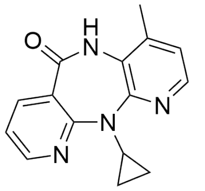

29 (Nevirapine)

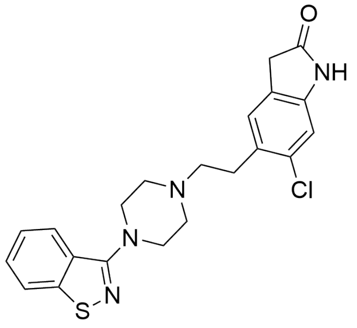

30 (Ziprasidone)

7

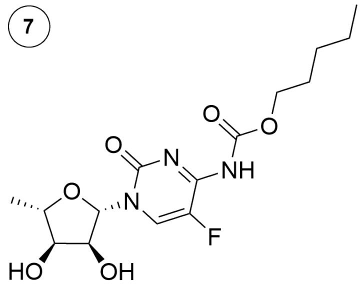

31 (Capecitabine)

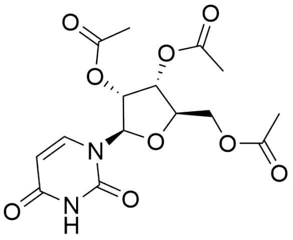

32 (Uridine triacetate)

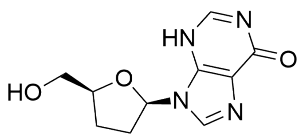

33 (Didanosine)

8

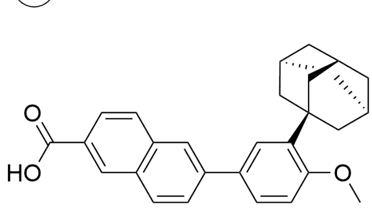

34 (Adapalene)

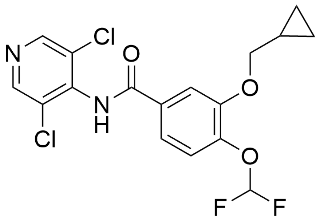

35 (Roflumilast)

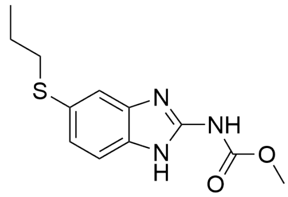

36 (Albendazole)

Figure S4: Selected examples from drug clusters clusters 5-8.

Fragments

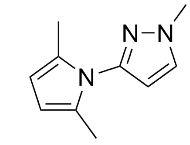

37

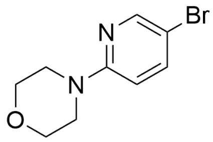

38

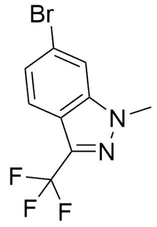

39

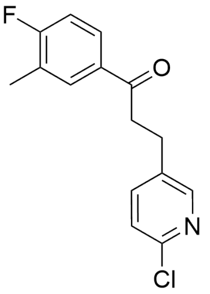

40

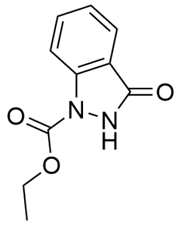

41

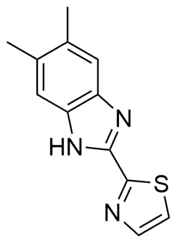

42

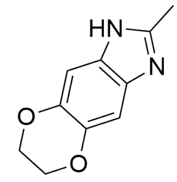

43

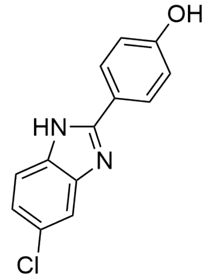

44

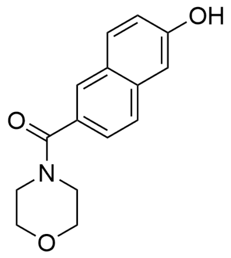

45

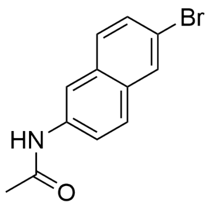

46

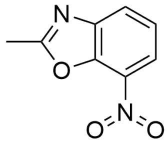

47

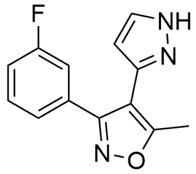

48

Idealized substrates

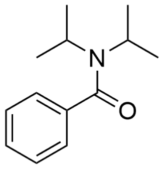

49

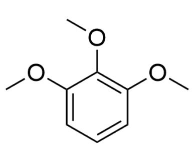

50

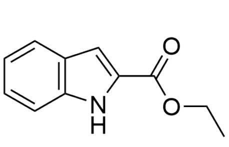

51

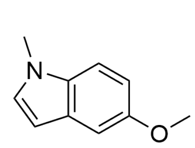

52

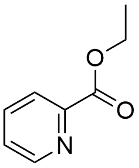

53

Figure S5: Screened fragments (**37-48**) and idealized substrates (**49-53**).

SI4 Screening plate design

To possess a clear rationale for the design of the screening plate, a statistical analysis, also referred to as meta-analysis, of the extracted reaction data was performed. As an initial starting point, the number of reaction components was determined. The largest number of C-H borylation reactions within the data set are constituted of four components in addition to the starting material: catalyst, ligand, reagent (boron source) and solvent. While there are examples with additives or additional reagents, initially it was aimed to reduce the complexity of a general screening plate and, therefore, only four component transformations were analyzed in detail. Further, it was opted for a 24-well plate design to reduce the time required for solid dispensing and limit the amount of required starting material (drugs, fragments) to a minimum. Future screenings to expand the data set with further reaction components are envisaged but will require reaction miniaturization and a flexible plate set-up. This could also include catalysts and ligands, which are not commercially available and were excluded from this initial study.

SI4.1 Reaction conditions

As reaction conditions are in general numerical values, a statistical analysis of the following important parameters was carried out: Reaction temperature (T) in °C, reaction time (t) in hours (h), reaction concentration (c) in mol/L and scale (n) in mmol. The plots showing the value distribution including the average and median for all four parameters are depicted in Figure S6.

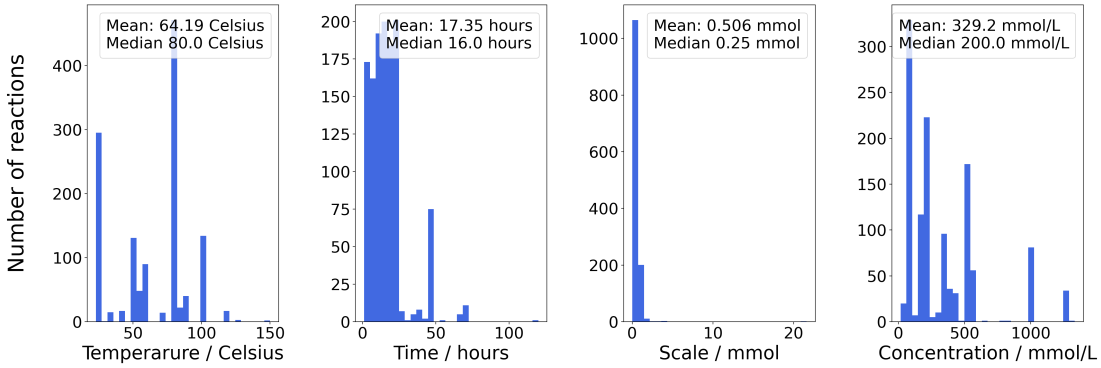

Figure S6: Core reaction parameter (T, t, c, n) distribution of literature data set.

Based on a calculated average (64.2 °C) and median temperature (80 °C) in the data set, 80 °C was selected as the reaction temperature for the 24-well plate. Determination of the average (17.4 h) and median (16.0 h) reaction time strongly indicated, running the reaction overnight for 16 hours. The reaction concentration median was found at 0.2 mol/L and used as molarity for the screening protocol. A lower scale (0.1 mmol) compared to the values (average: 0.51 mmol, median: 0.25 mmol) calculated from the data set was chosen to reduce material consumption. Moreover, the atmosphere under which the borylation reactions were performed, was analyzed. The literature data impressively showed that working under an argon or nitrogen atmosphere is preferred, which was also taken into account for the storage of the reagents. This observation can be explained due to the usage of oxygen and moisture-unstable Iridium catalysts.

SI4.2 Catalyst

Based on the data set (Figure S7), the top three catalysts used for borylation reactions have shown to be $[Ir(COD)OMe]_2$ (**2**, CAS: 12148-71-9), $Pd(OAc)_2$ (**58**, CAS: 3375-31-3) and $[Ir(COD)Cl]_2$ (**59**, CAS: 12112-67-3). All three catalysts are commercially available and would be suitable for the desired borylation screening. Nevertheless, **2** (N = 813) has been used 10-fold more compared to **58** (N = 74) and **59** (N = 47). Therefore, **2**

was chosen as the single catalyst for the screening plate. Based on the data set, the average catalyst loading in borylation reactions in relation to the starting material is 3 mol% with the median being 1.5 mol%. A value in the middle of both values was selected, leading to a catalyst loading of 2.5%.

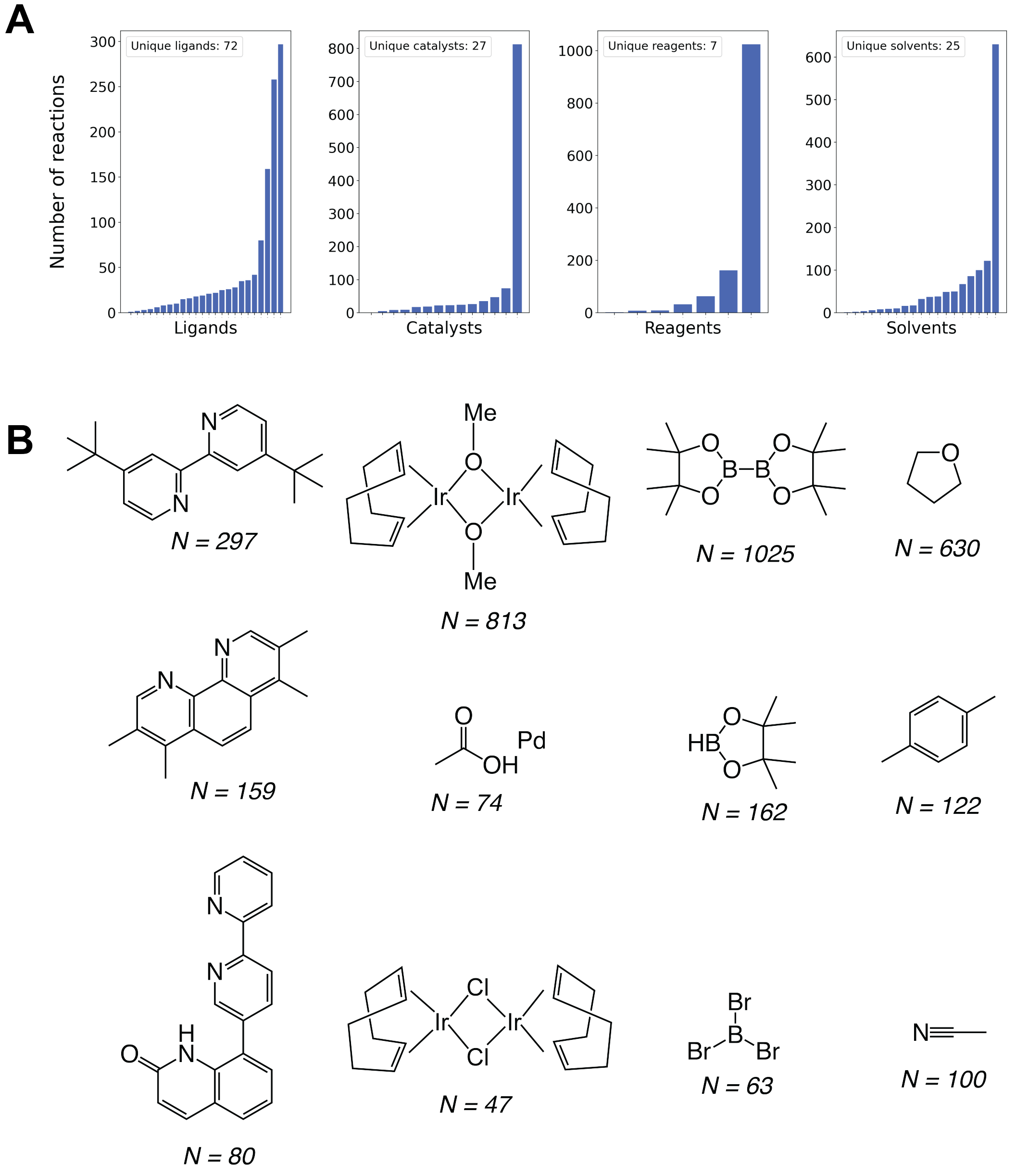

Figure S7: Analysis of the reaction components of the 1301 reactions of the literature data set. **A** Barplot illustrating the abundance of different reaction components. From left to right: ligands, catalysts, reagents, and solvents. Each bar illustrates one unique species, and the bars are sorted from least abundant (left) to most abundant (right) **B** The three most abundant species for each of the reaction components. From left to right: ligands, catalysts, reagents, and solvents. *Note: The second highest count for ligands in "none" (i.e., a ligand free reaction). Therefore, the top-3 and top-4 most abundant ligands are shown instead.*

SI4.3 Ligand

Overall, the most used ligand (N = 297) is dtbbpy (**6**, CAS: 72914-19-3), which was also used in combination with **2** in 256 reactions. The second most abundant ligand is tmphen (**9**, CAS: 1660-93-1), which has been used 159 times across the full data set and 127 times in combination with **2**. From the top twelve ligand combinations with **2**, only six are commercially available. Those six ligands were selected for the screening plate. In addition to the above-mentioned dtbbpy and tmphen, 2-pyridinecarboxaldehyde 2,2-bis(phenylmethyl)hydrazone (**4**, CAS:

237402-29-8), 8-aminoquinoline (**5**, CAS: 578-66-5), 4,4'-dimethyl-2,2'-bipyridine (**7**, CAS: 1134-35-6) and 1,10-phenanthroline (**8**, CAS: 66-71-7) are included into the screening plate (main paper, Figure 2a). The analysis of the ligand/catalyst ratio revealed an average of 1.59 and a median of 2, the median was used to give a 5 mol% ligand loading.

SI4.4 Reagent / boron source

B_2pin_2 (**3**, CAS: 73183-34-3, N = 1052) and HBpin (**60**, CAS: 25015-63-8, N = 162) have been the most used reagents (boron sources) across the data set, with BBr_3 (**61**, CAS: 10294-33-4, N = 63) complementing the top three. As **61** is part of a different chemistry type (metal-free borylations), only **3** and **60** were analyzed in more detail. In combination with **2**, **3** (N = 710) was used nearly nine times more often than **60** (N = 71) allowing an informed selection decision to utilize **3** as the boron source for the 24-well plate. On average, a slight excess of **3** (1.25 equivalents) was used in the analyzed reactions. The median, though, shows an equimolar in relation to the starting material (eq = 1), which was chosen for the plate design.

SI4.5 Solvents

The most used solvents have been shown to be aprotic solvents that are mainly non-polar or only slightly polar. The most used solvent by far is THF (**62**, CAS: 109-99-9, N = 630), followed by *p*-xylene (**63**, CAS: 106-42-3, N = 122) and acetonitrile (**13**, CAS: 75-05-8, N = 100). With a reaction temperature of 80 °C, **62** (boiling point: 66 °C) is not an ideal solvent if potential evaporation should be avoided. Instead, 2-methyltetrahydrofuran (**11**, CAS: 96-47-9, boiling point: 80 °C) was chosen due to the higher boiling point while maintaining key properties (*e.g.*, polarity), even though it did not appear in the data set. Due to the potential of solvent borylation, **63** was avoided, but the number three solvent **13** was included in the screening plate. Furthermore, CPME (**12**, CAS: 5614-37-9, N = 31) and cyclohexane (**10**, CAS: 110-82-7, N = 38) were selected due to their high boiling points and their regular appearance in the data set. All solvents, except **11**, were also used in combination with **2**.

SI4.6 Plate design

Based on the considerations above, the plate design was implemented and is shown in Figure 2a of the paper.

SI5 HTE borylation screening protocol

All generated screening data used the plate design depicted in the paper (Figure 2a) and the procedure below, only the starting materials (SI3) were varied. In a nitrogen-filled glovebox from mbraun (Garching, DE) that does not contain any liquids, all solid reaction components were dosed into 1 mL glass vials from Analytical Sales (Flanders, US) on a 24- or 96-well plate from Analytical Sales (Flanders, US) using a CHRONECT Quantos from Axel Semrau GmbH & Co. KG (Spockhövel, DE) coupled with an XPE206 balance from Mettler Toledo (Greifensee, CH). The plate was sealed and discharged from the glovebox before being transferred to another glovebox from LC Technologies (Salisbury, US), where solvents were added to the vials using multichannel pipettes from Eppendorf (Hamburg, DE). The plate was heated within the glovebox (LC Technologies) on a Junior benchtop solution from Unchained Labs (Pleasanton, US) and VP 721F-1 Parylene Encapsulated Stainless Steel Stir Discs from V&P Scientific Inc. (San Diego, US) were used to stir the reaction mixture. For an intermediate internal process control (IPC), samples were drawn from the plate within the glovebox using a multichannel pipette and transferred into a new plate, which was then subjected to a Genevac centrifugal evaporator EZ3P-VVVHz-HN0 from SP Industries (Warminster, US) to remove the solvents. For a single or the final IPC, the plate was cooled and discharged from the glovebox before being placed into the Genevac centrifugal evaporator to remove the solvents. Using a Freedom EVO 100 liquid handler from Tecan (Männedorf, CH), the residues were re-suspended in MeCN/H_2O (4:1) and shook on a Teleshake 95 from Inheco (Martinsried, DE). Depending on the concentration of the suspension, further dilution steps using the Tecan liquid handler were carried out to reach an LCMS injection concentration of 1 or 0.5 mmol/L. Finally, the samples were transferred onto a 96-deep-well plate (1 mL) from Eppendorf (Hamburg, DE). The plates were analyzed on a Waters (Milford, US) UPLC-MS system equipped with a Waters Acquity sample manager with a flow-through needle, a Waters Acquity sample organizer and a Waters QDa single quadrupole mass spectrometer. The separation was achieved on a ZORBAX RRHT Eclipse Plus C18, 95 Å, 2.1 x 30 mm, 1.8 µm column (P/N 959731-902, LOT: USUXY02479) from Agilent (Santa Clara, USA) at 50 °C. A 2-minute gradient was used and the injection volume accounted for 2 µL. 2 min gradient: A: 0.1% HCOOH in H_2O; B: 0.07% HCOOH in MeCN at flow 1 mL/min. Gradient: 0 min, 3% B; 0.2 min, 3% B; 1.5 min, 97% B; 0.3 min, 97% B; 0.1 min 3% B. The raw data were processed with MassLynx V4.2 and the obtained .rpt file underwent parsing with a customized script, before being subjected to the automated reaction data analysis pipeline (SI6). Due to irrevocable data loss by the LCMS, 956 instead of 960 experimental data points (40 substrates x 24 conditions) were collected.

SI6 Automated reaction data analysis pipeline

Figure S8 illustrates the automated reaction data analysis pipeline used to rapidly identify if drugs, fragments and idealized substrates were borylated or not. Each reaction carries a unique identifier, which is reflected through the LCMS sample name and the MS searches for the sum formulas of the desired products (mono- and diborylated boronic ester and acid). The LCMS-measured data are reported into a .rpt file that needs to undergo parsing to allow a transfer into a tabular format. The obtained data is then pushed to a server from which it is accessible through multiple means. In this case, Alteryx Designer (Irvine, US) was chosen for further processing of the data. In the first step, the data stream is cleaned to remove any undesired columns that would slow down the pipeline. As the LCMS delivers a three-channel output (LC, ES+, ES-), those need to be connected for the same peak in order to allow quantitative and qualitative assessment of the peak. In addition, the Sample ID is disassembled to obtain the different identifiers required for the upcoming data curation.

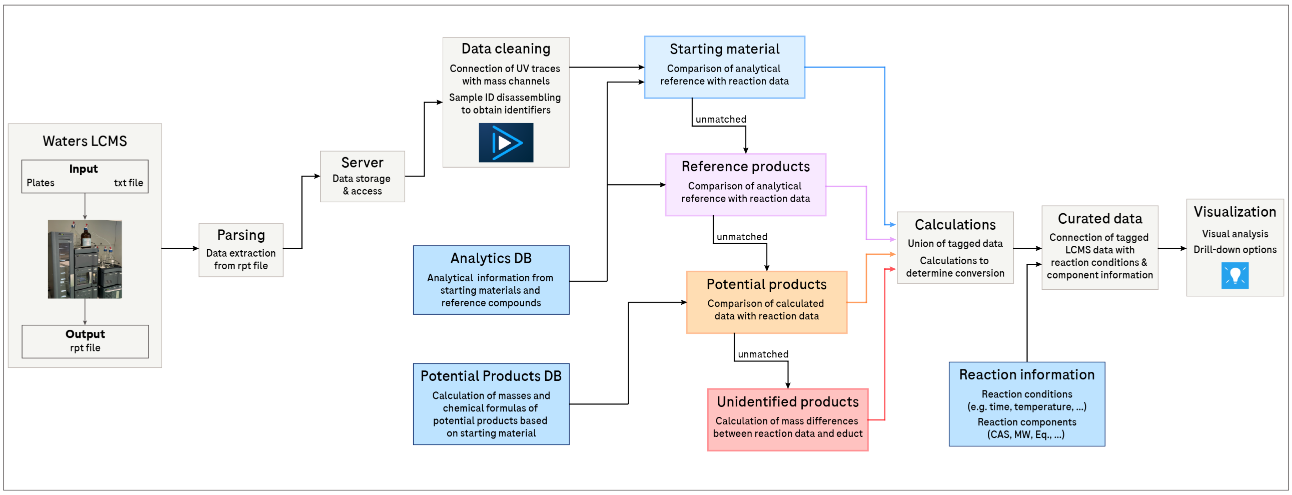

Figure S8: Simplified schematic overview of the automated reaction data analysis pipeline.

In addition to the reaction mixtures, all starting materials and, if available, reference products using the same solvent mixture (MeCN:H_2O, 4:1) are measured on the LCMS to obtain the retention time (LC) and mass pattern (MS). This data is stored in a database and needed for the initial two steps of the matching process. More relevant for LSF though, are the desired/potential products of the reaction. Those masses and chemical formulas are calculated based on the starting material information and the transformation. This Alteryx workflow allows hands-free generation of the potential products including molecular weight, mono-isotopic mass and chemical formula (Hill notation). In addition to being used for the reaction data analysis, this data is also the foundation for generating the LCMS input file.

Once the reaction data has passed through the cleaning process, it is compared to the LCMS information from the above-mentioned data sets, starting off with the identification of the starting material. If a trace from the reaction mixture matches the retention time ($\pm$ 0.02 min) and the mass pattern (chemical formula detected, mass channel match with database reference), it receives the starting material tag. All unmatched traces continue through the pipeline, where reference compounds, if available, are tagged using the same criteria. The remaining data is then compared to the products that could potentially be formed and are desired (mono- or diborylated species). Since the exact position of the new functional groups is not known, no reference compounds are available. Therefore, only the five most abundant masses per peak are used for tagging and compared to information from the potential product database. Based on the abundance of the mass and if the chemical formula was found by the LCMS, the tag is complemented by an MS reliability score. The score is higher if the chemical formula was found and the correct mass of the desired product ($\pm$ 0.5 Da) appears in a more abundant channel. For this study, only high MS reliability scores were subjected to the machine learning platform. Last, the unmatched

data is classified as unidentified products, and the mass differences between the peak and parent material are calculated to avoid unnecessary manual calculating of mass differences.

After the tagging is completed, the data streams are recombined and subjected to calculations in order to quantify the reaction components from starting material through reagents to products. To do so, the sum of all LC peaks (integral) is calculated and each peak is then divided by this value. This gives a quantitative measure of the product distribution within the sample, an LCMS conversion. While there are numerous approaches to using internal standards or assays, due to the nature of LSF they have not been applied. LSF reactions tap into new, unexplored chemical space and generally, multiple different components are formed. Therefore, selecting an internal standard that does not overlap with one of these unknown components, is highly difficult.

Upon completion of the calculations, using the identifiers mentioned earlier, reaction information, such as conditions and components, are added to the components that have been identified and quantified. This follows the FAIR data principle and generates a curated, high-quality LSF screening data set that can be stored and shared in the SURF convention (SI7). This allows rapid subjecting of the data to machine learning algorithms as done in this research. It also allows direct visualization of the data in known interfaces, such as TIBCO Spotfire (Somerville, USA) or Tableau (Seattle, USA). Using this workflow, the data curation of one plate usually takes less than one minute.

SI7 SURF convention

The simple user-friendly reaction format (SURF) aims at standardizing reaction data reporting through a simple, yet comprehensive and structured format that is usable with a basic understanding of a spreadsheet. SURF does not require any coding experience, advanced IT skills or a web interface. It enables every chemist within or outside the lab to document chemical synthesis in a machine-readable and shareable format. SURF allowed extraction and documentation of the borylation reactions from literature faster. The generated reaction screening data were also transformed into SURF before being directly subjected to the machine learning pipelines. Reaction documentation following SURF can be implemented in every spreadsheet as the only requirement is the existence of rows and columns.

Each row of the spreadsheet represents the information and data for one single reaction. The SURF convention contains constant (CC) and flexible (FC) categories. CCs never change and are always present, independent of the number of reaction components. They capture the origin and ids of the reaction as well as basic characteristics (reaction type, named reaction, reaction technology) and conditions (temperature, time, atmosphere, scale, concentration, stirring/shaking). Add-ons, such as the procedure or comments, belong to the CCs, too. The FCs describe the more variable part of a reaction, the starting material(s), solvent(s), reagent(s) and product(s). Two identifier options (CAS and SMILES) are available for each component. While the SMILES string is available for every compound and serves as structural input for machine learning models, the CAS number, even though not always available, can be handy for chemists in the lab to order, itemize and find chemicals. For the starting material(s) and reagent(s), *e.g.*, catalyst, ligand, additive, the number and type of columns remain the same (CAS, Smiles, equivalents). If multiple starting materials or reagents are used, additional columns are required. In that case, the three information columns are duplicated and the X is replaced by a number, starting from 1 for the first component, 2 for the second, etc. The same accounts for multiple solvents or products, however, due to their role, they possess more and partly different columns. While the CAS number and/or the SMILES string remain as an identifier, the solvent fraction (in decimals) instead of equivalents is recorded. This allows exact determination of the ratio between solvents. The product category withholds the largest amount of headers as SURF records the yield (in percent), but also the yield type (*e.g.*, isolated, lcms, gcms) as well as the detected mass by MS and the ^{1}H NMR sequence in addition to the common identifiers CAS and Smiles. This not only allows rapid comparison when experiments are reproduced but can also deliver important increments for machine learning models by differentiating between yield types. As most electronic lab journals already record the above-mentioned parameters, by enforcing of documentation compliance combined with simple automated data extraction and cleaning pipelines, numerous reaction data could be accessible in the SURF convention, and readily available for machine learning applications. We spent thoughts on how to further reduce complexity by introducing specific SURFs without FCs for chemical transformations where the reaction components are generally the same. An excellent example would be Suzuki-Miyaura couplings that utilize a set of six to seven components (organoboron species, halide, catalyst, ligand, base, solvents). [48, 49] However, generating different tailored templates would ultimately end up in various different formats and mismatching headers falling short of the main SURF goal to standardize reaction documentation.

The results of this paper would have not been achieved without FAIR data handling using SURF. The manually extracted reaction data (1376 reactions from 38 publications), which were used in this manuscript for data analysis and selectivity prediction, reported in SURF are attached to the SI as a tab-delimited text file. Moreover, two empty SURF templates are attached as tab-delimited text files: The first file contains the general SURF template, which can be adjusted by introducing additional columns depending on the reaction specifics. The second file is a customized SURF template that should accommodate the vast of chemical transformations: It contains columns for two starting materials, two reagents, one catalyst, one ligand, one additive, two solvents and two products.

SI8 Further analysis of the experimental data set

SI8.1 Molecular property distribution

The molecular property distribution of the 40 molecules within the LSF space library for eight different molecular properties is visualized in Figure S9. Furthermore, the reaction yield distribution of both the complete experimental data set and only the positive results of the former are visualized in a histogram in Figure S10.

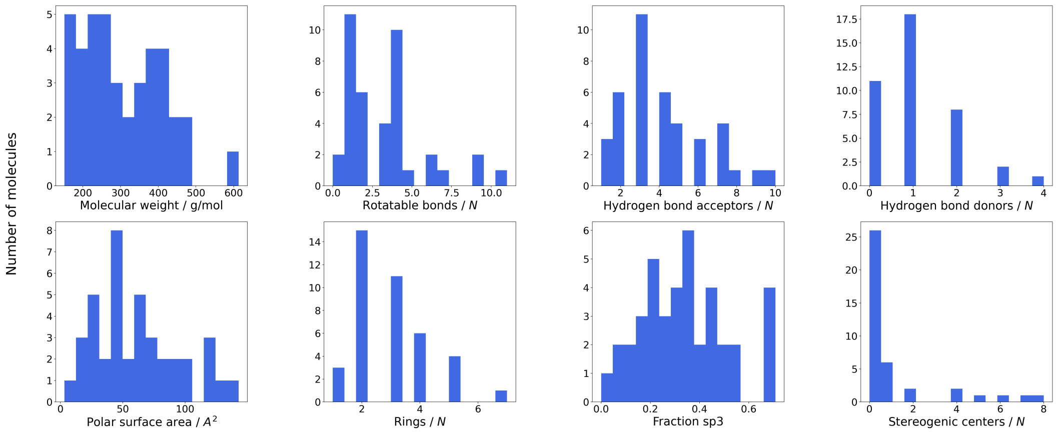

Figure S9: Molecular property distributions of the experimental data set. Top left to right: molecular weight, number of rotatable bonds, hydrogen bond acceptors, hydrogen bond donors; Bottom left to right: polar surface area, number of rings, sp^3 fraction, and number stereogenic centres.

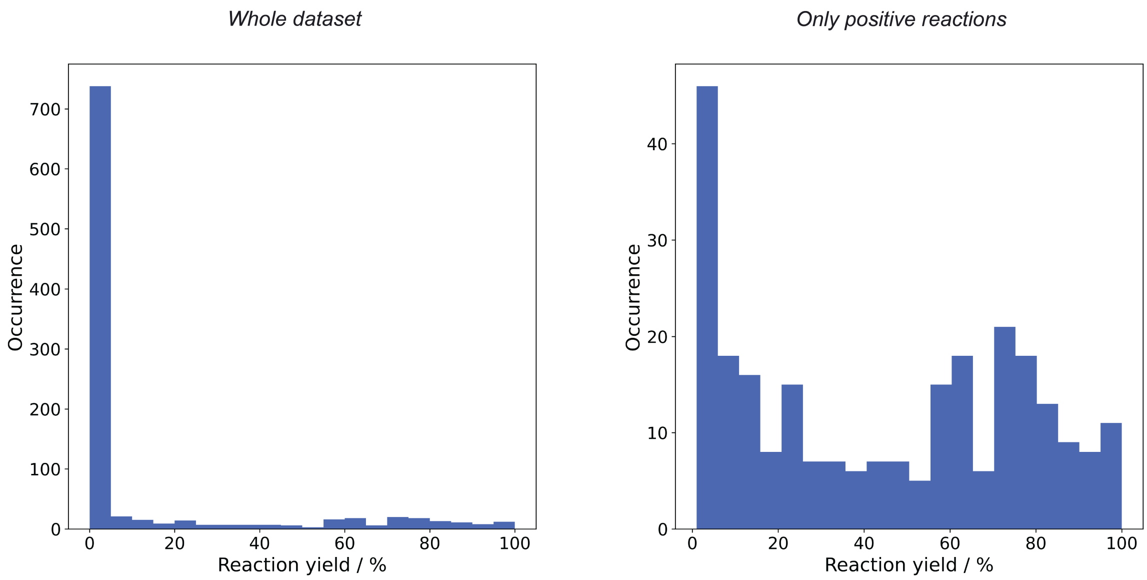

Figure S10: Histogram of reaction yield distribution of the experimental data set. Left: Reaction yield distribution on the whole dataset. Right: Histogram of reaction yield distribution of positive reactions.

SI8.2 Functional group analysis

Functional groups are known as chemical substructures in molecules that consist of atoms and bonds which are responsible for molecular properties such as reactivity or bioactivity. [50] The concept of functional groups has therefore formed a cornerstone in synthetic chemistry, medicinal chemistry and toxicology. [51] To evaluate the scope and limitations of our machine learning platform and the investigated borylation reactions, the functional groups covered by substrates in the LSF space library have been extracted and analyzed. A substructure-free algorithm has been used to extract functional groups from molecules. [52] The resulting functional groups from the LSF space library were compared to the ones from all 1174 drugs and analyzed towards their tolerance for successful borylation reactions. The 53 functional groups extracted from the LSF space library correspond to 11.6 % of the total 458 functional groups present in the 1174 drug molecules (Figure S11 A). However, of the 40 most abundant functional groups found within the drug molecules, 33 (82.5 %) are covered by the LSF space library. The top-3 most occurring functional groups in the LSF space library (40 molecules, including fragments) are aromatic nitrogens, aromatic alkyl-oxy groups and alcohols (Figure S11 B). Most abundant groups covered by the drug space but not by the LSF space (Figure S11 C) are alkyl carboxylic acids or esters (first and third orange bar from left to right, respectively), primary amines (second orange bar from left to right), and tertiary and secondary amides (fourth and fifth orange bar from left to right, respectively). Further, the functional groups which have shown to be tolerated or not tolerated were investigated. All occurring five- and six-membered aromatic heterocycles containing nitrogen, oxygen and sulfur are well tolerated or even cause the desired reaction outcomes (Figure S11 D). On the contrary, certain non-aromatic functional groups such as primary amines, carbamates and carbonates, or aromatic functional groups with strong electron-withdrawing moieties (*e.g.* nitro-aryls) are found to be less tolerated and inhibit desired reaction outcomes (Figure S11 D and E).

Further, Table S5 and S4 shows the number of successful reactions for the different solvents and ligands, respectively.

Table S4: Number of successful and failed reaction for the different ligands.

SMILES	Successful reactions / #	Failed reactions / #
N=1C=C(C(=C2C=CC3=C(N=CC(=C3C)C)C12)C)C	52	108
N=1C=CC(=CC1C=2N=CC=C(C2)C(C)(C)C)C(C)(C)C	48	111
N=1C=CC=C2C=CC=3C=CC=NC3C12	46	114
N=1C=CC(=CC1C=2N=CC=C(C2)C)C	46	113
N=1C=CC=CC1C=NN(CC=2C=CC=CC2)CC=3C=CC=CC3	35	124
N1=CC=CC2=CC=CC(N)=C12	27	132

Table S5: Number of successful and failed reaction for the different solvents.

SMILES	Successful reactions / #	Failed reactions / #
C1CCCCC1	80	159
O(C)C1CCCC1	68	171
O1CCCC1C	60	179
N#CC	46	193

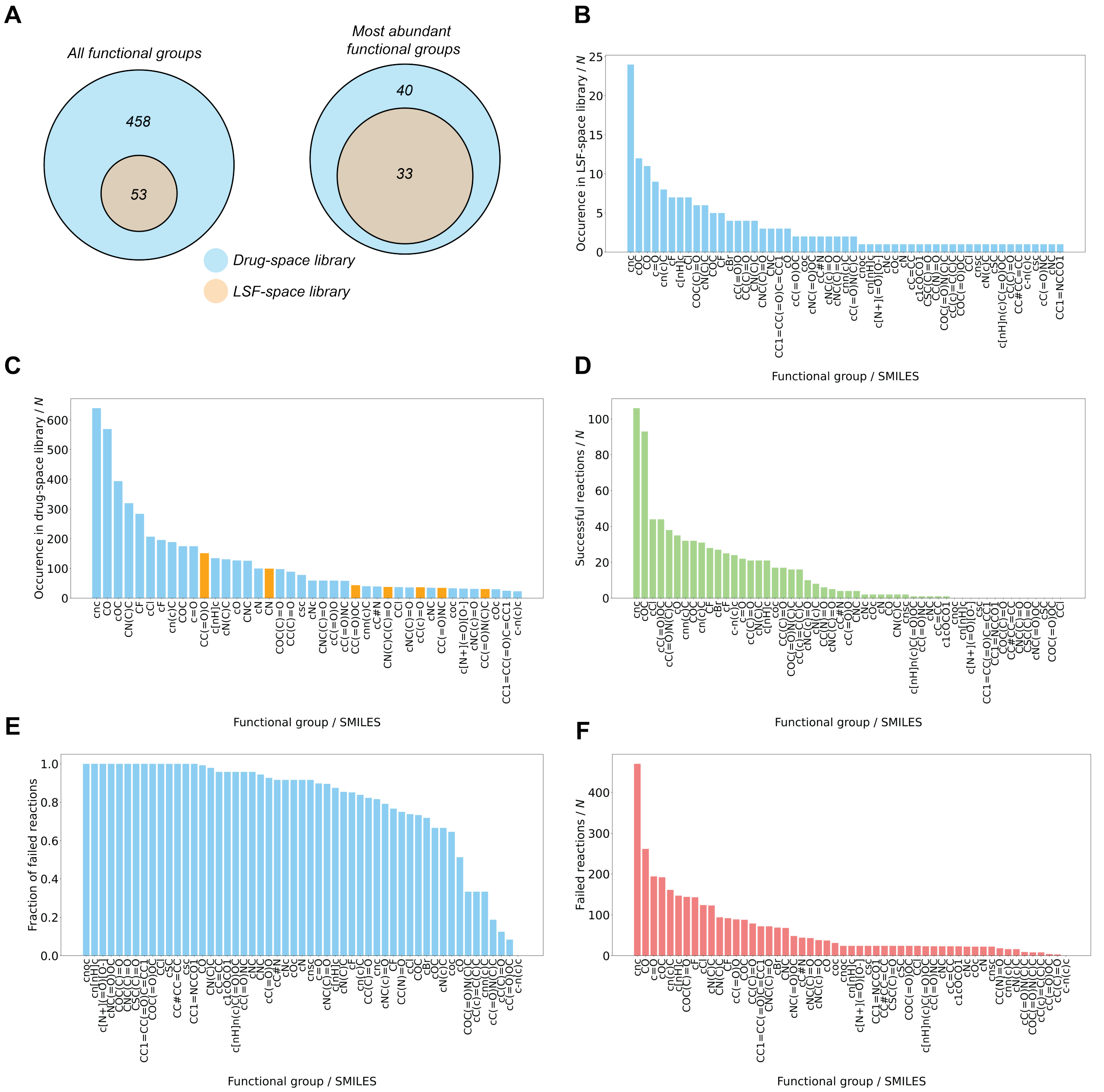

Figure S11: Functional group analysis. **A** Comparing the number of functional groups in the LSF space library to the ones in the drug space library. Left: All functional groups; right: The 40 most abundant functional groups. **B** The 53 functional groups extracted from the LSF space library are plotted by their occurrence from left to right. **C** The 40 most abundant functional groups extracted from the drug space library are plotted by their occurrence from left to right. The bars in blue (33/40) show the functional groups which are covered by the LSF space library. The bars in orange (7/40) show the functional groups which are missing in the LSF space library. **D** The 53 functional groups extracted from the LSF space library are plotted by the absolute number of successful reactions from left to right. **E** The 53 functional groups extracted from the LSF space library are plotted by the fraction of failed reactions from left to right. **F** The 53 functional groups extracted from the LSF space library plotted by the absolute number of failed reactions from left to right.

SI9 Further analysis of the literature data set

In the following, an additional analysis of the experimental data set is described. The molecular property distribution for eight different molecular properties is visualized in Figure S12. Figure S13 shows the reaction yield distribution. To learn the reaction yields the reactions have been binned into four different equally sized bins in the ranges of 0-45%, 45-65%, 65-83%, and 83-100%.

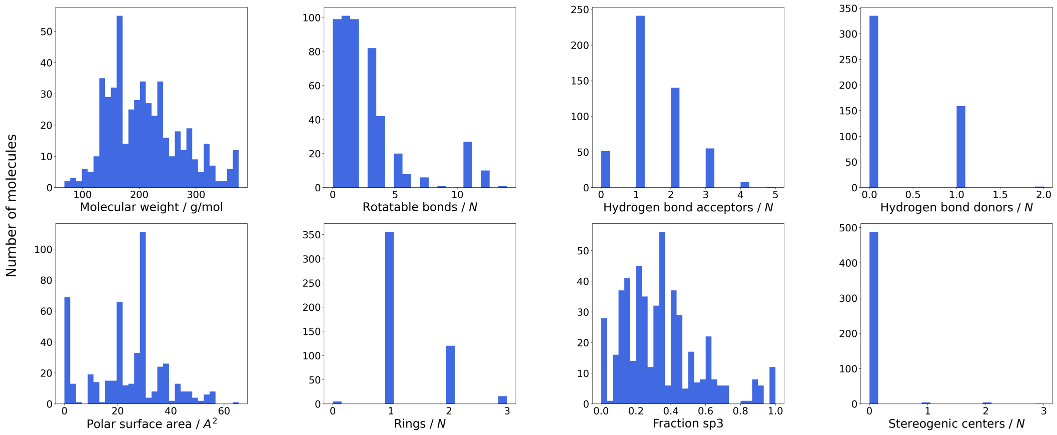

Figure S12: Molecular property distributions of literature data set showing from top left to bottom right: molecular weight, rotatable bonds, hydrogen bond acceptors, hydrogen bond donors, polar surface area, rings, sp^3 fraction, and stereogenic centres.

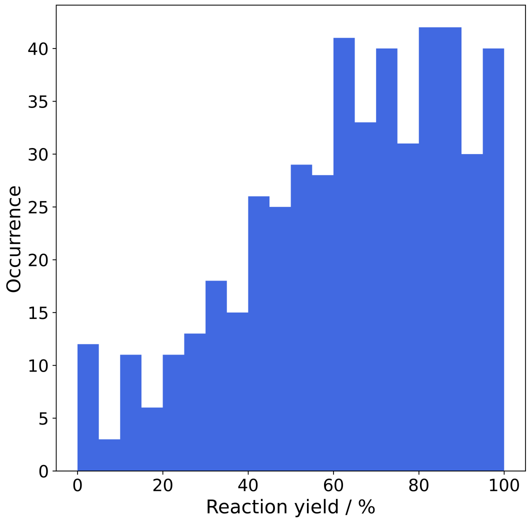

Figure S13: Reaction yield distribution of the literature data set.

SI9.1 Diversity analysis for regioselectivity data set

To further assess the diversity of the chemical space in the regioselectivity training data, the starting materials were clustered using sphere exclusion clustering on ECFP4 fingerprints using a Tanimoto threshold of 0.55 with the ChemFP toolkit [53]. To do so, starting materials were first desalted and standardized using RDKit v.2020.03.1 [54] and unique molecules were kept based on InChI keys. 656 unique starting materials remained, for which the clustering results are shown in Figure S14. Overall, 119 compound clusters and 209 Bemis-Murcko scaffolds were obtained by performing this analysis (Table S6). We argue that this is a sufficiently diverse representation for the task of interest and exceeds the chemical diversity observed in a recent pre-print [55] (Figure S7). As molecular shape potentially influences the performance of the regioselectivity prediction, principal moment of inertia plots and the fraction of sp^3 carbons were further calculated using RDKit (Figure S15). We found that the three-dimensionality of the data is in the range of structures typically observed in medicinal chemistry projects [56].

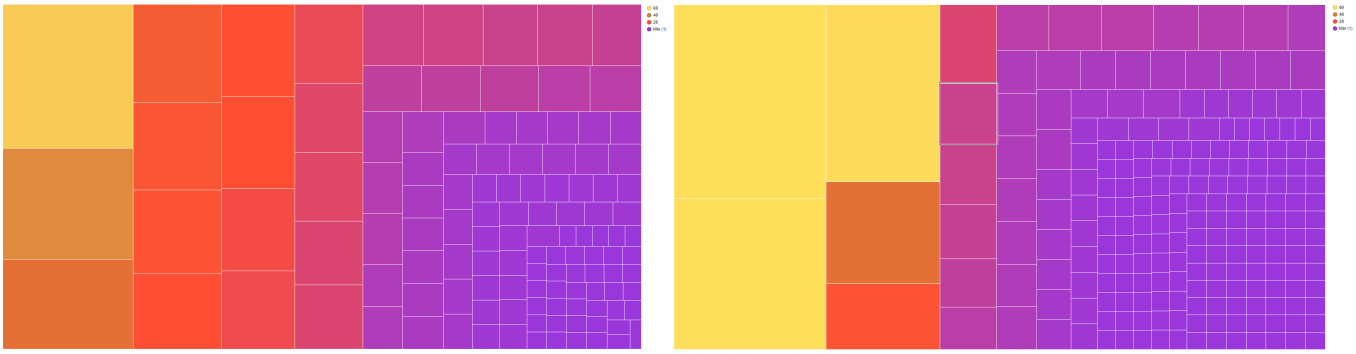

A: Compound Clusters **B: Bemis-Murcko Scaffolds**

Figure S14: A / left: Tree map showing the size of the sphere exclusion clusters obtained for the regioselectivity training data when clustering on the whole molecular structure. B / right: Number of compounds per Bemis-Murcko scaffold. The size of the boxes as well as the color represents the number of compounds. For the 656 molecules, 119 clusters were obtained on the molecule level and a total of 209 scaffolds were observed. The largest molecule cluster contained 56 members and the most frequent scaffold had 86 compounds.

Table S6: Number of sphere exclusion clusters per cluster size (left) and number of compounds per Bemis-Murcko scaffold (right) observed for the regioselectivity training data.

**Compounds per Cluster**	**Count**
1	40
2	24
3	16
4	7
5	3
6	3
7	2
8	3
9	1
10	2
11	2
13	2
14	2
16	1
17	1
18	1
20	3
22	1
23	1
26	1
35	1
43	1
56	1
**Total Clusters**	**119**
**Total Compounds**	**656**

**Compounds per Scaffold**	**Count**
1	146
2	19
3	9
4	9
5	9
6	3
7	4
8	1
9	1
10	2
13	1
22	1
34	1
59	1
67	1
86	1
**Total Scaffolds**	**209**

Table S7: Number of sphere exclusion clusters per cluster size (left) and number of compounds per Bemis-Murcko scaffold (right) observed in [55].

Compounds per Cluster	Count
1	70
2	8
3	7
4	1
5	1
7	1
10	1
12	1
**Total Clusters**	**90**
**Total Compounds**	**145**

Compounds per Scaffold	Count
1	91
2	9
3	5
5	1
8	2
**Total Scaffolds**	**108**

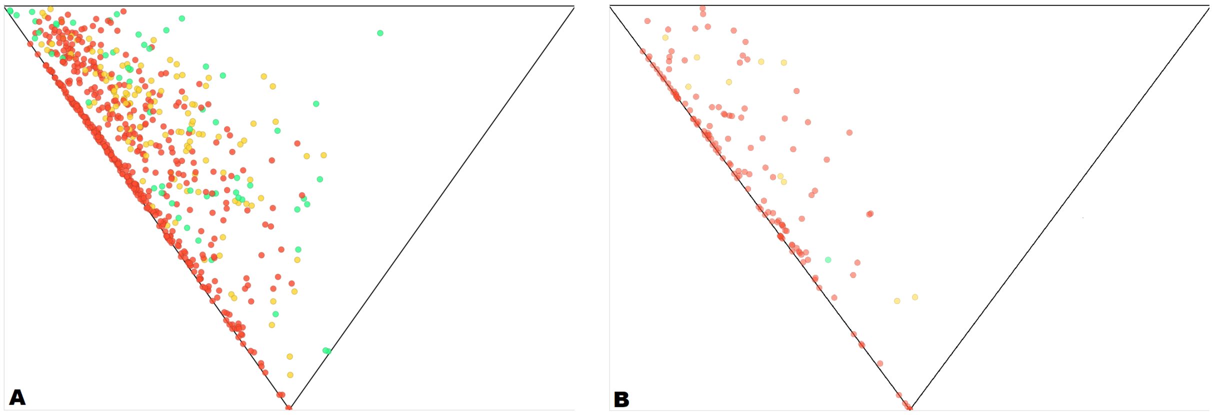

Figure S15: Principal moments of inertia plot representing the shape of the regioselectivity training data for this publication (A/left) and a recent pre-print (B/right) [55]. Dots represent compounds and the color represents the fraction of sp^3 carbons, with red $\leq$0.3, green $\geq$0.5 and yellow in between. Rod-shaped compounds appear in the top left, disc-shaped compounds in the bottom and sphere-shaped compounds in the top right corner.

SI9.2 Model performance on the literature data set

Table S9 and S10 show the accuracy of the investigated nine neural networks. The performance of the reaction yield predictions was investigated on a randomly split data set to learn reaction yields for known substrates in combination with new conditions for both the literature data set (Figure S16).

Table S8: Model performance of the nine investigated neural networks predicting binary reaction outcomes and reaction yields. Mean absolute errors (MAEs) were used to quantify reaction yield predictions. Area under receiver operating characteristic curve(AUC) was used to quantify binary reaction outcome predictions. The numbers represent mean and standard deviation for N=3 independent neural network runs.

	**Reaction yield, PCC**	**Reaction yield, MAE / %**
**GTNN2D**	0.59 (±0.01)	4.53 (±0.09)
**GNN2D**	0.61 (±0.01)	5.61 (±0.06)
**GTNN3D**	0.62 (±0.01)	4.51 (±0.11)
**GNN3D**	**0.63 (±0.01)**	5.33 (±0.34)
**GTNN2DQM**	0.62 (±0.01)	4.41 (±0.17)
**GNN2DQM**	0.61 (±0.01)	5.41 (±0.10)
**GTNN3DQM**	0.61 (±0.01)	**4.23 (±0.08)**
**GNN3DQM**	0.62 (±0.01)	4.88 (±0.24)
**ECFP4NN**	0.530(±0.002)	4.55 (±0.14)

Table S9: Prediction accuracy of the investigated neural networks. The numbers represent mean and standard deviation for N=3 independent neural network runs.

**Prediction error**	**Mean absolute error/ %**	**Accurate bin / %**	**1 bin off / %**	**2 bins off / %**	**3 bins off / %**
**GTNN2D**	16.7 (±0.13)	48.4 (±0.7)	36.0 (±0.9)	12.4 (±1.3)	3.1 (±0.5)
**GNN2D**	16.4 (±0.2)	46.5 (±1.0)	39.4 (±0.5)	11.8 (±0.5)	2.7 (±0.5)
**GTNN3D**	16.4 (±0.24)	49.0 (±1.8)	37.2 (±2.8)	11.1 (±1.5)	2.3 (±0.0)
**GNN3D**	16.2 (±0.14)	46.3 (±0.5)	40.4 (±1.5)	11.6 (±1.6)	1.9 (±0.5)
**GTNN2DQM**	**16.1 (±0.02)**	**49.3 (±0.5)**	**36.8 (±1.1)**	**11.2 (±1.0)**	**1.9 (±0.5)**
**GNN2DQM**	16.3 (±0.04)	46.9 (±1.4)	40.0 (±0.5)	10.3 (±1.7)	3.0 (±1.1)
**GTNN3DQM**	16.2 (± 0.16)	47.1 (±0.7)	38.3 (±0.8)	11.4 (±0.5)	2.3 (±0.9)
**GNN3DQM**	16.2 (±0.14)	46.1 (±2.7)	39.4 (±2.6)	12.4 (±1.4)	1.9 (±1.1)
**ECFP4NN**	18.2 (±0.05)	46.5 (±1.5)	36.0 (±1.7)	13.1 (±0.5)	1.9 (±0.5)

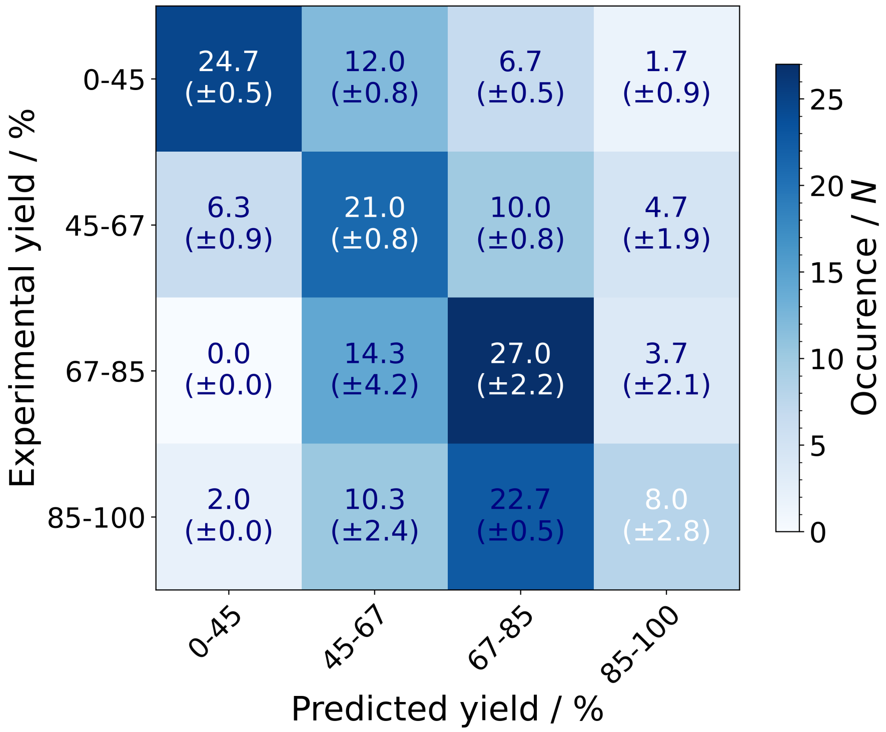

Figure S16: Performance of reaction yield prediction on the literature data set. Confusion matrix visualizing the accuracy of the best-performing neural network (GTNN3DQM) for reaction yields, divided into four equally sized bins.

SI9.3 Different thresholds for binary reaction outcome prediction

Binary reaction outcome prediction was investigated for different reaction yield thresholds (*i.e.*, >1%, >5%, >10%, and >20%) to enable tailored applications to the specific needs of different medicinal chemistry projects. Table S10 illustrates the performance of GTNN3D for the four different thresholds. Figure S17 illustrates the corresponding cofusion matrices thereof.

Table S10: Model performance of GTNN3D for binary reaction outcome prediction with different thresholds at >1%, >5%, >10%, and >20%. Five metrics are shown for each of the model to quantify model performance, *i.e.*, area under receiver operating characteristic curve (AUC), *F*-score, predictive positive value (PPV), true positive rate (TPR), and absolute accuracy. The numbers represent mean and standard deviation for N=3 independent neural network runs.

**Binary threshold**	**AUC** / %	***F*-score** / %	**PPV** / %	**TPR** / %	**Absolute accuracy** / %
**>1%**	94.5 (±0.2)	82.9 (±0.6)	80.5 (±0.6)	85.4 (±0.5)	91.9 (±0.3)
**>5%**	94.5 (±0.2)	84.2 (±0.4)	82.4 (±0.3)	86.1 (±0.6)	93.3 (±0.2)
**>10%**	95.6 (±0.3)	81.9 (±0.6)	80.1 (±0.7)	83.6 (±0.6)	92.9 (±0.3)
**>20%**	94.4 (±0.2)	82.9 (±0.4)	81.1 (±0.3)	84.9 (±0.9)	90.7 (±0.3)

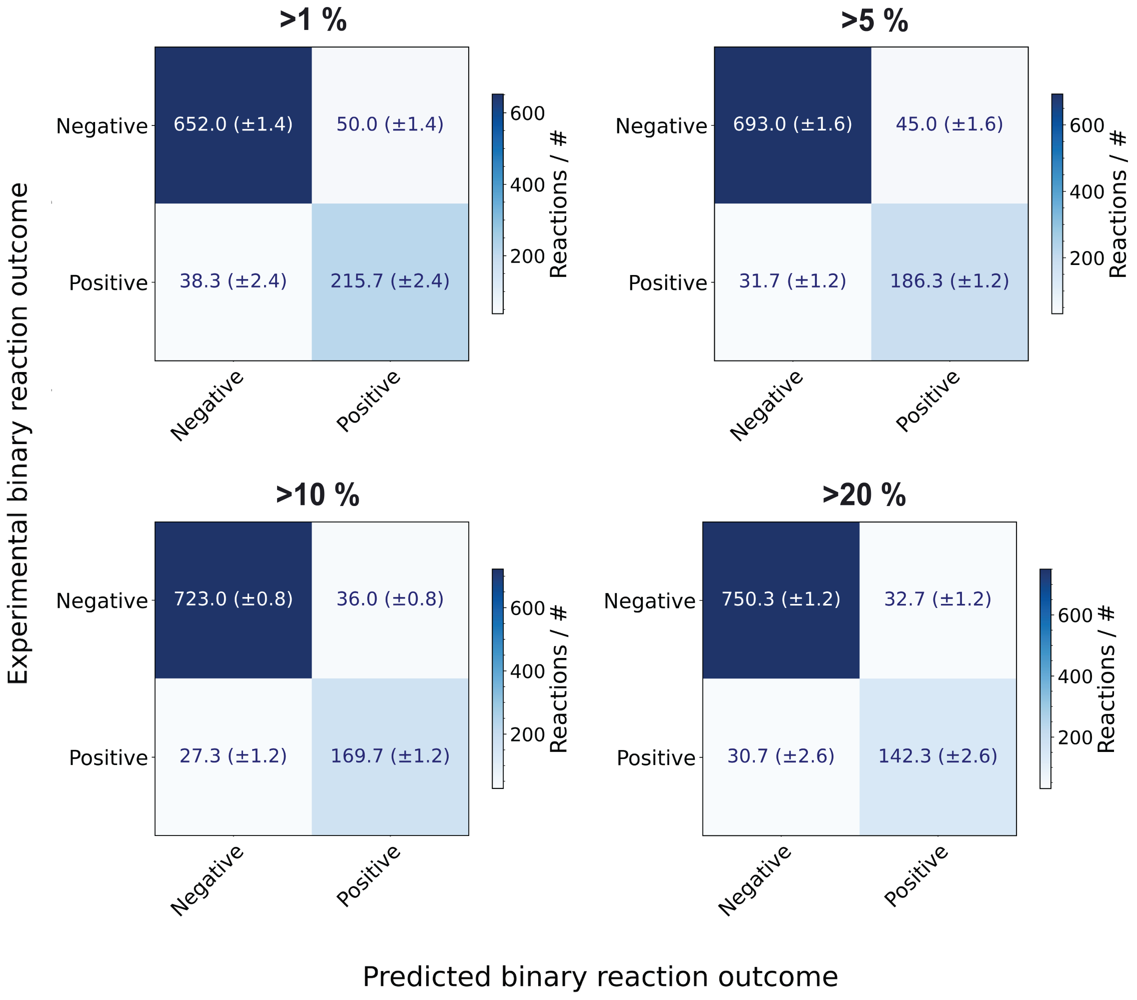

Figure S17: Model performance of GTNN3D for binary reaction outcome prediction with different thresholds at >1%, >5%, >10%, and >20%. Confusion matrix visualizing the accuracy for each thresholds.

SI10 Decision tree algorithms using reaction fingerprints

Fingerprint-based reaction representations in combination with classical machine learning algorithms (*e.g.* support vector machines, ridge regression, gradient boosting, or random forest) have shown applications in predicting reaction outcomes and reaction yields. [57] Here, we compare the results achieved through binary reactions fingerprints using two popular decision tree algorithms, gradient boosting and extreme gradient boosting (XGBoost). While both decision tree algorithms achieve comparable results for three of the four investigated tasks, they are outperformed by the best preforming graph neural network (Table S11) for all the investigated reaction tasks. Binary reaction fingerprints are composed by a one-hot encoding of the reaction conditions (*i.e.* catalyst, reagent, ligand, solvent) and a structure-based fingerprint of the substrate (*e.g.* ECFP4) (Figure S18).

The two decision tree algorithms were optimized using the following hyperparameters for screening:

- **XGBoost**: The XGBoost algorithm (XGBoost Python Package version 1.6.2 [58]) was optimized by fine-tuning the following hyperparameters: n_estimators=[1, 2, 5, 10, 20, 50, 100, 200], reg_lambda=[0.01, 0.05, 0.1, 0.5, 1], eta=[0.01, 0.05, 0.1, 0.5, 1], gamma=[0.01, 0.05, 0.1, 0.5, 1], and max_depth=[1, 2, 4, 6, 8, 10, 12, 14, 16].
- **Gradient boosting**: The gradient boosting algorithm (GradientBoostingClassifier and GradientBoostingRegressor by Sklearn version 0.23.2 [59]) was optimized by fine-tuning the following hyperparameters: n_estimators=[1, 2, 5, 10, 20, 50, 100, 200], learning_rate=[0.01, 0.05, 0.1, 0.5, 1], and max_depth=[1, 2, 4, 6, 8, 10, 12, 14, 16].

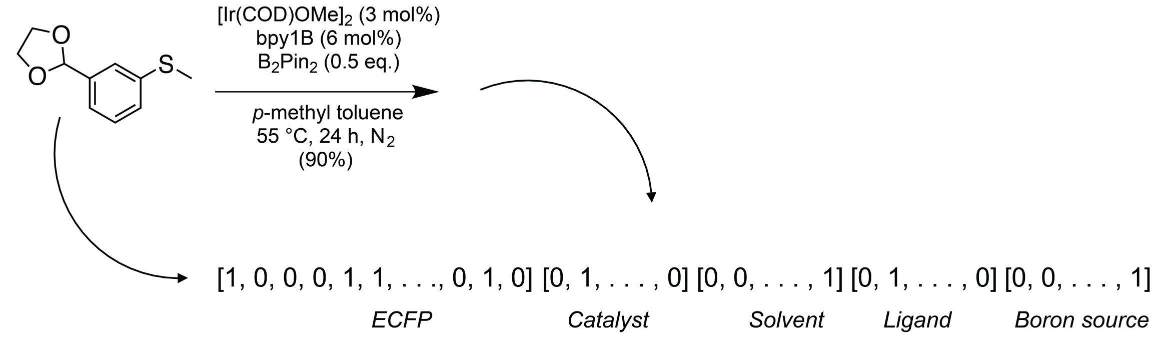

Figure S18: Illustration of binary fingerprint representations for an exemplary borylation reaction with four one-hot encoded reaction conditions (*i.e.* catalyst, reagent, ligand, solvent) and a structure-based fingerprint of the substrate.

Table S11: Model performance of the best graph neural network in comparison to the two decision tree algorithms, gradient boosting and extreme gradient boosting (XGBoost) for predicting binary reaction outcomes and reaction yields. Mean absolute errors (MAEs) were used to quantify reaction yield predictions. Balanced accuracy (AUC, area under receiver operating characteristic curve) was used to quantify binary reaction outcome predictions. The standard deviation is calculated through the results of three different hyperparameter initializations. Since the XGBoost algorithm is deterministic and uses its random state only for sub-sampling and not for initialization, the standard deviations are much lower and in all our cases even equal to zero. The numbers represent mean and standard deviation for N=3 independent neural network runs.

	**Reaction yield (literature), MAE / %**	**Reaction yield (experimental), MAE / %**	**Binary reaction outcome (experimental, random split), balanced accuracy / %**	**Binary reaction outcome (experimental, substrate split), balanced accuracy / %**
**Gradient boosting**	16.50 (±0.07)	5.56 (±0.03)	90.86 (±0.0)	52 (±4)
**XGBoost**	16.18 (±0.0)	5.32 (±0.0)	90.16 (±0.0)	44 (±0)
**Best graph neural network**	**16.11 (±0.02)**	**4.23 (±0.08)**	**91.8 (±0.9)**	**67 (±2)**

SI11 Borylation scale-ups

SI11.1 Reagent and purification information

Reactions were set up and conducted in nitrogen-filled gloveboxes from mbraun (Garching, DE) and LC Technologies (Salisbury, US). All chemicals were purchased from Sigma Aldrich (St. Louis, US), AstaTech (Bristol, US), Combi-Blocks (San Diego, US), TRC (Toronto, CA), Thermo Scientific (Waltham, US) or obtained from the Roche compound library and used as received. All solids were dosed using a CHRONECT Quantos from Axel Semrau GmbH & Co. KG (Spockhövel, DE) coupled with an XPE206 balance from Mettler Toledo (Greifensee, CH). Anhydrous solvents were purchased from Sigma Aldrich, stored in the glovebox and added to the reaction vials using pipettes from Eppendorf (Hamburg, DE). The vials were heated on a Junior benchtop solution from Unchained Labs (Pleasanton, US) and the reaction mixture was stirred by VP 721F-1 Parylene Encapsulated Stainless Steel Stir Discs from V&P Scientific Inc. (San Diego, US). Purification by flash column chromatography was performed using SiliaSep Premium Flash Cartridges from Silicycle (Quebec, CA) on a Combi Flash Rf from Teledyne ISCO (Nebraska, US). Eluent solvents, gradients and cartridge sizes for flash chromatography are described for each experiment.

SI11.2 Analytical information

All compounds were characterized by nuclear magnetic resonance (NMR) spectroscopy and (flow injection analysis (FIA)) high-resolution mass spectrometry (HRMS) or gas-chromatography mass spectrometry (GCMS). NMR spectra were recorded on a Bruker Avance III, 600 MHz spectrometer equipped with a 5 mm TCI, Z-gradient CryoProbe. NMR data are reported as follows: chemical shift in reference to the residual solvent peak (δ ppm), multiplicity (s = singlet, d = doublet, br d = broad doublet, dd = doublet of doublet, br dd = broad doublet of doublet, t = triplet, br t = broad triplet, m = multiplet), coupling constant (Hz), and integration. ^{1}H NMR residual solvent peaks in respective deuterated solvents for $CHCl_3$ at 7.26 ppm and DMSO at 2.50 ppm. ^{13}C NMR residual solvent peaks in respective deuterated solvents for $CHCl_3$ at 77.16 ppm and DMSO at 39.52 ppm.

LC-MS high-resolution spectra were recorded with an Agilent LC system consisting of Agilent 1290 high-pressure gradient system, and an Agilent 6545 QTOF. The separation was achieved on a Zorbax Eclipse Plus C18 1.7 µm 2.1 x 50 mm column (P/N 959731-902) at 55 °C; A: 0.01% HCOOH in H_2O; B: MeCN at flow 0.8 mL/min. Gradient: 0 min 5% B, 0.3 min 5% B, 4.5 min 99% B, 5 min 99% B. The injection volume was 2 µL. Ionization was performed in an Agilent Multimode source. The mass spectrometer was run in "2 GHz extended dynamic range" mode, resulting in a resolution of about 20 000 at m/z = 922. Mass accuracy was ensured by internal drift correction. GC-MS spectra were recorded on an Agilent 5975B single quadrupole mass spectrometer. Separation was achieved on an Agilent 7890A using a HP-1ms column (15 m ID: 250 µm and 0.25 µm film) with He as carrier gas. Sample introduction was done via a Split injector at 270°C. After 0.5 min at a constant temperature, the temperature was ramped from 100 °C or 45 °C to 320 °C with 35 °C/min. The mass spectrometer was operated in EI (electron ionization) mode at 70 eV. FIA-HRMS spectra were recorded with an Agilent LC system consisting of an Agilent 1290 high-pressure gradient system, and an Agilent 6540 QTOF. No separation was intended and the injected sample was flushed directly into the Agilent Jetstream source. The mass spectrometer was run in "2 GHz extended dynamic range" mode, resulting in a resolution of about 20 000 at m/z 922. Mass accuracy was ensured by internal drift correction.

SI11.3 Experimental procedures and analytical data

Ethyl 4-[13-chloro-6-(4,4,5,5tetramethyl-1,3,2-dioxaborolan-2-yl)-4-azatricyclo[9.4.0.-03,8]pentadeca-1(11),3(8),4,6,12,14-hexaen-2-ylidene]piperidine-1-carboxylate (1a):

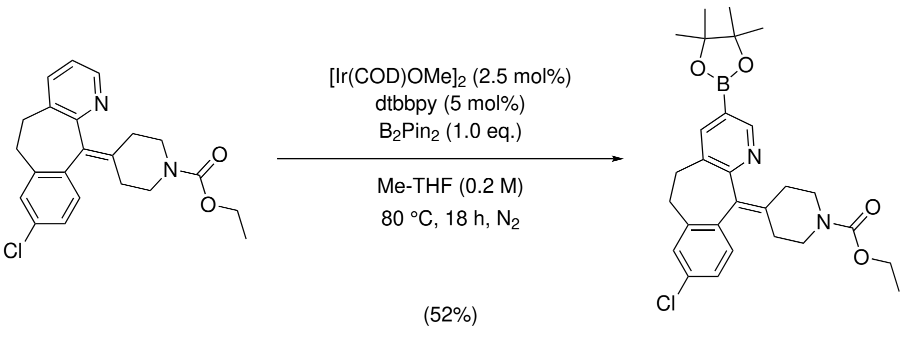

Figure S19: Monoborylation of Loratadine (**1**).

In an N_2-filled glovebox, ethyl 4-(13-chloro-4-azatricyclo[9.4.0.03,8]pentadeca-1(11),3(8),4,6,12,14-hexaen-2-ylidene)piperidine-1-carboxylate (**1**, 31.66 mg, 78.55 μmol, 1.00 eq.), bis(pinacolato)diboron (**3**, 19.95 mg, 78.55 μmol, 1.00 eq), 4,4'-dimethyl-2,2'-bipyridine (**7**, 723.63 ug, 3.93 μmol, 0.05 eq.) and bis(1,5-cyclooctadiene)-dimethoxydiiridium (**2**, 1.3 mg, 1.96 μmol, 0.025 eq.) were dosed by a solid handler. Addition of 2-methyl-THF (**11**, 398 μL) dissolved all components to give a reaction concentration of 0.2 M. The reaction was stirred at 80 °C for 18 h. The crude material was purified using silica gel column chromatography (4 g) using a MeOH gradient (0%-5%) in DCM. Evaporation of solvents gave the title compound 4-[13-chloro-6-(4,4,5,5-tetramethyl-1,3,2-dioxaborolan-2-yl)-4-azatricyclo[9.4.0.03,8]pentadeca-1(15),3,5,7,11,13-hexaen-2-ylidene]piperidine-1-carboxylic acid ethyl ester (**1a**, 23.0 mg, 52%) as a white solid.

^{1}H NMR (600 MHz, CDCl$_3$) δ (ppm) 8.78 - 8.69 (m, 1H), 7.87 - 7.77 (m, 1H), 7.12 (s, 3H), 4.25 - 4.01 (m, 3H), 3.88 - 3.75 (m, 2H), 3.36 (s, 1H), 3.43 - 3.28 (m, 1H), 3.14 - 2.99 (m, 2H), 2.82 (s, 1H), 2.89 - 2.73 (m, 1H), 2.52 - 2.42 (m, 1H), 2.40 - 2.25 (m, 3H), 1.43 - 1.30 (m, 15H). **^{13}C NMR (151 MHz, CDCl$_3$)** δ (ppm) 155.47, 152.51, 139.52, 137.67, 132.94, 132.57, 130.63, 129.05, 126.15, 84.17, 75.02, 61.33, 44.76, 31.78, 31.21, 24.86, 24.81, 14.68. **FIA-HRMS** $C_{28}H_{34}BClN_2O_4$; calc. for $(M+H^+)$: 509.2378, found: 509.2410.

Ethyl 4-[13-chloro-6,14-bis(4,4,5,5-tetramethyl-1,3,2-dioxaborolan-2-yl)-4-azatricyclo[9.4.0.03,8]pentadeca-1(11),3(8),4,6,12,14-hexaen-2-ylidene]piperidine-1-carboxylate (1b):

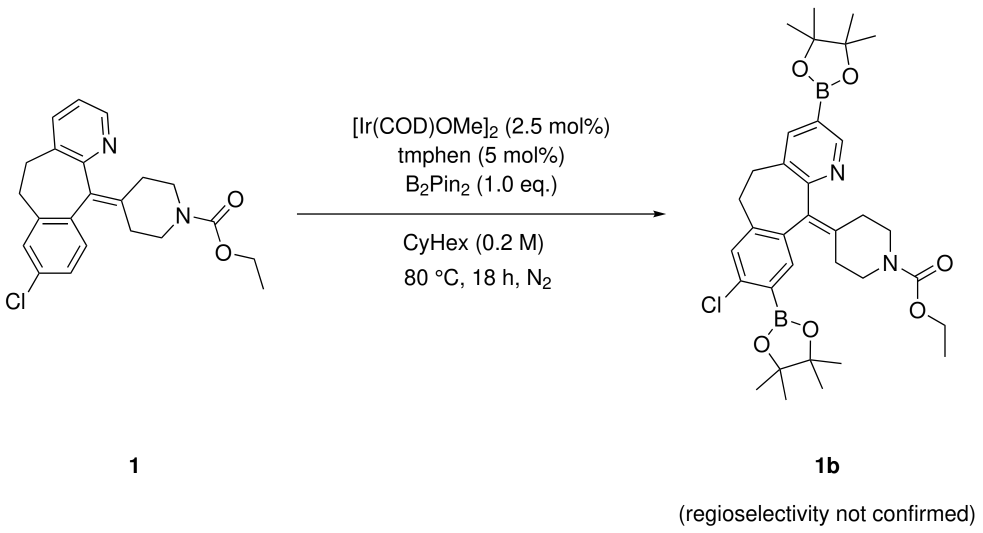

Figure S20: Diborylation of Loratadine (**1**).

In an N_2-filled glovebox, ethyl 4-(13-chloro-4-azatricyclo[9.4.0.03,8]pentadeca-1(11),3(8),4,6,12,14-hexaen-2-ylidene)piperidine-1-carboxylate (**1**, 500 mg, 1.28 mmol, 1.00 eq.), bis(pinacolato)diboron (**3**, 325 mg, 1.28 mmol, 1.00 eq.), 3,4,7,8-tetramethyl-1,10-phenanthroline (**9**, 15.1 mg, 64.0 μmol, 0.05 eq.) and bis(1,5-cyclooctadiene)dimethoxydiiridium (**2**, 21.2 mg, 32.0 mmol, 0.025 eq.) were dosed by a solid handler. Addition of cyclohexane (**10**, 6.39 mL) dissolved all components to give a reaction concentration of 0.2 M. The reaction was stirred at 80 °C for 18 h. The crude material was purified using silica gel column chromatography (40 g) using an EtOAc/EtOH (3:1) gradient (10%-30%) in heptane. Evaporation of solvents gave the title compound ethyl 4-[13-chloro-6,14-bis(4,4,5,5-tetramethyl-1,3,2-dioxaborolan-2-yl)-4-azatricyclo[9.4.0.03,8]pentadeca-1(11),3(8),4,6,12,14-hexaen-2-ylidene]piperidine-1-carboxylate (**1b**, 51.0 mg, 6%), which could only be characterized by HRMS. For confirmation of the regioselectivity, **1b** was transformed into **1c**.

FIA-HRMS $C_{34}H_{45}B_2ClN_2O_6$; calc. for $(M+H^+)$: 635.3231, found: 635.3458.

Ethyl 4-(13-chloro-6,14-dihydroxy-4-azatricyclo[9.4.0.03,8]pentadeca-1(11),3(8),4,6,12,14-hexaen-2-ylidene)piperidine-1-carboxylate (1c):

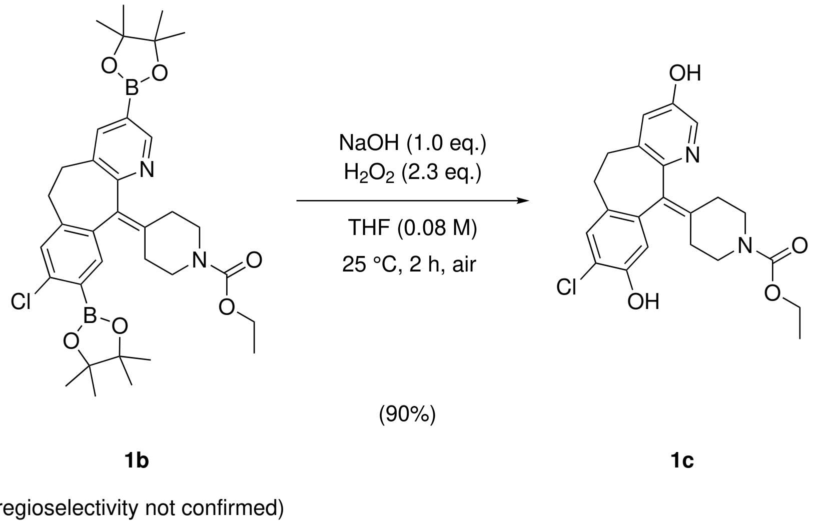

Figure S21: Hydroxylation of di-borylated Loratadine (**1b**).

Ethyl 4-[13-chloro-6,14-bis(4,4,5,5-tetramethyl-1,3,2-dioxaborolan-2-yl)-4-azatricyclo[9.4.0.03,8]pentadeca-1(11),3-(8),4,6,12,14-hexaen-2-ylidene]piperidine-1-carboxylate (**1b**, 51.0 mg, 0.08 mmol, 1.00 eq.) was dissolved in THF (**64**, 1.0 mL) to give a reaction concentration of 0.08 M. Next, NaOH (3.20 mg, 0.08 mmol, 1.00 eq.) and H_2O_2 (5.50 mL, 6.25 mg, 0.18 mmol, 2.30 eq.) were added to the reaction mixture, which was then stirred at 25 °C for 2 h. The reaction was worked up with H_2O_2 (10 mL) and extracted with EtOAc (3 x 10 mL). Combined organic phases were washed with brine and dried over Na_2SO_4. Evaporation of solvents gave the title compound Ethyl 4-(13-chloro-6,14-dihydroxy-4-azatricyclo[9.4.0.03,8]pentadeca-1(11),3(8),4,6,12,14-hexaen-2-ylidene)piperidine-1-carboxylate (**1c**, 30.0 mg, 90%) as a white solid.

^{1}H NMR (600 MHz, DMSO) δ (ppm) 9.93 (s, 1H), 9.73 (s, 1H), 7.88 (d, J = 2.7 Hz, 1H), 7.15 (s, 1H), 6.91 (d, J = 2.7 Hz, 1H), 6.64 (s, 1H), 4.02 - 4.05 (m, 2H), 3.56 - 3.61 (m, 2H), 3.12 - 3.18 (m, 4H), 2.68 - 2.72 (m, 1H), 2.63 - 2.67 (m, 1H), 2.25 - 2.30 (m, 1H), 2.20 - 2.24 (m, 2H), 2.13 - 2.16 (m, 1H), 1.17 (t, J = 7.1 Hz, 3H). **^{13}C NMR (151 MHz, DMSO)** δ (ppm) 155.03, 152.89, 151.08, 148.02, 139.90, 135.66, 134.78, 134.49, 134.18, 130.39, 130.02, 123.76, 118.33, 116.88, 61.15, 31.77, 30.33, 15.11. **HRMS** $C_{22}H_{23}ClN_2O_4$; calc. for (M+H$^+$): 415.1424, found: 415.1420.

4-Hydroxy-3-(3-oxo-1-phenyl-butyl)-7-(4,4,5,5-tetramethyl-1,3,2-dioxaborolan-2-yl)chromen-2-one (25a):

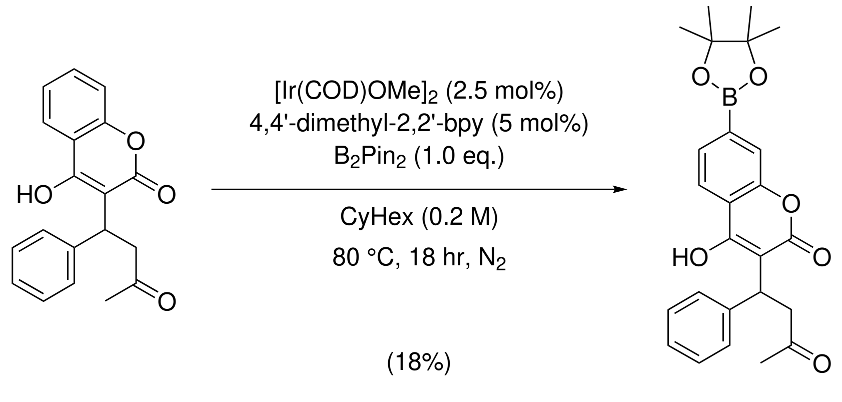

25 **25a**

Figure S22: Borylation of Warfarin (**25**).

In an N_2-filled glovebox, 4-Hydroxy-3-(3-oxo-1-phenylbutyl)- 2H-chromen-2-one (**25**, 247 mg, 0.8 mmol, 1.00 eq.), bis(pinacolato)diboron (**3**, 203 mg, 0.8 mmol, 1.00 eq.), 4,4′-dimethyl-2,2′-bipyridine (**7**, 7.4 mg, 0.04 mmol, 0.05 eq.) and bis(1,5-cyclooctadiene)dimethoxydiiridium (**2**, 13.2 mg, 0.02 mmol, 0.025 eq.) were dosed by a solid handler. Addition of cyclohexane (**10**, 6.39 mL) dissolved all components to give a reaction concentration of 0.2 M. The reaction was stirred at 80 °C for 18 h. The crude material was purified using silica gel column chromatography (12 g) using an EtOAc/EtOH (3:1) gradient (0%-25%) in heptane, followed by another silica gel chromatography (4 g) using a EtOAc/EtOH (3:1) gradient (0%-10%) in heptane. Evaporation of solvents gave the title compound 4-Hydroxy-3-(3-oxo-1-phenyl-butyl)-7-(4,4,5,5-tetramethyl-1,3,2-dioxaborolan-2-yl)chromen-2-one (**25a**, 48.0 mg, 18%) as a white solid.

^{1}H NMR (600 MHz, DMSO) δ (ppm) 8.43 - 8.33 (m, 3H), 7.83 - 7.68 (m, 4H), 7.38 (s, 1H), 7.29 - 7.11 (m, 7H), 3.99 (br dd, J = 6.7, 11.2 Hz, 1H), 2.38 - 2.25 (m, 2H), 2.22 - 2.04 (m, 1H), 1.90 (br t, J = 12.1 Hz, 1H), 1.69 - 1.55 (m, 4H), 1.42 - 1.13 (m, 2H). **^{13}C NMR (151 MHz, DMSO)** δ (ppm) 158.31, 151.58, 143.69, 128.13, 126.96, 125.83, 122.20, 121.16, 117.82, 104.47, 99.67, 84.18, 42.63, 27.07. **FIA-HRMS** $C_{25}H_{27}BO_6$; calc. for $(M+H^+)$: 435.1979, found: 435.1824.

2-cyclopropyl-7-methyl-13-(4,4,5,5-tetramethyl-1,3,2-dioxaborolan-2-yl)-2,4,9,15-tetrazatricyclo[9.4.0.03,8]-pentadeca-1(15),3(8),4,6,11,13-hexaen-10-one (**29a**):

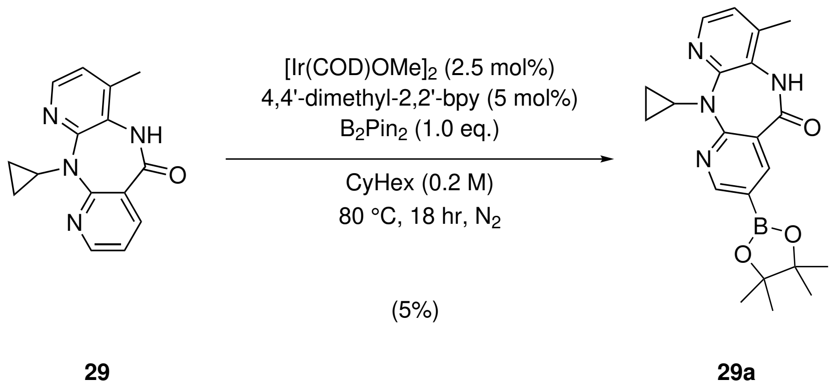

Figure S23: Borylation of Nevirapine (**29**).

In an N_2-filled glovebox, 11-cyclopropyl-4-methyl-5,11-dihydro-6H- dipyrido[3,2-b:2′,3′-e][1,4]diazepin-6-one (**29**, 26.6 mg, 0.1 mmol, 1.00 eq.), bis(pinacolato)diboron (**3**, 253 mg, 1.0 mmol, 1.00 eq.), 4,4′-dimethyl-2,2′-bipyridine (**7**, 9.3 mg, 0.05 mol, 0.05 eq.) and bis(1,5-cyclooctadiene)dimethoxydiiridium (**2**, 16.5 mg, 0.025 mmol, 0.025 eq.) were dosed by a solid handler. Addition of cyclohexane (**10**, 0.5 mL) dissolved all components to give a reaction concentration of 0.2 M. The reaction was stirred at 80 °C for 18 h. The crude material was purified using silica gel column chromatography (4 g) using an EtOAc/EtOH (3:1) gradient (5%-25%) in heptane, followed by another silica gel chromatography (4 g) using a EtOAc/EtOH (3:1) gradient (0%-25%) in heptane. Evaporation of solvents gave the title compound 2-cyclopropyl-7-methyl-13-(4,4,5,5-tetramethyl-1,3,2-dioxaborolan-2-yl)-2,4,9,15-tetrazatricyclo[9.4.0.03,8]pentadeca-1(15),3(8),4,6,11,13-hexaen-10-one (**29a**, 9.0 mg, 5%) as a white solid.

^{1}H NMR (600 MHz, DMSO) δ (ppm) 9.89 (s, 1H), 8.66 (d, J = 2.0 Hz, 1H), 8.22 (d, J = 2.0 Hz, 1H), 8.10 (d, J = 4.8 Hz, 1H), 7.10 (dd, J = 4.8, 0.7 Hz, 1H), 3.63 (dt,J = 6.9, 3.3 Hz, 1H), 2.35 (s, 3H), 1.31 (d, J = 4.8 Hz, 10H), 1.08 (s, 1H), 0.92 (s, 2H), 0.30 - 0.44 (m, 2H). **^{13}C NMR (151 MHz, $CDCl_3$)** δ (ppm) 168.21, 162.26, 158.12, 153.51, 147.06, 144.34, 138.52, 124.66, 121.99, 119.07, 84.18, 29.78, 24.85, 24.83, 17.68. **LCMS** $C_{21}H_{25}BN_4O_3$; calc. for ($M+H^+$): 392.2, found: 392.2.

3-(2,5-dimethylpyrrol-1-yl)-1-methyl-5-(4,4,5,5-tetramethyl-1,3,2-dioxaborolan-2-yl)pyrazole (**37a**):

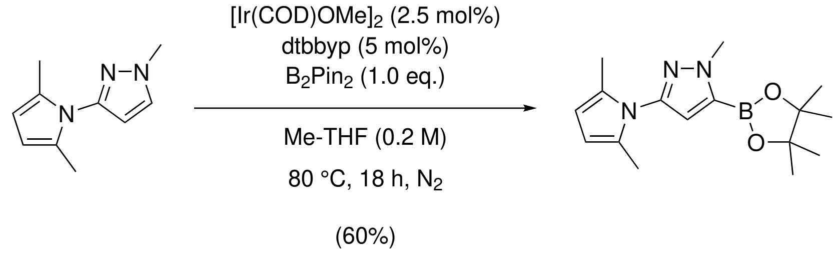

Figure S24: Monoborylation of **37**.

In an N_2-filled glovebox, ethyl 3-(2,5-dimethylpyrrol-1-yl)-1-methyl-pyrazole (**37**, 140.18 mg, 800 μmol, 1.000 eq), bis(pinacolato)diboron (**3**, 203.15 mg, 800 μmol, 1.000 eq), dtbbpy (**6**, 10.74 mg, 40.0 μmol, 0.05 eq) and bis(1,5-cyclooctadiene)dimethoxydiiridium (**2**, 13.26 mg, 20.0 μmol, 0.025 eq) were dosed by a solid handler. Addition of Me-THF (**11**, 4.0 mL) dissolved all components to give a reaction concentration of 0.2 M. The reaction was stirred at 80 °C for 18 h, followed by evaporation of the solvent. The crude material was purified by silica gel column chromatography (40 g) using a MeOH gradient (0%-5%) in DCM. Evaporation of solvents gave the title compound 3-(2,5-dimethylpyrrol-1-yl)-1-methyl-5-(4,4,5,5-tetramethyl-1,3,2-dioxaborolan-2-yl)pyrazole (**37a**, 146.00 mg, 60%) as an off-white solid.

^{1}H NMR (600 MHz, CDCl$_3$) δ (ppm) 6.57 (s, 1H), 5.85 (s, 2H), 4.09 (s, 3H), 2.10 (s, 6H), 1.38 (s, 12H). **^{13}C NMR (151 MHz, CDCl$_3$)** δ (ppm) 146.14, 129.33, 1124.55, 105.86, 84.44, 39.76, 24.88, 12.92. **GCMS** $C_{16}H_{24}BN_3O$; calc. for (M^{*+}): 301.2, found: 301.2.

4-[5-bromo-4-(4,4,5,5-tetramethyl-1,3,2-dioxaborolan-2-yl)-2-pyridyl]morpholine (38a):

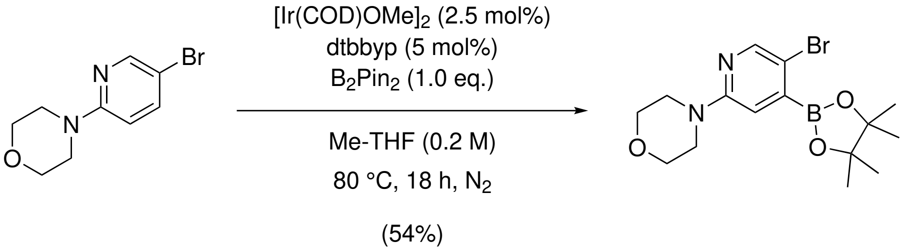

38 **38a**

Figure S25: Monoborylation of **38**.

In an N_2-filled glovebox, 4-(5-bromo-2-pyridyl)morpholine (**38**, 194.48 mg, 800 μmol, 1.00 eq.), bis(pinacolato)-diboron (**3**, 203.15 mg, 800 μmol, 1.00 eq.), dtbbpy (**6**, 10.74 mg, 40.0 μmol, 0.05 eq.) and bis(1,5-cyclooctadiene)-dimethoxydiiridium (**2**, 13.26 mg, 20.0 μmol, 0.025 eq.) were dosed by a solid handler. Addition of 2-methyl-THF (**11**, 4.0 mL) dissolved all components to give a reaction concentration of 0.2 M. The reaction was stirred at 80 °C for 18 h, followed by evaporation of the solvent. The crude material was purified by silica gel column chromatography (40 g) using a MeOH gradient (0%-5%) in DCM. Evaporation of solvents gave the title compound 4-[5-bromo-4-(4,4,5,5-tetramethyl-1,3,2-dioxaborolan-2-yl)-2-pyridyl]morpholine (**38a**, 161.00 mg, 54%) as a white solid.

^{1}H NMR (600 MHz, $CDCl_3$) δ (ppm) 8.25 (d, J = 0.6 Hz, 1H), 6.84 (s, 1H), 3.80 - 3.82 (m, 4H), 3.48 - 3.50 (m, 4H), 1.38 (s, 12H). **^{13}C NMR (151 MHz, $CDCl_3$)** δ (ppm) 157.59, 149.20, 113.51, 112.82, 84.84, 66.70, 45.59, 24.83. **GCMS** $C_{15}H_{22}BBrN_2O_3$; calc. for (M^{*+}): 368.1, found: 368.1.

6-bromo-1-[(4,4,5,5-tetramethyl-1,3,2-dioxaborolan-2-yl)methyl]-3-(trifluoromethyl)indazole (39a):

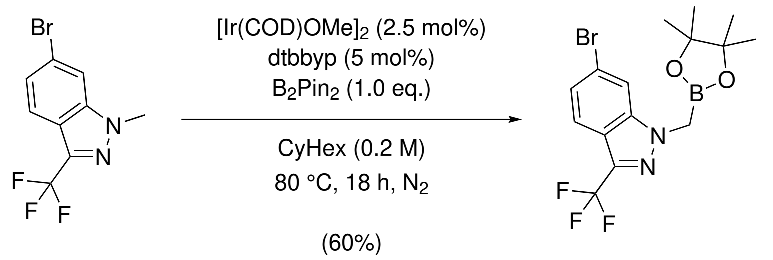

39 **39a**

Figure S26: Monoborylation of **39**.

In an N_2-filled glovebox, 6-bromo-1-methyl-3-(trifluoromethyl)indazole (**39**, 223.25 mg, 800 μmol, 1.00 eq), bis-(pinacolato)diboron (**3**, 203.15 mg, 800 μmol, 1.00 eq), dtbbpy (**6**, 10.74 mg, 40.0 μmol, 0.05 eq) and bis(1,5-cyclooctadiene)dimethoxydiiridium (**2**, 13.26 mg, 20.0 μmol, 0.025 eq) were dosed by a solid handler. Addition of cyclohexane (**10**, 4.0 mL) dissolved all components to give a reaction concentration of 0.2 M. The reaction was stirred at 80 °C for 18 h, followed by evaporation of the solvent. The crude material was purified by silica gel column chromatography (40 g) using a MeOH gradient (0%-5%) in DCM. Evaporation of solvents gave the title compound 6-bromo-1-[(4,4,5,5-tetramethyl-1,3,2-dioxaborolan-2-yl)methyl]-3-(trifluoromethyl)indazole (**39a**, 197.0 mg, 60%) as a light yellow solid.

^{1}H NMR (300 MHz, $CDCl_3$) δ (ppm) 7.68 (d, J = 8.6 Hz, 1H), 7.61 - 7.62 (m, 1H), 7.37 (dd, J = 1.6, 8.7 Hz, 1H), 4.11 (s, 2H), 1.31 (s, 12H). **^{13}C NMR (151 MHz, $CDCl_3$)** δ (ppm) 141.53, 125.99, 121.21, 113.11, 84.99, 24.72. **GCMS** $C_{15}H_{17}BBrF_3N_2O_2$; calc. for (M^{*+}): 404.1, found: 404.1.

[6-hydroxy-8-(4,4,5,5-tetramethyl-1,3,2-dioxaborolan-2-yl)-2-naphthyl]-morpholino-methanone (45a) and **[6-hydroxy-4-(4,4,5,5-tetramethyl-1,3,2-dioxaborolan-2-yl)-2-naphthyl]-morpholino-methanone (45b)**:

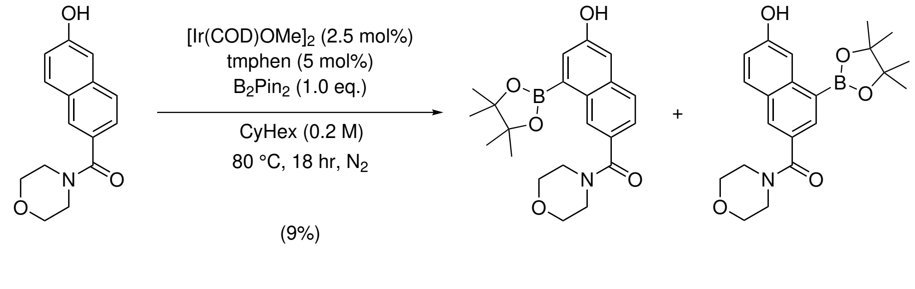

Figure S27: Monoborylation of **45**.

In an N_2-filled glovebox, (6-hydroxy-2-naphthyl)-morpholino-methanone (**45**, 25.7 mg, 0.1 mmol, 1.00 eq), bis-(pinacolato)diboron (**3**, 253 mg, 1.0 mmol, 1.00 eq.), tmphen (**6**, 11.82 mg, 0.05 mmol, 0.05 eq) and bis(1,5-cyclooctadiene)dimethoxydiiridium (**2**, 16.5 mg, 0.025 mmol, 0.025 eq.) were dosed by a solid handler. Addition of cyclohexane (**10**, 0.5 mL) dissolved all components to give a reaction concentration of 0.2 M. The reaction was stirred at 80 °C for 18 h, followed by evaporation of the solvent. The crude material was purified by silica gel column chromatography (4 g) using a MeOH gradient (0%-75%) in DCM. Evaporation of solvents gave the title compounds 6-hydroxy-8-(4,4,5,5-tetramethyl-1,3,2-dioxaborolan-2-yl)-2-naphthyl]-morpholino-methanone (**45a**) and -[6-hydroxy-4-(4,4,5,5-tetramethyl-1,3,2-dioxaborolan-2-yl)-2-naphthyl]-morpholino-methanone (**45b**) as an isomeric mixture (combined 3.4 mg, 9%).

^{1}H NMR (600 MHz, $CDCl_3$) δ (ppm) 8.72 - 8.75 (m, 1H), 8.07 - 8.10 (m, 1H), 8.05 (d, J = 1.9 Hz, 1H), 7.89 (d, J = 1.9 Hz, 1H), 7.72 (d, J = 2.5 Hz, 1H), 7.69 - 7.73 (m, 1H), 7.60 (s, 1 H), 7.46 - 7.50 (m, 1H), 7.15 - 7.19 (m, 1H), 7.09 - 7.14 (m, 1H), 5.54 - 6.30 (m, 1H), 3.70 - 3.89 (m, 8H), 1.40 (s, 12H). **^{13}C NMR (151 MHz, $CDCl_3$)** δ (ppm) 171.40, 153.93, 135.61, 134.55, 131.03, 130.75, 129.69, 128.26, 127.80, 127.50, 125.09, 113.17, 84.09, 83.98, 67.12, 66.96, 25.04, 25.00. **HRMS** $C_{21}H_{26}BNO_5$; calc. for (M+H$^+$): 384.1982, found: 384.1979.

***tert*-butyl (6-chloro-4-(4,4,5,5-tetramethyl-1,3,2-dioxaborolan-2-yl)-2,3-dihydro-1H-inden-2-yl)carbamate (64a)**:

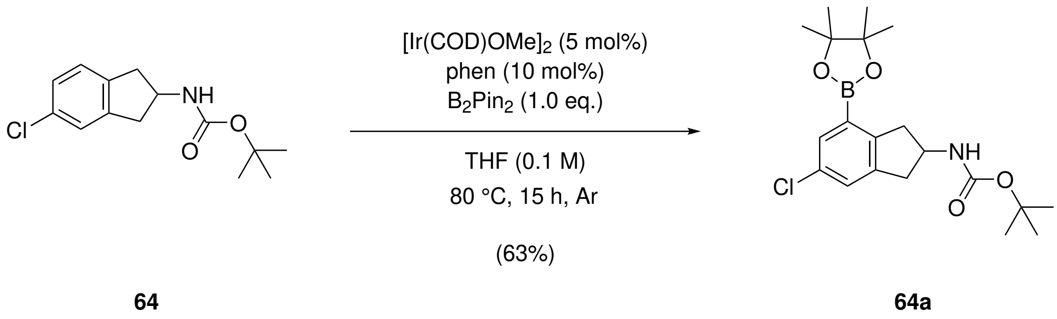

Figure S28: Monoborylation of **64**.

Under an Ar atmosphere, tert-butyl (5-chloro-2,3-dihydro-1H-inden-2-yl)carbamate (**64**, 2.50 mg, 9.34 mmol, 1.00 eq), bis(pinacolato)diboron (**3**, 2.42 g, 9.34 mmol, 1.00 eq.), phen (**8**, 221 mg, 0.93 mmol, 0.10 eq) and bis(1,5-cyclooctadiene)dimethoxydiiridium (**2**, 309 mg, 0.47 mmol, 0.05 eq.) were added to a vial. The addition of THF (**62**, 10 mL) dissolved all components to give a reaction concentration of 0.1 M. The reaction was stirred at 80 °C for 15 h, followed by evaporation of the solvent. The crude material was purified by silica gel column chromatography (20 g) using an EtOAc gradient (0%-30%) in heptane. Evaporation of solvents gave the title compound tert-butyl (6-chloro-4-(4,4,5,5-tetramethyl-1,3,2-dioxaborolan-2-yl)-2,3-dihydro-1H-inden-2-yl)carbamate (**64a**) as an off-white solid (2.30 g, 63%).

LCMS $C_{20}H_{29}BClNO_4$; calc. for (M-Boc+H^+): 293.1324, found: 294.2.

***tert*-butyl (6-chloro-4-hydroxy-2,3-dihydro-1H-inden-2-yl)carbamate (64b)**:

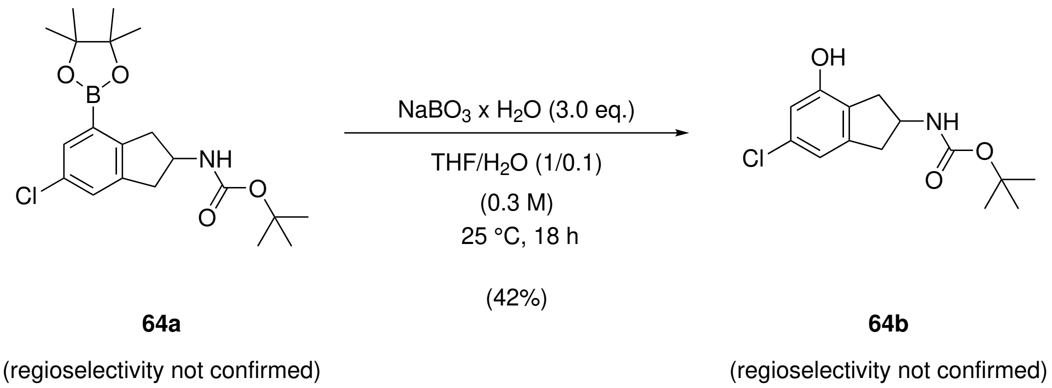

Figure S29: Conversion of **64a** to **64b**.

tert-butyl (6-chloro-4-(4,4,5,5-tetramethyl-1,3,2-dioxaborolan-2-yl)-2,3-dihydro-1H-inden-2-yl)carbamate (**64a**, 850.0 mg, 1.84 mmol, 1.00 eq.) was dissolved in THF (**62**, 5.56 mL) and H_2O (556 uL), followed by addition of sodium perborate monohydrate (549 mg, 5.51 mmol, 3.00 eq.). The reaction was stirred at 25 °C for 18 hours. The solvent evaporated and the residue was taken up in H_2O, followed by extraction with EtOAc to separate the two layers. The aqueous layer was extracted twice with EtOAc. The combined organic layers were washed with brine, dried over anhydrous sodium sulfate and evaporated to dryness. The crude material was purified by silica gel column chromatography (10 g) using an EtOAc gradient (0%-50%) in heptane. Evaporation of solvents gave the title compound *tert*-butyl (6-chloro-4-hydroxy-2,3-dihydro-1H-inden-2-yl)carbamate (**64b**) as an off-white solid (220 mg, 42%).

LCMS $C_{20}H_{29}BClNO_4$; calc. for (M-H^+): 282.1, found: 282.2.

2-((*tert*-butoxycarbonyl)amino)-6-chloro-2,3-dihydro-1H-inden-4-yl trifluoromethanesulfonate (64c):

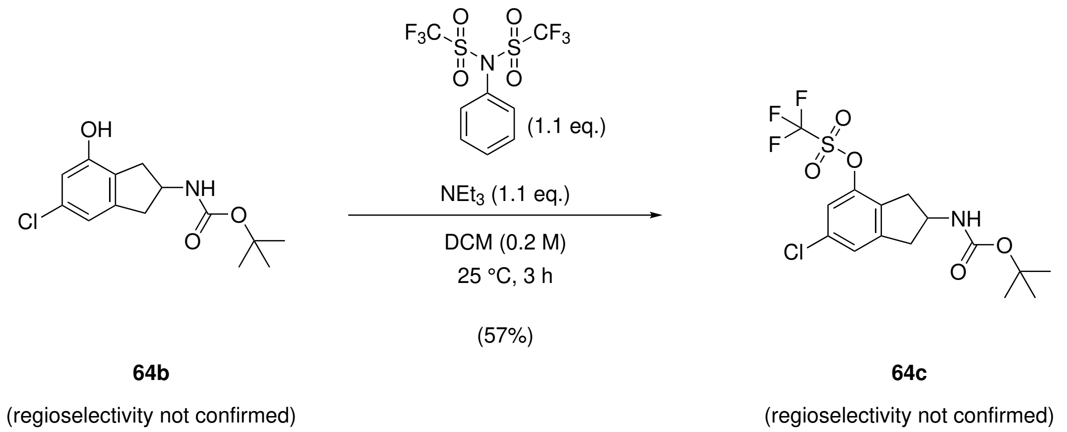

Figure S30: Conversion of **64b** to **64c**.

tert-butyl (6-chloro-4-hydroxy-2,3-dihydro-1H-inden-2-yl)carbamate (**64b**, 50.0 mg, 176 umol, 1.00 eq.) was dissolved in dry DCM (750 uL) and triethylamine (19.6 mg, 27 uL, 194 umol, 1.10 eq.) was added. To this stirring solution, 1,1,1-trifluoro-N-phenyl-N-((trifluoromethyl)sulfonyl)methanesulfonamide (69.2 mg, 194 umol, 1.10 eq.) was added. The reaction was stirred at 25 °C for three hours. The reaction was poured into EtOAc and the layers were separated. The aqueous layer was extracted twice with EtOAc. The combined organic layers were washed with brine, dried over anhydrous sodium sulfate and evaporated to dryness. The crude material was purified by silica gel column chromatography (4 g) using an EtOAc gradient (0%-50%) in heptane. Evaporation of solvents gave the title compound *tert*-butoxycarbonyl)amino)-6-chloro-2,3-dihydro-1H-inden-4-yl trifluoromethanesulfonate (**64c**) as a white solid (42.0 mg, 57%).

LCMS $C_{20}H_{29}BClNO_4$; calc. for (M-H$^+$): 414.0, found: 414.1.

***tert*-butyl (6-chloro-4-cyano-2,3-dihydro-1H-inden-2-yl)carbamate (64d):**

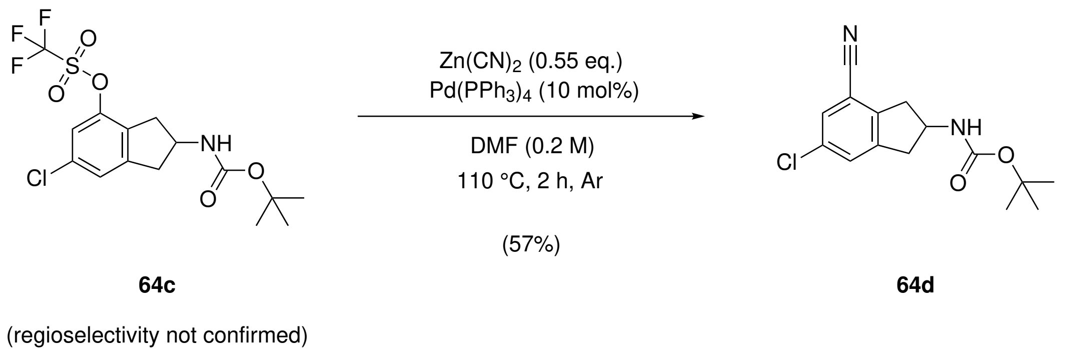

Figure S31: Conversion of **64c** to **64d**.

2-((*tert*-butoxycarbonyl)amino)-6-chloro-2,3-dihydro-1H-inden-4-yl trifluoromethanesulfonate (**64c**, 60.0 mg, 144 umol, 1.00 eq.), zinc cyanide (9.32 mg, 79.4 umol, 0.55 eq.) and tetrakis(triphenylphosphine) palladium (16.7 mg, 14.4 umol, 0.10 eq.) were dissolved in dry DMF (721 uL) and Argon was bubbled through the reaction for five minutes. The reaction was stirred at 110 °C for two hours. The reaction was poured into LiCl 10% and extracted with EtOAc. The layers were separated and the aqueous layer was extracted twice with EtOAc. The combined organic layers were washed with brine, dried over anhydrous sodium sulfate and evaporated to dryness. The crude material was purified by silica gel column chromatography (2 g) using a EtOAc gradient (0%-50%) in

heptane. Evaporation of solvents gave the title compound *tert*-butyl (6-chloro-4-cyano-2,3-dihydro-1H-inden-2-yl)carbamate (**64d**) as a white solid (24.0 mg, 57%).

^{1}H NMR (600 MHz, CDCl$_3$) δ (ppm) 7.44 - 7.45 (m, 1 H), 7.42 (d, J = 1.9 Hz, 1 H), 4.74 (s, 1 H), 4.54 (s, 1 H), 3.43 (dd, J = 17.1, 7.3 Hz, 1 H), 3.34 (dd, J = 16.6, 7.2 Hz, 1 H), 2.97 (dd, J = 17.1, 5.2 Hz, 1 H), 2.88 - 2.92 (m, 1 H), 1.46 (s, 9 H). **^{13}C NMR (151 MHz, CDCl$_3$)** δ (ppm) 155.2, 144.6, 110.3. **GCMS** $C_{15}H_{17}ClN_2O_2$; calc. for (M^{*+}): 292.1, found: 292.1.

SI12 NMR spectra

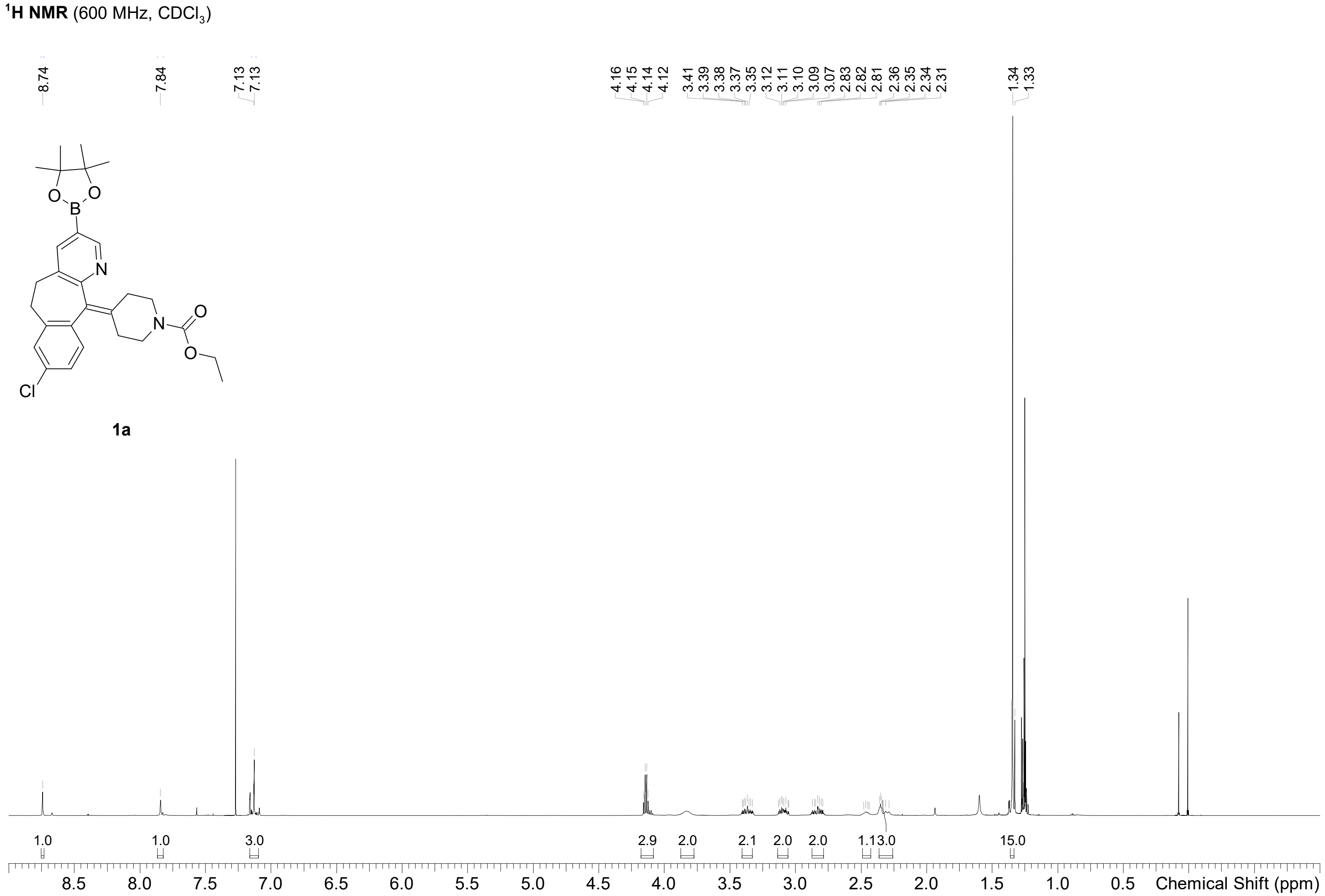

Figure S32: **1a**, ^{1}H-NMR spectra.

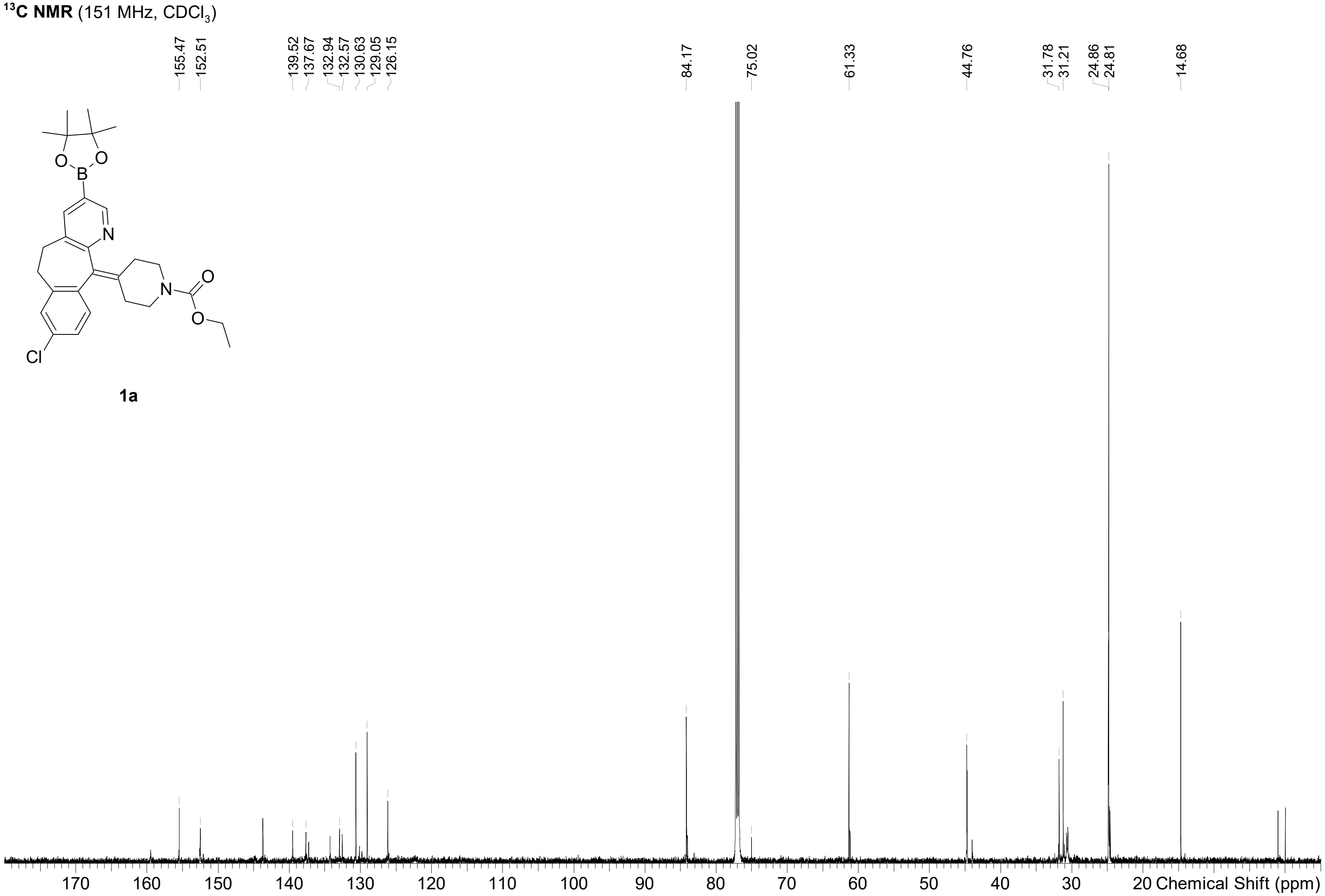

Figure S33: **1a**, ^{13}C-NMR spectra.

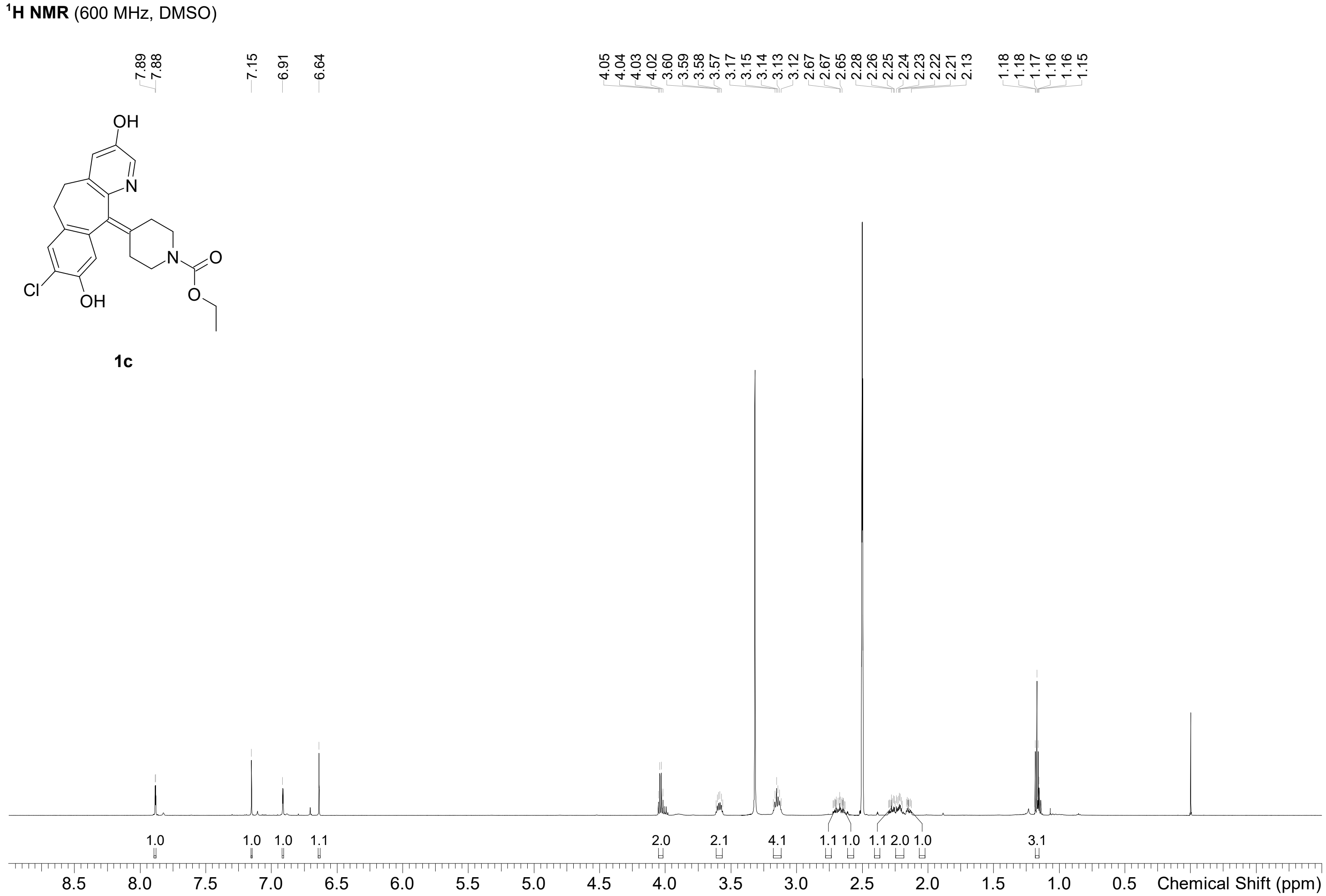

Figure S34: **1c**, ^{1}H-NMR spectra.

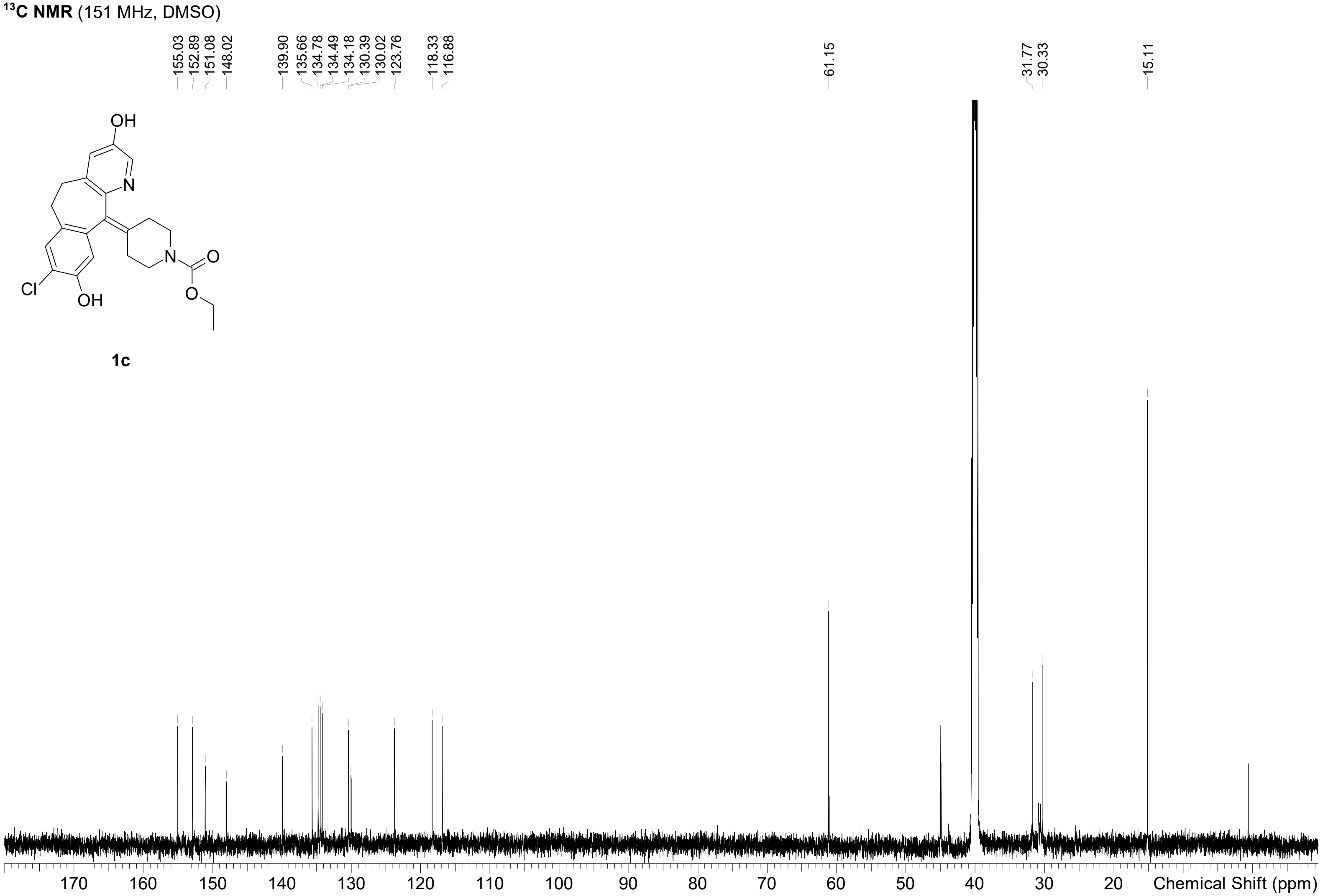

Figure S35: **1c**, ^{13}C-NMR spectra.

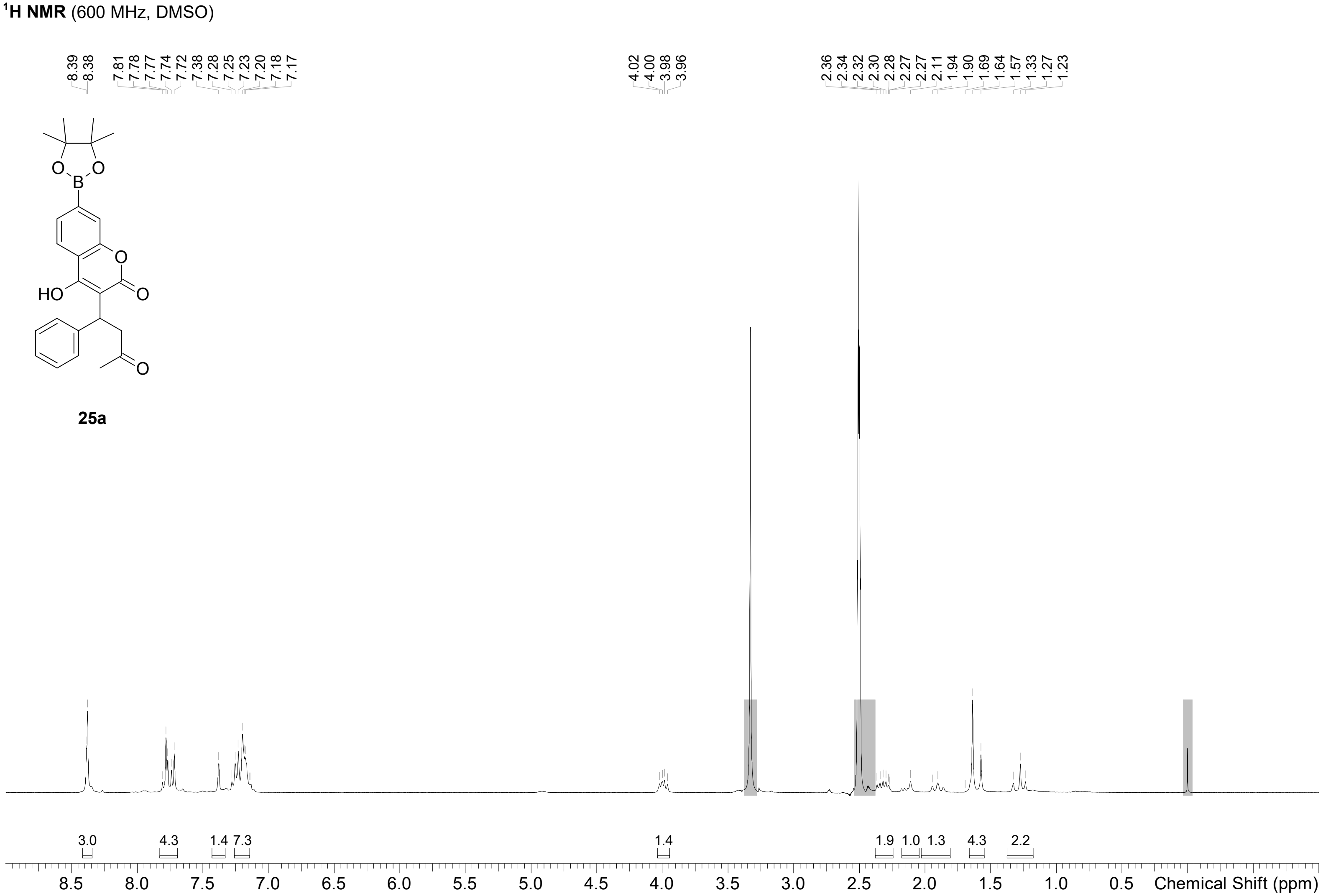

Figure S36: **25a**, ^{1}H-NMR spectra.

^{13}C NMR (151 MHz, DMSO)

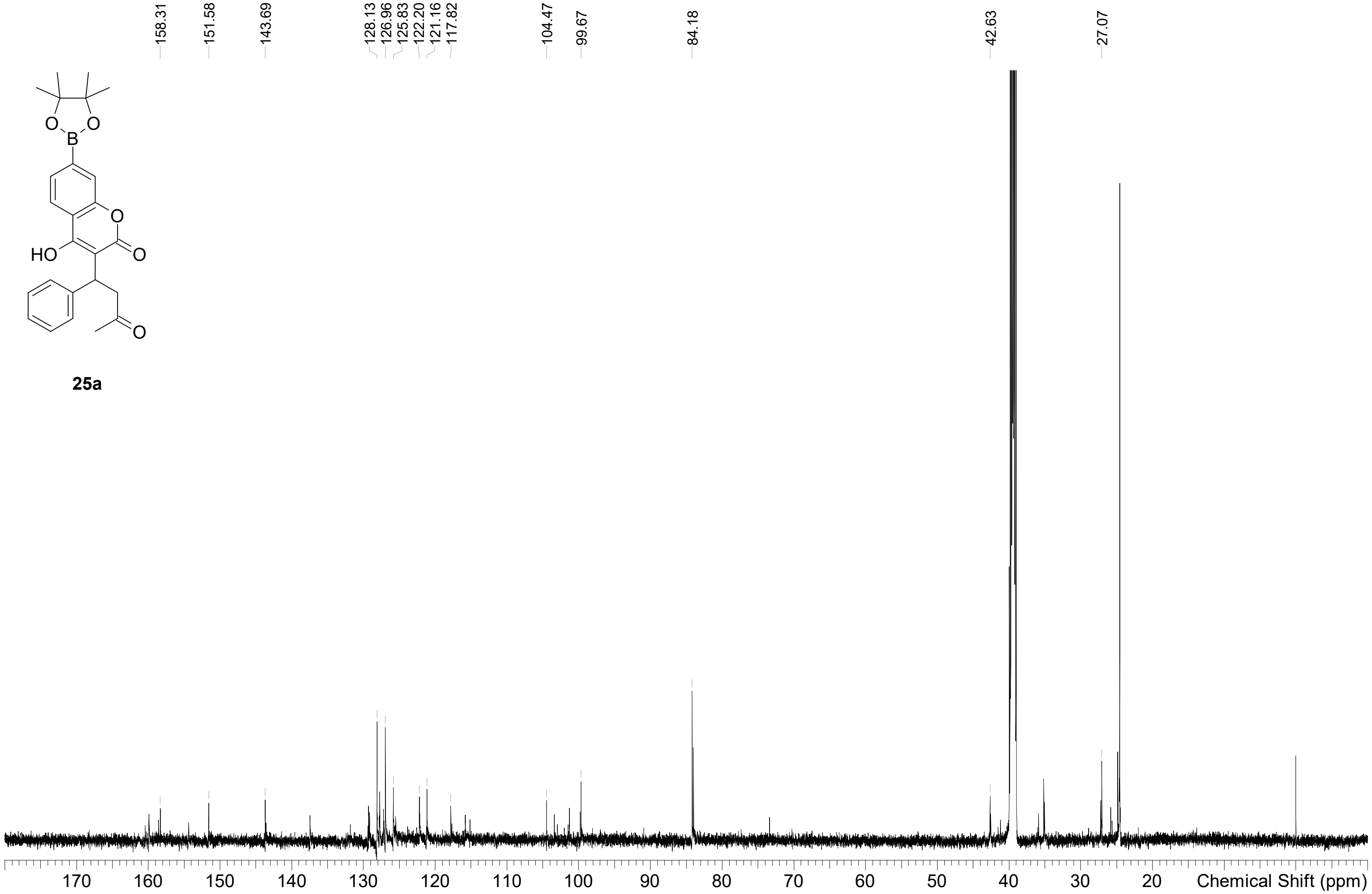

Figure S37: **25a**, ^{13}C-NMR spectra.

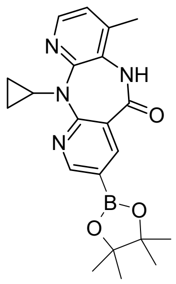

29a

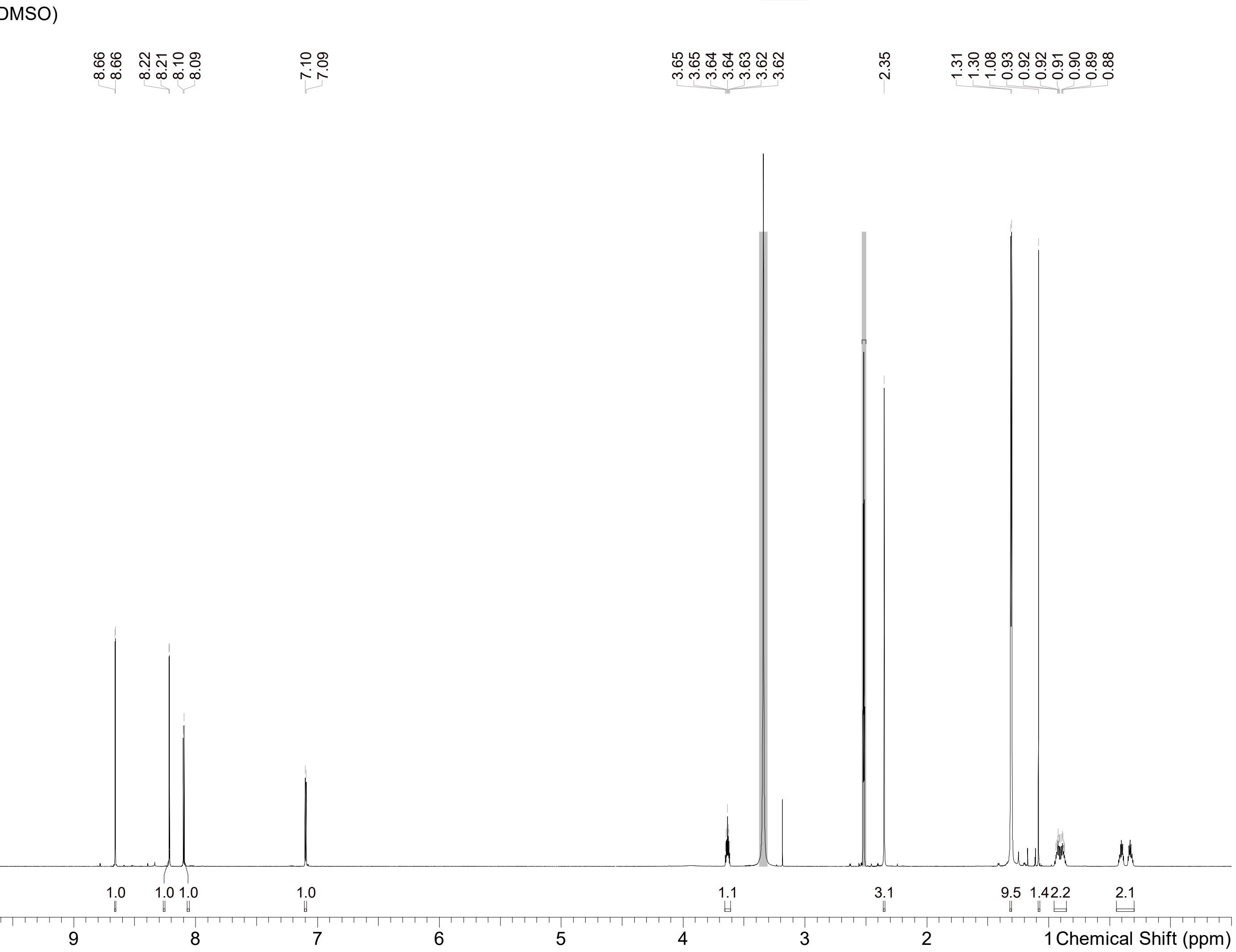

Figure S38: **29a**, ^{1}H-NMR spectra.

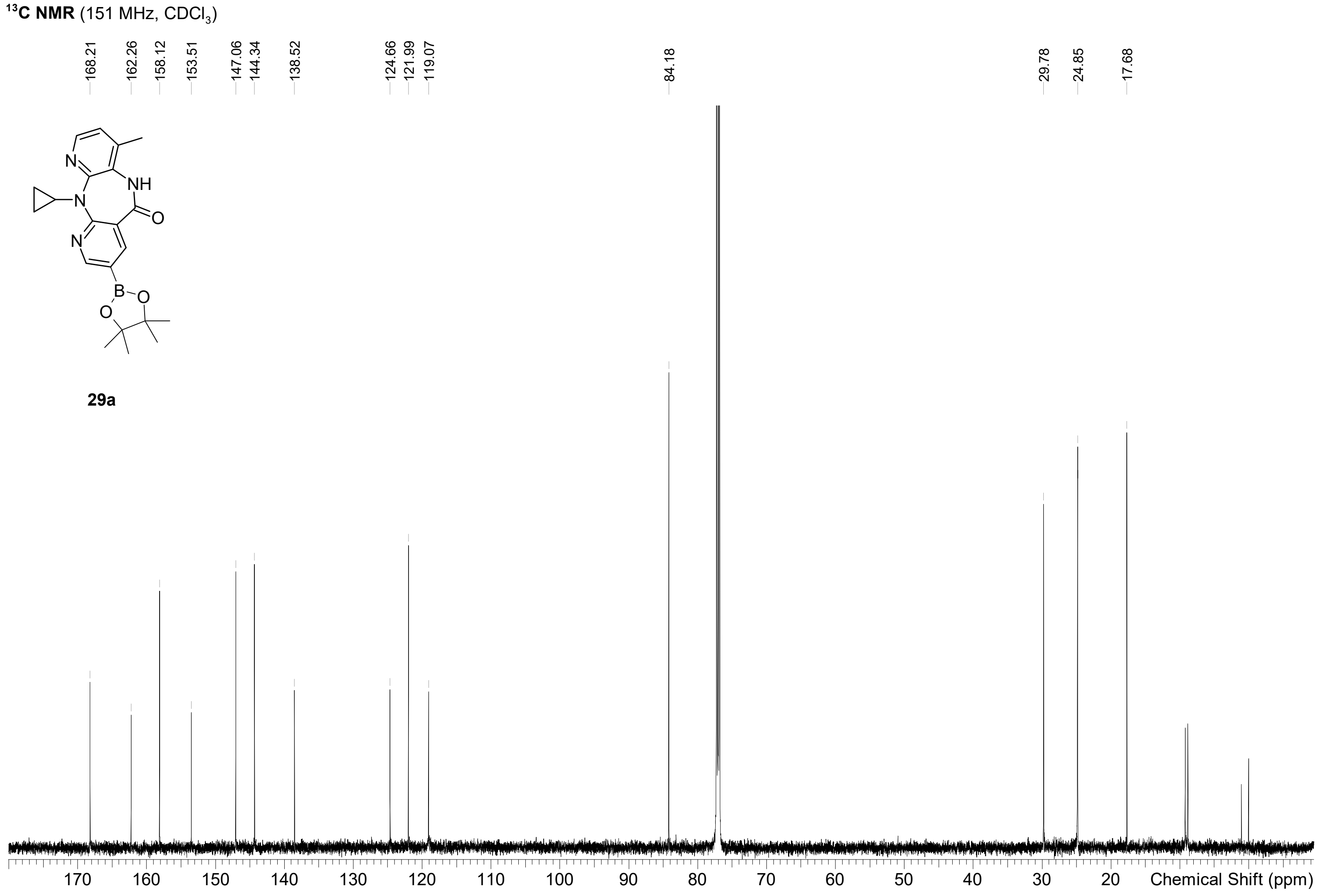

Figure S39: **29a**, ^{13}C-NMR spectra.

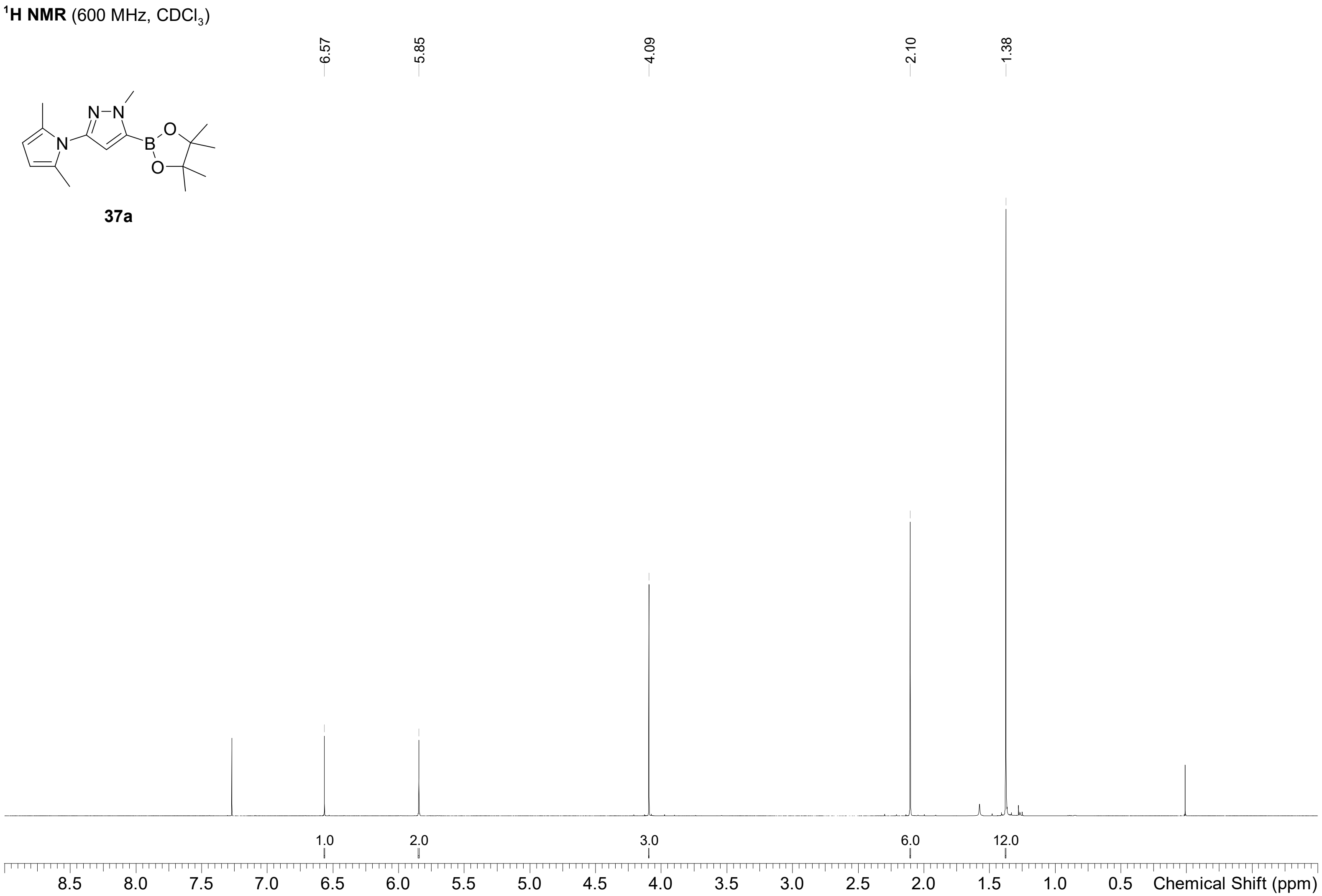

Figure S40: **37a**, ^{1}H-NMR spectra.

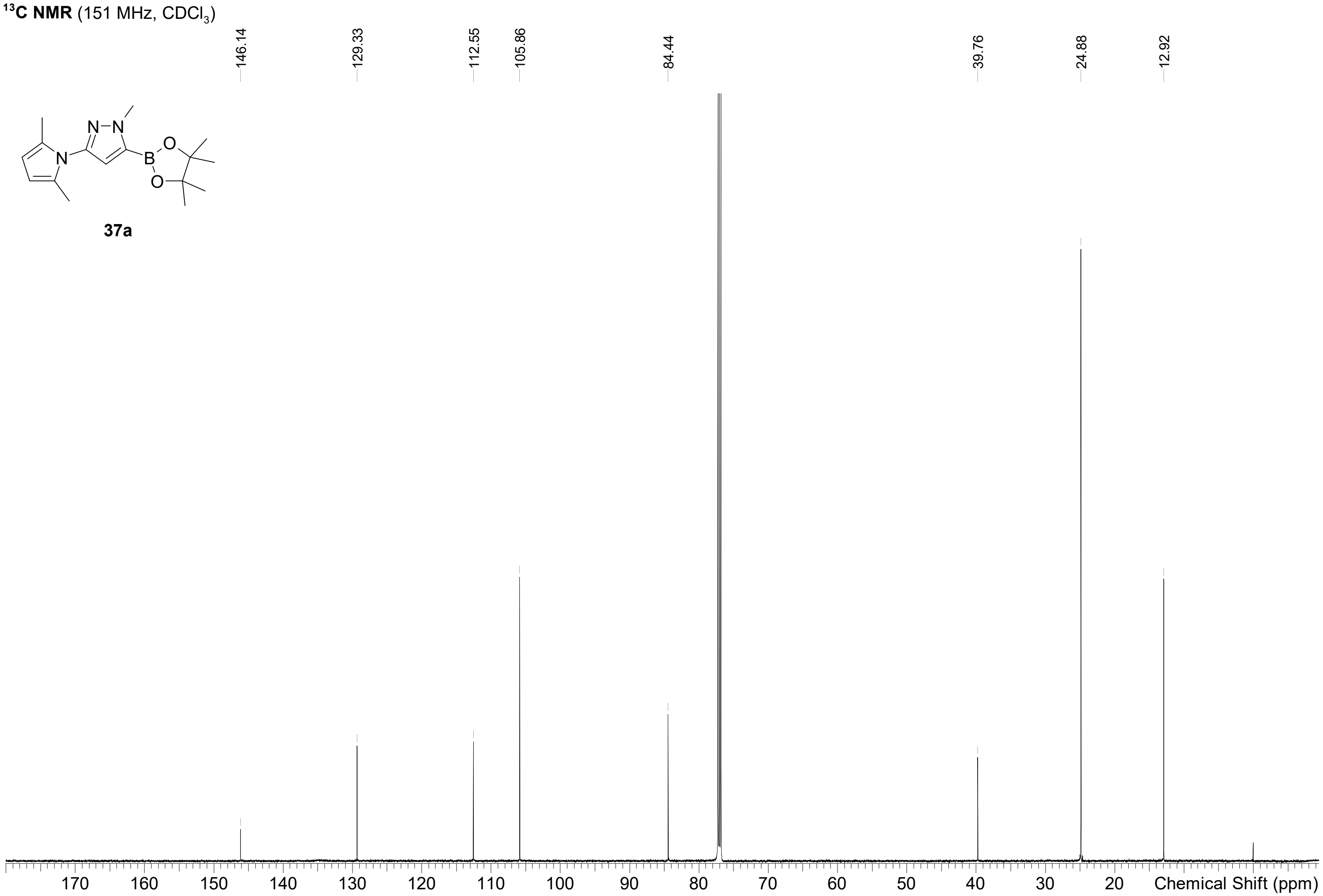

Figure S41: **37a**, ^{13}C-NMR spectra.

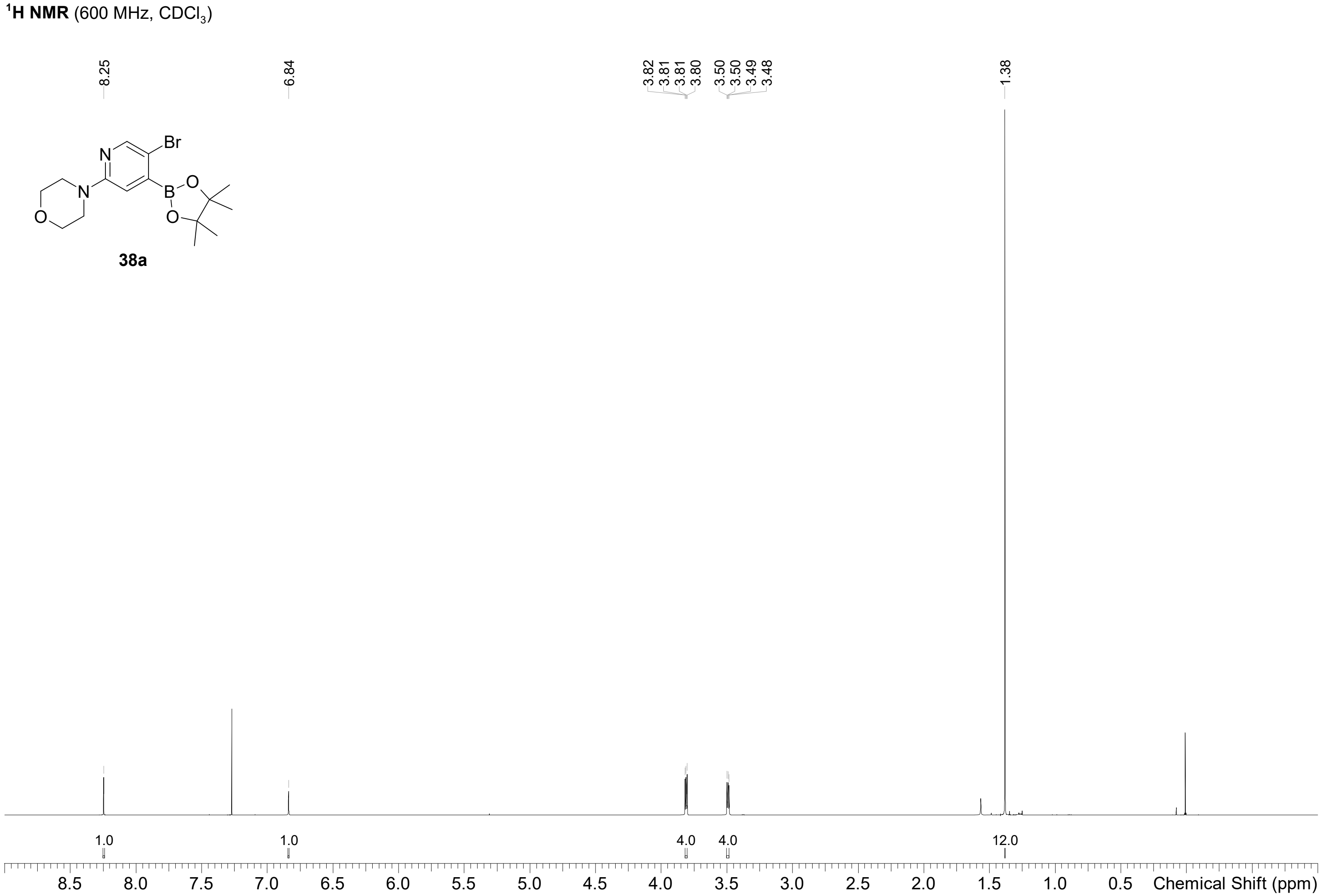

Figure S42: **38a**, ^{1}H-NMR spectra.

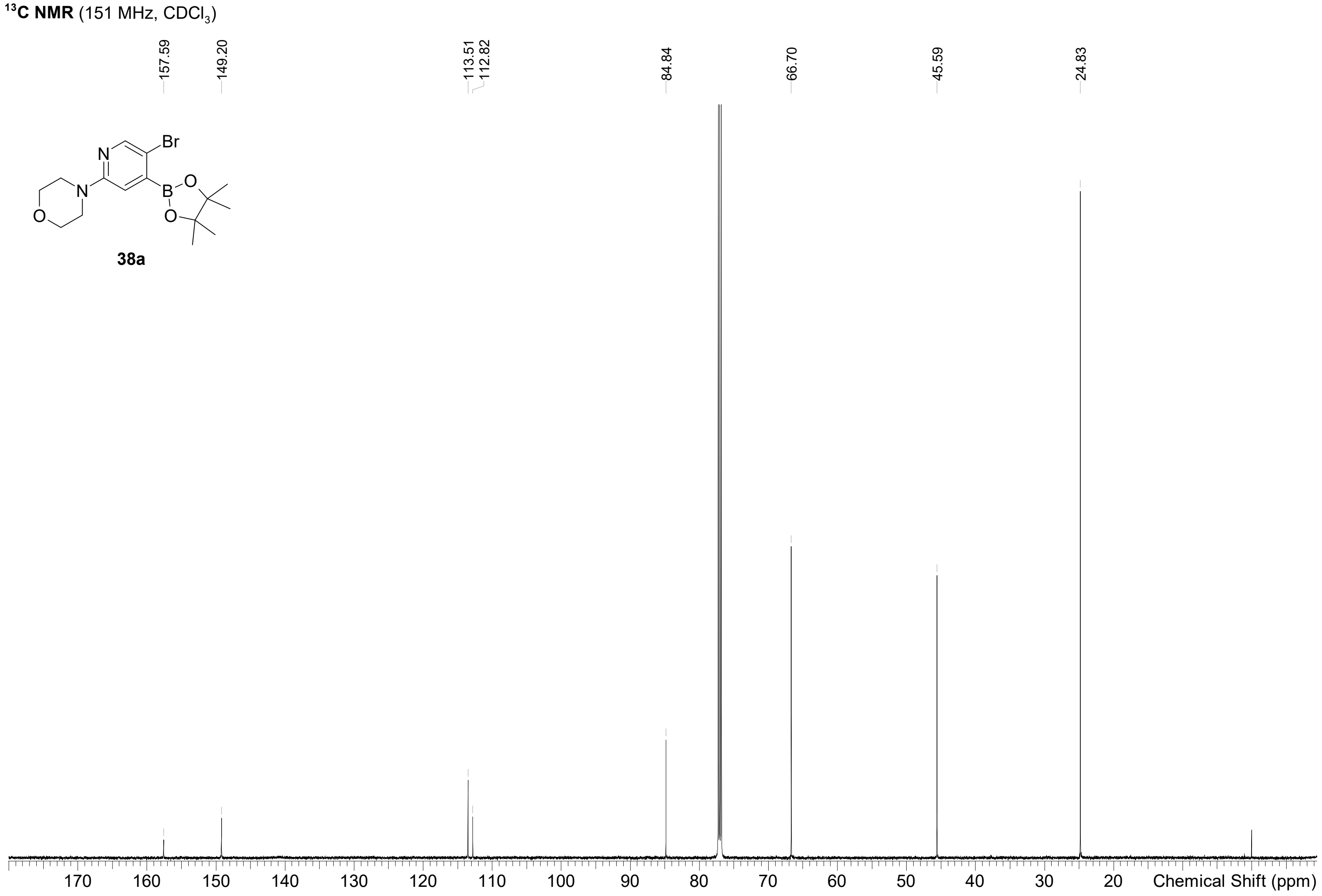

Figure S43: **38a**, ^{13}C-NMR spectra.

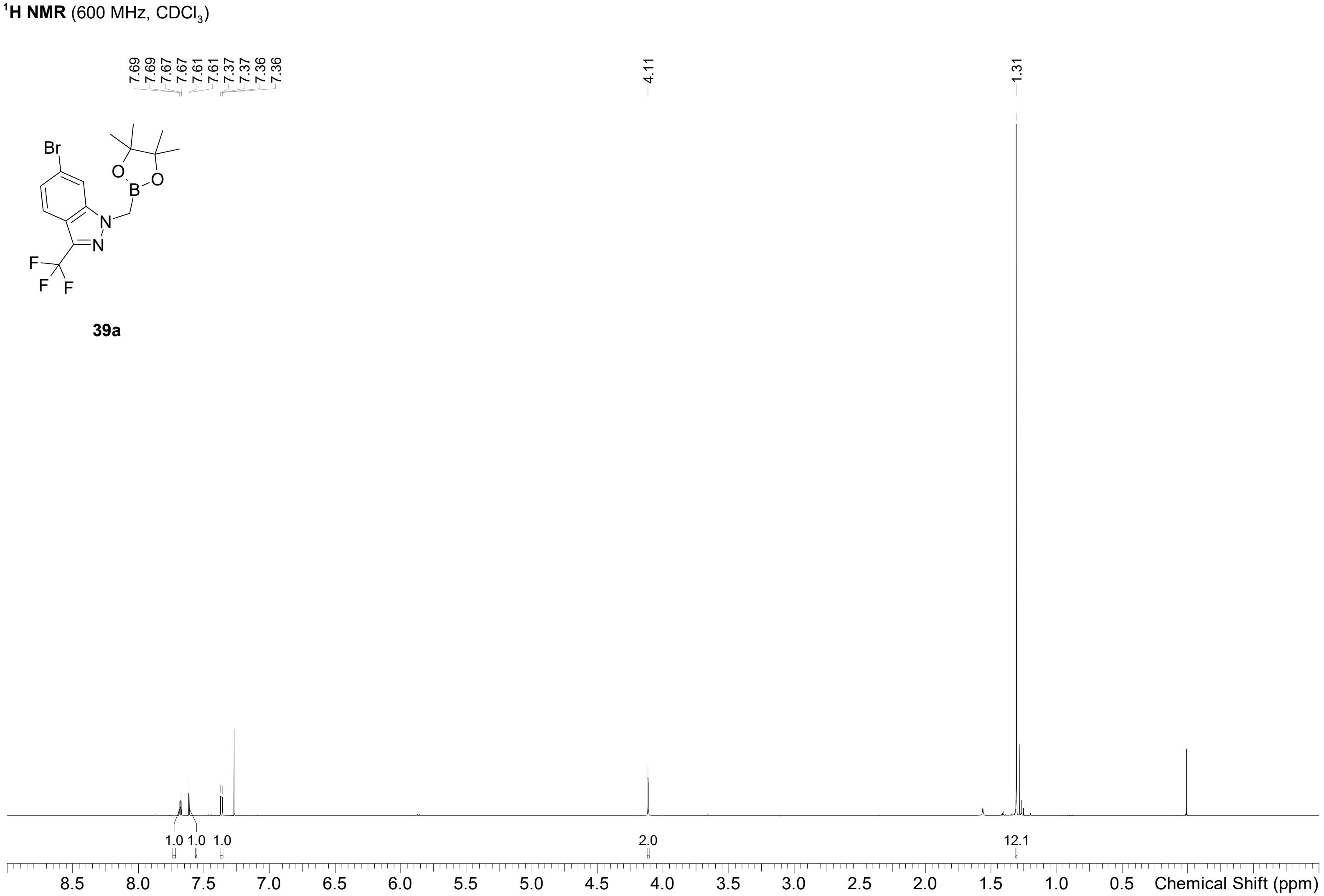

Figure S44: **39a**, ^{1}H-NMR spectra.

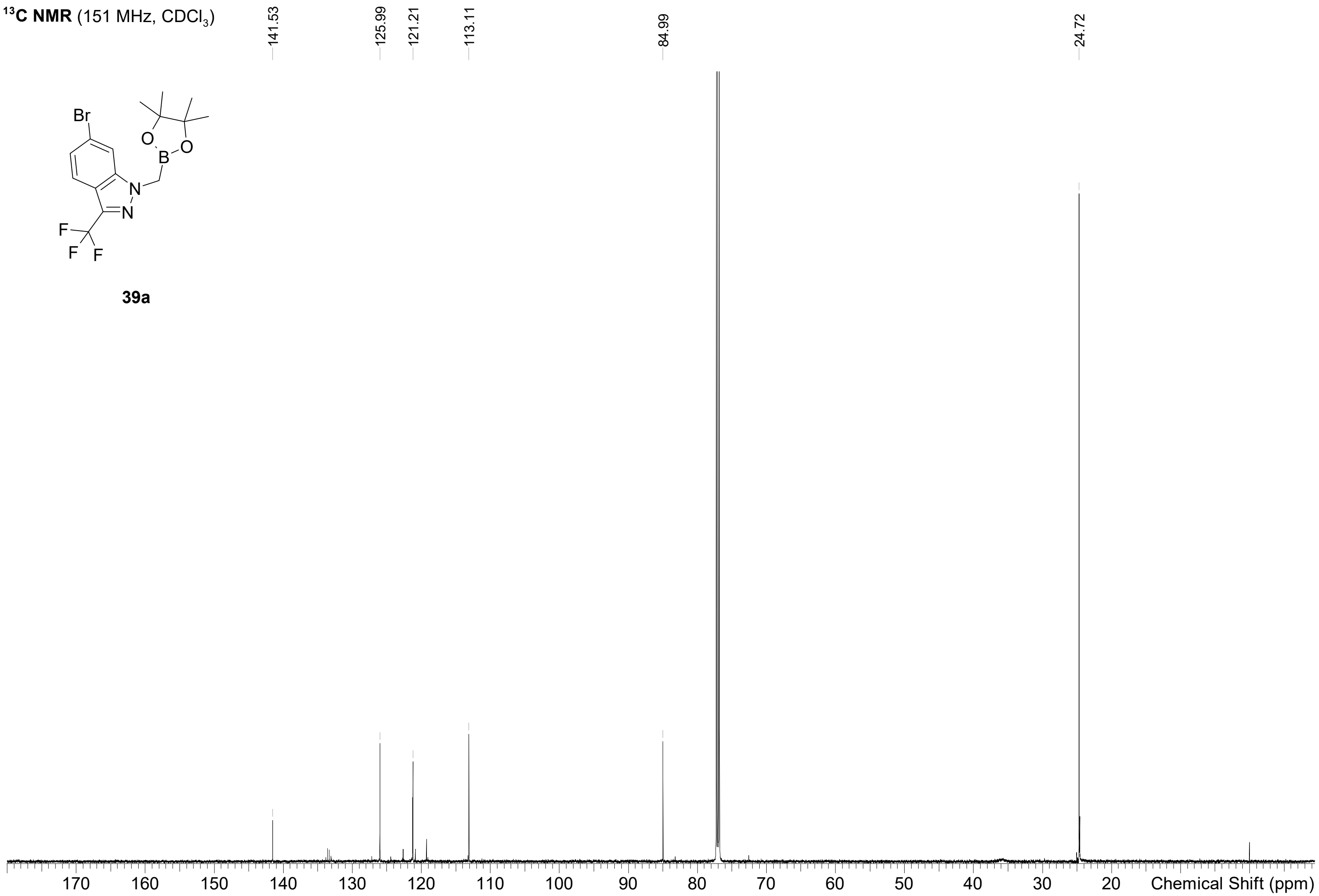

Figure S45: **39a**, ^{13}C-NMR spectra.

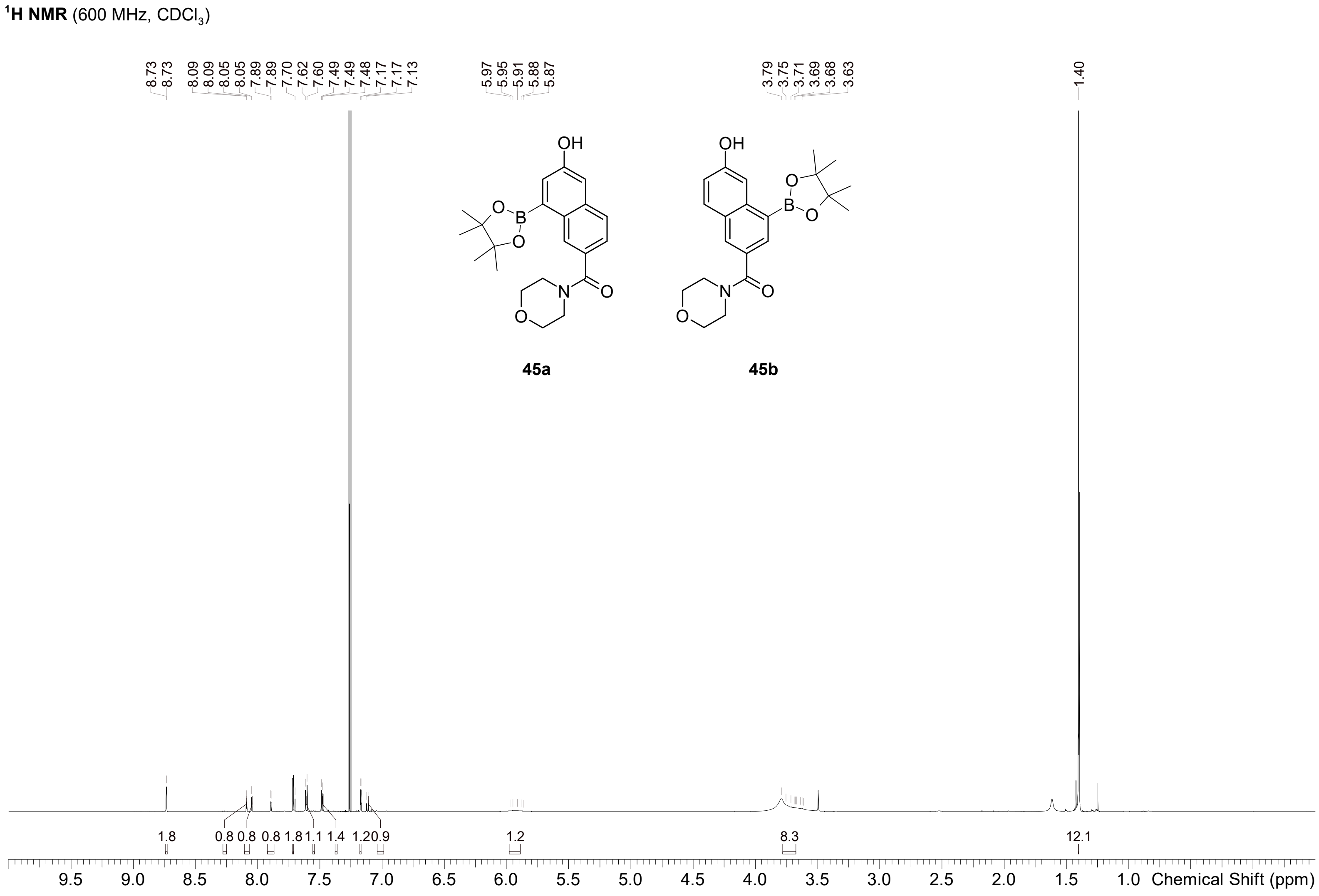

Figure S46: **45a & 45b**, ^{1}H-NMR spectra.

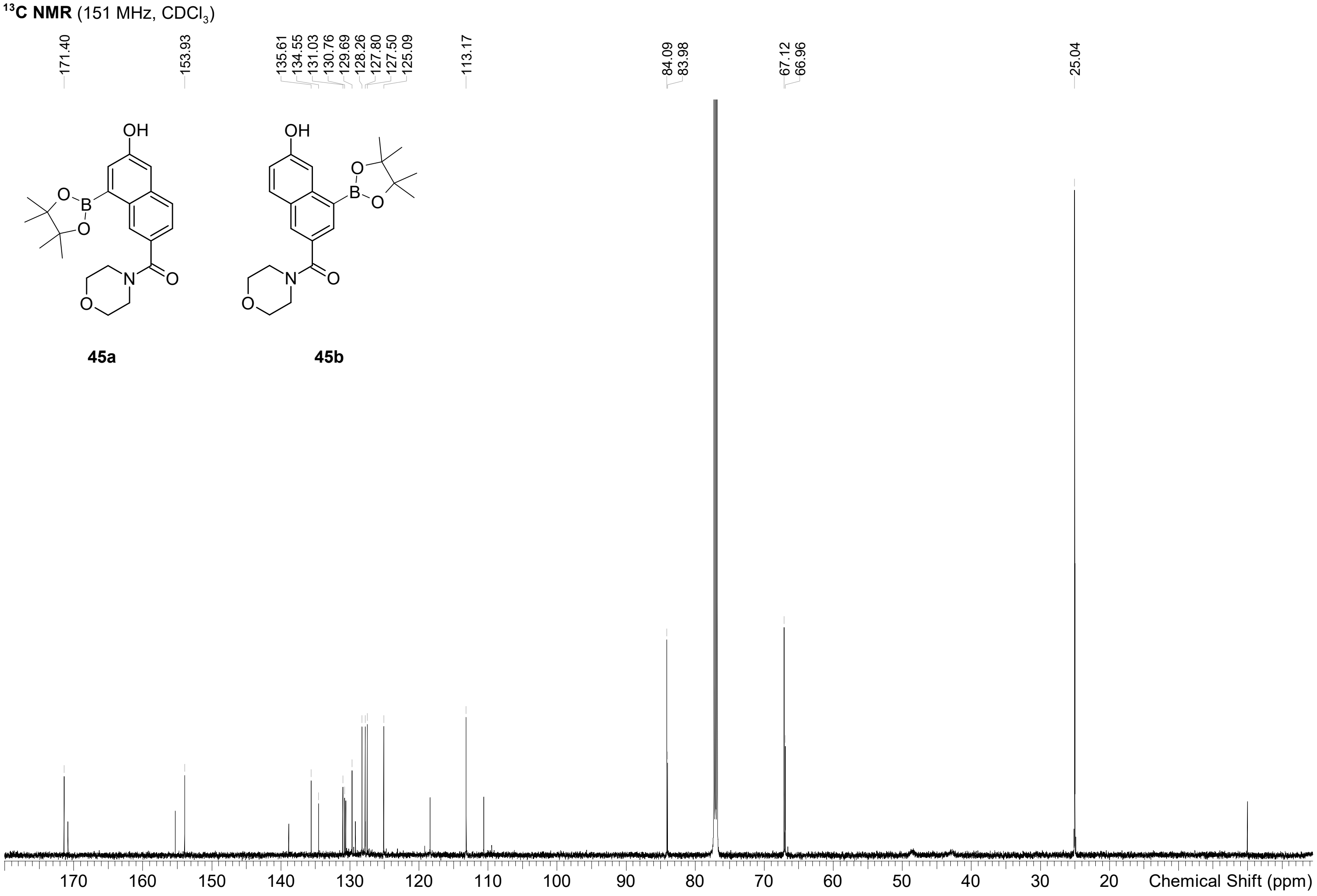

Figure S47: **45a & 45b**, ^{13}C-NMR spectra.

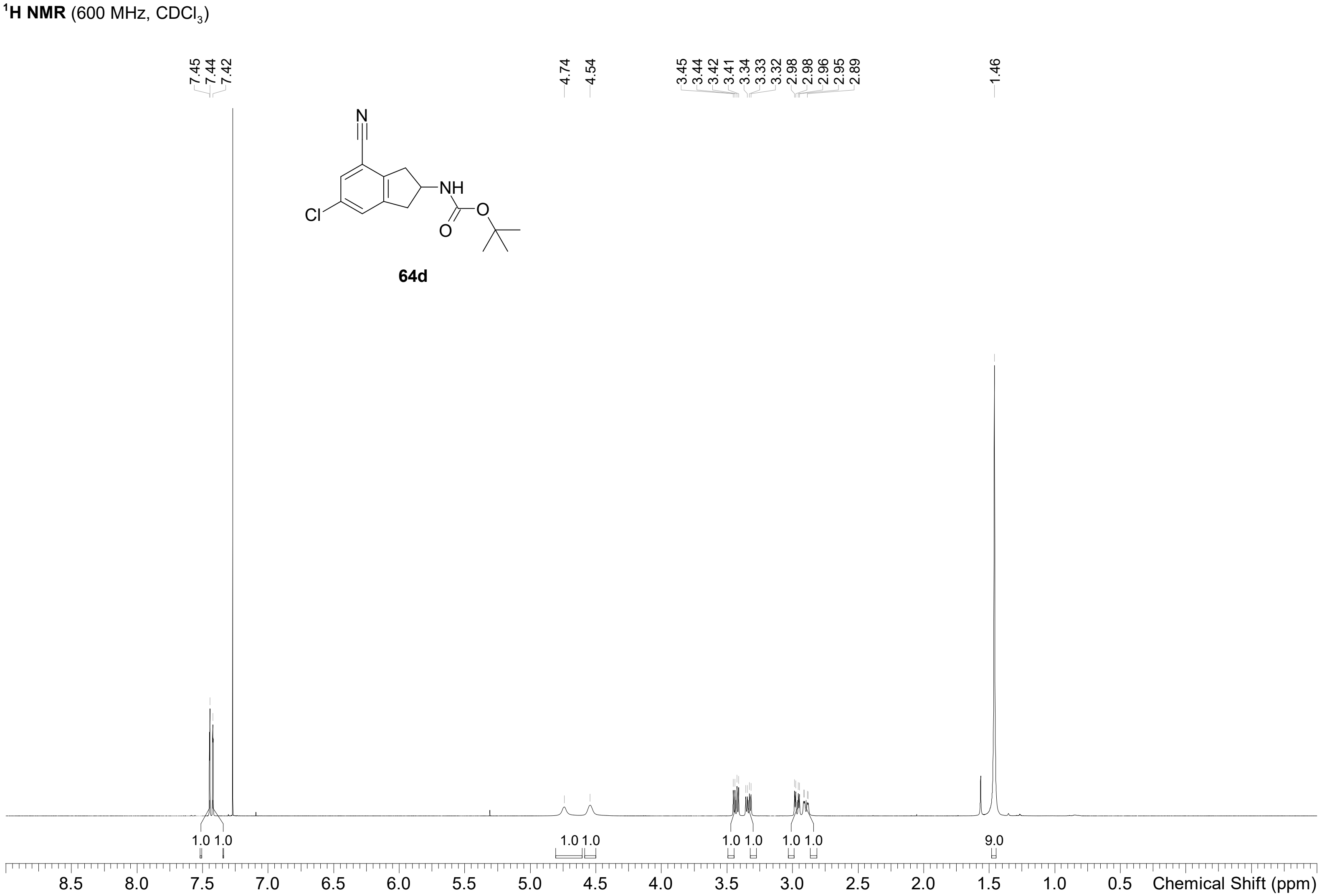

Figure S48: **64d**, ^{1}H-NMR spectra.

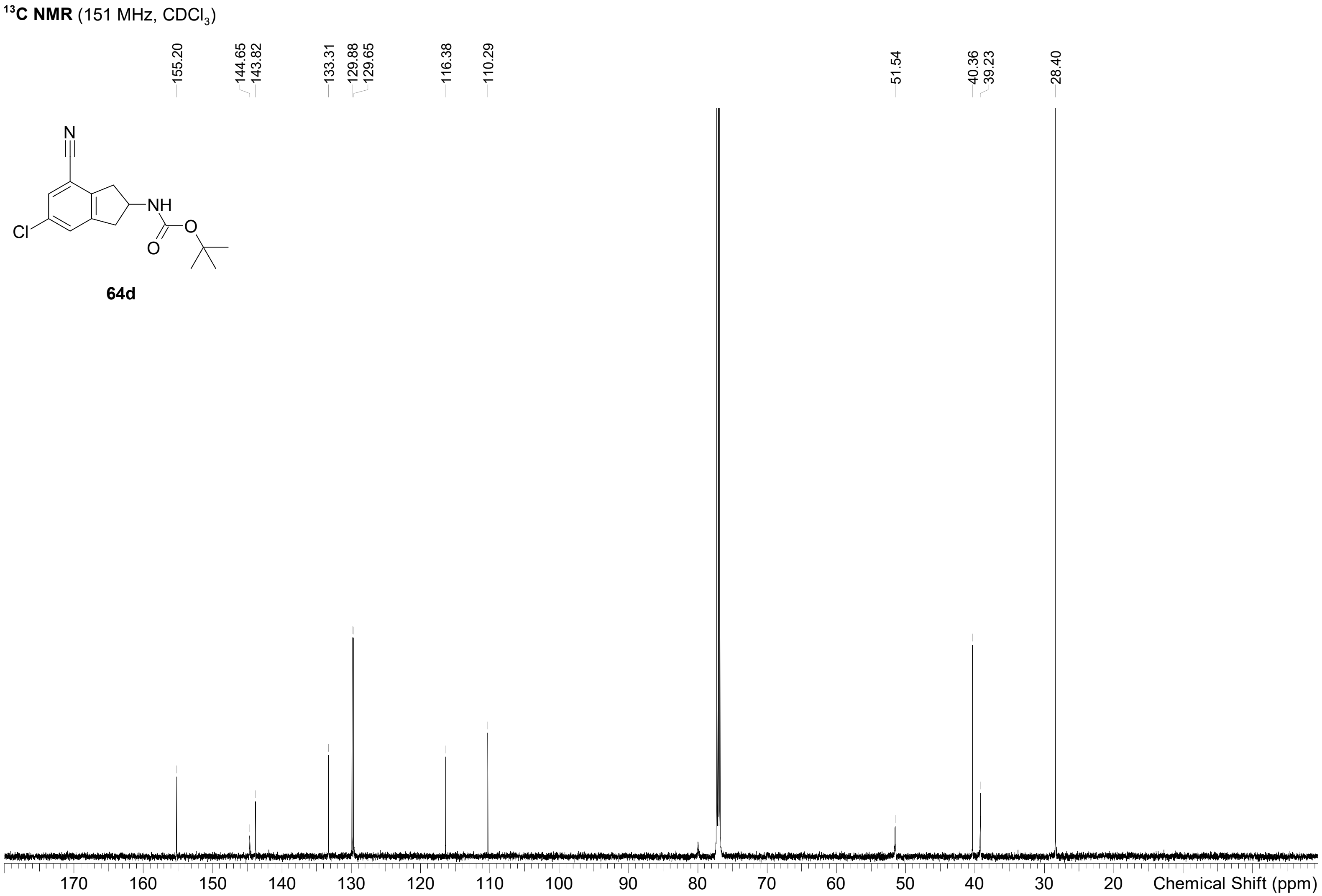

Figure S49: **64d**, ^{13}C-NMR spectra.

References

1. Li, J., Burnham, J. F., Lemley, T. & Britton, R. M. Citation analysis: Comparison of web of science®, scopus™, SciFinder®, and google scholar. *J. Electron. Resour. Med.* **7,** 196–217 (2010).
2. Reyes, R. *et al.* Asymmetric remote C–H borylation of aliphatic amides and esters with a modular iridium catalyst. *Science* **369,** 970–974 (2020).
3. Tian, Y.-M. *et al.* Ni-catalyzed traceless, directed C3-selective C-H borylation of indoles. *J. Am. Chem. Soc.* **142,** 13136–13144 (2020).
4. Yu, X. *et al.* Site-selective alkene borylation enabled by synergistic hydrometallation and borometallation. *Nature Cat.* **3,** 585–592 (2020).
5. Oeschger, R. *et al.* Diverse functionalization of strong alkyl C–H bonds by undirected borylation. *Science* **368,** 736–741 (2020).
6. Larsen, M., Oeschger, R. & Hartwig, J. Effect of ligand structure on the electron density and activity of iridium catalysts for the borylation of alkanes. *ACS Catal.* **10,** 3415–3424 (2020).
7. Lv, J. *et al.* Metal-free directed sp 2-C–H borylation. *Nature* **575,** 336–340 (2019).
8. Iqbal, S. *et al.* Acyl-directed ortho-Borylation of anilines and C7 borylation of indoles using just BBr3. *Angew. Chem. Int. Ed.* **58,** 15381–15385 (2019).
9. Oeschger, R., Larsen, M., Bismuto, A. & Hartwig, J. Origin of the difference in reactivity between Ir catalysts for the borylation of C-H bonds. *J. Am. Chem. Soc.* **141,** 16479–16485 (2019).
10. Bai, S.-T., Bheeter, C. & Reek, J. Hydrogen bond directed ortho-selective C-H borylation of secondary aromatic amides. *Angew. Chem. Int. Ed.* **58,** 13039–13043 (2019).
11. Bisht, R., Hoque, M. & Chattopadhyay, B. Amide effects in C-H activation: Noncovalent interactions with l-shaped ligand for meta borylation of aromatic amides. *Angew. Chem. Int. Ed.* **57,** 15762–15766 (2018).
12. Légaré Lavergne, J., Jayaraman, A., Misal Castro, L., Rochette, É. & Fontaine, F.-G. Metal-free borylation of heteroarenes using ambiphilic aminoboranes: On the importance of sterics in frustrated lewis pair C-H bond activation. *J. Am. Chem. Soc.* **139,** 14714–14723 (2017).
13. Davis, H., Genov, G. & Phipps, R. Meta-selective C-H borylation of benzylamine-, phenethylamine-, and phenylpropylamine-derived amides enabled by a single anionic ligand. *Angew. Chem. Int. Ed.* **56,** 13351–13355 (2017).
14. Chattopadhyay, B. *et al.* Ir-catalyzed ortho-borylation of phenols directed by substrate-ligand electrostatic interactions: A combined experimental/in silico strategy for optimizing weak interactions. *J. Am. Chem. Soc.* **139,** 7864–7871 (2017).
15. Hoque, M., Bisht, R., Haldar, C. & Chattopadhyay, B. Noncovalent interactions in Ir-catalyzed C-H activation: L-shaped ligand for para-selective borylation of aromatic esters. *J. Am. Chem. Soc.* **139,** 7745–7748 (2017).
16. Yin, Q., Klare, H. & Oestreich, M. Catalytic Friedel–Crafts C-H borylation of electron-rich arenes: Dramatic rate acceleration by added alkenes. *Angew. Chem. Int. Ed.* **56,** 3712–3717 (2017).
17. He, J., Shao, Q., Wu, Q. & Yu, J.-Q. Pd(II)-catalyzed enantioselective C(sp3)-H borylation. *J. Am. Chem. Soc.* **139,** 3344–3347 (2017).
18. Obligacion, J., Bezdek, M. & Chirik, P. C(sp2)-H borylation of fluorinated arenes using an air-stable cobalt precatalyst: Electronically enhanced site selectivity enables synthetic opportunities. *J. Am. Chem. Soc.* **139,** 2825–2832 (2017).
19. Li, H., Kuninobu, Y. & Kanai, M. Lewis acid–base interaction-controlled ortho-selective C-H borylation of aryl sulfides. *Angew. Chem. Int. Ed.* **56,** 1495–1499 (2017).
20. Obligacion, J., Semproni, S., Pappas, I. & Chirik, P. Cobalt-catalyzed C(sp2)-H borylation: Mechanistic insights inspire catalyst design. *J. Am. Chem. Soc.* **138,** 10645–10653 (2016).
21. Bisht, R. & Chattopadhyay, B. Formal Ir-catalyzed ligand-enabled ortho and meta borylation of aromatic aldehydes via in situ-generated imines. *J. Am. Chem. Soc.* **138,** 84–87 (2016).
22. He, J. *et al.* Ligand-promoted borylation of C(sp3)-H bonds with palladium(II) catalysts. *Angew. Chem. Int. Ed.* **55,** 785–789 (2016).
23. Furukawa, T., Tobisu, M. & Chatani, N. C-H functionalization at sterically congested positions by the platinum-catalyzed borylation of arenes. *J. Am. Chem. Soc.* **137,** 12211–12214 (2015).
24. Kuninobu, Y., Ida, H., Nishi, M. & Kanai, M. A meta-selective C-H borylation directed by a secondary interaction between ligand and substrate. *Nat. Chem.* **7,** 712–717 (2015).

25. Feng, Y. *et al.* Total synthesis of verruculogen and fumitremorgin a enabled by ligand-controlled C-H borylation. *J. Am. Chem. Soc.* **137,** 10160–10163 (2015).

26. Larsen, M., Wilson, C. & Hartwig, J. Iridium-catalyzed borylation of primary benzylic C-H bonds without a directing group: Scope, mechanism, and origins of selectivity. *J. Am. Chem. Soc.* **137,** 8633–8643 (2015).

27. Wang, G., Xu, L. & Li, P. Double N,B-type bidentate boryl ligands enabling a highly active iridium catalyst for C-H borylation. *J. Am. Chem. Soc.* **137,** 8058–8061 (2015).

28. Saito, Y., Segawa, Y. & Itami, K. Para -C-H borylation of benzene derivatives by a bulky iridium catalyst. *J. Am. Chem. Soc.* **137,** 5193–5198 (2015).

29. Miyamura, S., Araki, M., Suzuki, T., Yamaguchi, J. & Itami, K. Stereodivergent synthesis of arylcyclopropylamines by sequential C-H borylation and Suzuki-Miyaura coupling. *Angew. Chem. Int. Ed.* **54,** 846–851 (2015).

30. Obligacion, J., Semproni, S. & Chirik, P. Cobalt-catalyzed C-H borylation. *J. Am. Chem. Soc.* **136,** 4133–4136 (2014).

31. Larsen, M. & Hartwig, J. Iridium-catalyzed C-H borylation of heteroarenes: Scope, regioselectivity, application to late-stage functionalization, and mechanism. *J. Am. Chem. Soc.* **136,** 4287–4299 (2014).

32. Preshlock, S. *et al.* High-throughput optimization of Ir-catalyzed C-H borylation: A tutorial for practical applications. *J. Am. Chem. Soc.* **135,** 7572–7582 (2013).

33. Liskey, C. & Hartwig, J. Iridium-catalyzed C-H borylation of cyclopropanes. *J. Am. Chem. Soc.* **135,** 3375–3378 (2013).

34. Tajuddin, H. *et al.* Iridium-catalyzed C-H borylation of quinolines and unsymmetrical 1,2-disubstituted benzenes: Insights into steric and electronic effects on selectivity. *Chem. Sci.* **3,** 3505–3515 (2012).

35. Roosen, P. *et al.* Outer-sphere direction in iridium C-H borylation. *J. Am. Chem. Soc.* **134,** 11350–11353 (2012).

36. Dai, H.-X. & Yu, J.-Q. Pd-catalyzed oxidative ortho -C-H borylation of arenes. *J. Am. Chem. Soc.* **134,** 134–137 (2012).

37. Ros, A. *et al.* Use of hemilabile N,N ligands in nitrogen-directed iridium-catalyzed borylations of arenes. *Angew. Chem. Int. Ed.* **50,** 11724–11728 (2011).

38. Robbins, D., Boebel, T. & Hartwig, J. Iridium-catalyzed, silyl-directed borylation of nitrogen-containing heterocycles. *J. Am. Chem. Soc.* **132,** 4068–4069 (2010).

39. Paul, S. *et al.* Ir-catalyzed functionalization of 2-substituted indoles at the 7-position: Nitrogen-directed aromatic borylation. *J. Am. Chem. Soc.* **128,** 15552–15553 (2006).

40. Chotana, G., Rak, M. & Smith, M. Sterically directed functionalization of aromatic C-H bonds: Selective borylation ortho to cyano groups in arenes and heterocycles. *J. Am. Chem. Soc.* **127,** 10539–10544 (2005).

41. Kearnes, S. M. *et al.* The open reaction database. *J. Am. Chem. Soc.* **143,** 18820–18826 (2021).

42. Wilkinson, M. D. *et al.* The FAIR Guiding Principles for scientific data management and stewardship. *Sci. Data* **3,** 1–9 (2016).

43. Murtagh, F. & Legendre, P. Ward's hierarchical agglomerative clustering method: which algorithms implement Ward's criterion? *J. Classif.* **31,** 274–295 (2014).

44. Rogers, D. & Hahn, M. Extended-connectivity fingerprints. *J. Chem. Inf. Model.* **50,** 742–754 (2010).

45. Bhutani, P. *et al.* US FDA approved drugs from 2015–June 2020: A perspective. *J. Med. Chem.* **64,** 2339–2381 (2021).

46. Vitaku, E., Smith, D. T. & Njardarson, J. T. Analysis of the structural diversity, substitution patterns, and frequency of nitrogen heterocycles among US FDA approved pharmaceuticals: miniperspective. *J. Med. Chem.* **57,** 10257–10274 (2014).

47. Gomtsyan, A. Heterocycles in drugs and drug discovery. *Chem. Heterocyc. Compd.* **48,** 7–10 (2012).

48. Miyaura, N. & Suzuki, A. Palladium-catalyzed cross-coupling reactions of organoboron compounds. *Chem. Rev.* **95,** 2457–2483 (1995).

49. Nicolaou, K., Bulger, P. G. & Sarlah, D. Palladium-catalyzed cross-coupling reactions in total synthesis. *Angew. Chem. Int. Ed.* **44,** 4442–4489 (2005).

50. Ertl, P. In silico identification of bioisosteric functional groups. *Curr. Opin. Drug Discov. Dev.* **10,** 281–288 (2007).

51. Ertl, P., Altmann, E. & McKenna, J. M. The most common functional groups in bioactive molecules and how their popularity has evolved over time. *J. Med. Chem.* **63,** 8408–8418 (2020).

52. Ertl, P. An algorithm to identify functional groups in organic molecules. *J. Cheminformatics* **9,** 1–7 (2017).

53. Dalke, A. The chemfp project. *J. Cheminformatics* **11,** 1758–2946 (2019).

54. Landrum, G. *RDKit: Open-source cheminformatics software* May 2010. `http://www.rdkit.org/`.

55. Caldeweyher, E. *et al.* A hybrid machine-learning approach to predict the iridium-catalyzed borylation of C–H bonds. *ChemRxiv preprint* (2022).

56. Meyers, J., Carter, M., Mok, N. Y. & Brown, N. On the origins of three-dimensionality in drug-like molecules. en. *Future Med. Chem.* **8,** 1753–1767 (2016).

57. Pomberger, A. *et al.* The effect of chemical representation on active machine learning towards closed-loop optimization. *React. Chem. Eng.* (2022).

58. Chen, T. & Guestrin, C. *Xgboost: A scalable tree boosting system* in *Proceedings of the 22nd acm sigkdd international conference on knowledge discovery and data mining* (2016), 785–794.

59. Pedregosa, F. *et al.* Scikit-learn: Machine learning in Python. *J. Mach. Learn. Res.* **12,** 2825–2830 (2011).
